# LC-MS/MS Isomeric Profiling of *N*-Glycans Derived from Low-Abundant Serum Glycoproteins in Mild Cognitive Impairment Patients

**DOI:** 10.3390/biom12111657

**Published:** 2022-11-08

**Authors:** Cristian D. Gutierrez Reyes, Md. Abdul Hakim, Mojgan Atashi, Mona Goli, Sakshi Gautam, Junyao Wang, Andrew I. Bennett, Jianhui Zhu, David M. Lubman, Yehia Mechref

**Affiliations:** 1 Department of Chemistry and Biochemistry, Texas Tech University, Lubbock, TX 79409, USA; 2 Department of Surgery, The University of Michigan, Ann Arbor, MI 48109, USA

**Keywords:** MCI, neurological disorder, isomeric *N*-glycans, biomarker glycan

## Abstract

Mild cognitive impairment (MCI) is an early stage of memory loss that affects cognitive abilities, such as language or virtual/spatial comprehension. This cognitive decline is mostly observed with the aging of individuals. Recently, MCI has been considered as a prodromal phase of Alzheimer’s disease (AD), with a 10–15% conversion rate. However, the existing diagnostic methods fail to provide precise and well-timed diagnoses, and the pathophysiology of MCI is not fully understood. Alterations of serum *N*-glycan expression could represent essential contributors to the overall pathophysiology of neurodegenerative diseases and be used as a potential marker to assess MCI diagnosis using non-invasive procedures. Herein, we undertook an LC-MS/MS glycomics approach to determine and characterize potential *N*-glycan markers in depleted blood serum samples from MCI patients. For the first time, we profiled the isomeric glycome of the low abundant serum glycoproteins extracted from serum samples of control and MCI patients using an LC-MS/MS analytical strategy. Additionally, the MRM validation of the identified data showed five isomeric *N*-glycans with the ability to discriminate between healthy and MCI patients: the sialylated *N*-glycans GlcNAc_5_,Hex_6_,Neu5Ac_3_ and GlcNAc_6_,Hex_7_,Neu5Ac_4_ with single AUCs of 0.92 and 0.87, respectively, and a combined AUC of 0.96; and the sialylated-fucosylated *N*-glycans GlcNAc_4_,Hex_5_,Fuc, Neu5Ac, GlcNAc_5_,Hex_6_,Fuc, Neu5Ac_2_, and GlcNAc_6_,Hex_7_,Fuc, Neu5Ac_3_ with single AUCs of 0.94, 0.67, and 0.88, respectively, and a combined AUC of 0.98. According to the ingenuity pathway analysis (IPA) and in line with recent publications, the identified *N*-glycans may play an important role in neuroinflammation. It is a process that plays a fundamental role in neuroinflammation, an important process in the progression of neurodegenerative diseases.

## 1. Introduction

Mild cognitive impairment (MCI) is the term that defines an intermediate stage from normal cognitive function to dementia [1,2]. The concept of MCI is fundamental and essential to the field of aging and neurodegenerative diseases. Patients with MCI have an early stage of memory loss and a decline in their cognitive ability, generating language problems, or affecting visual/spatial perception [3,4,5,6]. Additionally, in many cases MCI may increase the risk of developing more complicated neurological conditions such as Alzheimer’s disease (AD) [7,8]. Thus, an accurate prediction of clinical changes of MCI patients, including qualitative or quantitative alterations at future time points, is essential for early diagnosis of AD and monitoring disease progression.

There has recently been growing concern for predicting the future clinical changes of MCI subjects in progression to AD [5,7,9]. Molecular biomarkers are required to support diagnoses and prognoses that could be used for disease management and monitoring treatment. There is now considerable evidence that protein glycosylation plays a key role in numerous biological functions and changes during the development of a disease [10,11]. Recently, researchers have found great potential in employing glycomics to identify diagnostic biomarkers in circulating biofluids for brain-related diseases [12,13,14,15,16,17,18,19,20]. Additionally, it is well known that glycans have a variety of isomeric conformations where some of the most important structures have differences in the sialic acid linkage (e.g., α2,3/α2,6), fucosylation in the glycan core or branch, and the formation of bisecting *N*-acetylglucosamine in the glycan core [13,21,22]. Glycan isomers were found to be associated with numerous diseases [12,23,24,25] and various cancers [26,27,28]. However, the isomeric glycan profiles of human biofluids such as blood, serum, and cerebrospinal fluid (CSF) from patients with MCI and AD have not been investigated. Therefore, targeted glycomics studies on human blood serum from patients with brain-related diseases are advantageous due to the minimally invasive procedures used for sample acquisition.

The comprehensive characterization of Isomeric glycans in human serum requires the application of a strategy that combines a sensitive mass spectrometry [29] detector with a powerful separation technique. To meet this challenge, our research group recently introduced an in-house developed mesoporous graphitized carbon (MGC) column [30] and a C18 column with a length of 500 mm [31], which can achieve separation of isomeric glycans. With recent advances in mass spectrometry [29] and separation methods, LC-MS/MS has thus been proven to be a reliable and sensitive analytical tool for various omics studies, including glycomics [13,32,33,34]. However, the data observed in MS analysis may have a high false positive rate due to the limited specificity and sensitivity of full scan MS analysis and the analytical variation caused by extensive sample preparation [35,36]. As an alternative, further targeted analysis such as multiple reaction monitoring (MRM), which is focused on a smaller number of predefined analytes and measures the absolute quantity of each target, would be the method of choice for performing the quantitative verification of glycan disease-markers. This technique is performed on a triple quadrupole mass spectrometer with the ability to isolate specific precursor ions, which are subsequently fragmented. In the case of glycomics analysis, the oxonium ions are the most representative glycan fragment ion “transitions” and are therefore used to verify the glycan identity and for glycan quantitation [37,38].

In the present study, we depleted the 14 most abundant human serum proteins of samples derived from patients with a clinical diagnosis of MCI and healthy controls so that we could study mid- to lower-abundance proteins. Then, for the first time, we profiled the isomeric glycome of the low abundant serum glycoproteins by utilizing our sensitive LC-MGC-MS and LC-C18(50 cm)-MS approaches on permethylated *N*-glycans. Additionally, we investigated the expression changes of the isomeric *N*-glycans between control and MCI samples. The MRM verification of the preliminary data showed that five of the isomeric *N*-glycans developed in our analytical strategy provided an improved ability to discriminate between disease states.

## 2. Materials and Methods

### 2.1. Study Participants

Human blood sera were provided from “The Indiana Blood Bank” via the University Hospital in Ann Arbor, Michigan according to IRB approval No. HUM00212084: 10 cases of mild cognitive impairment (MCI) and 10 cases of healthy donors. The patients were clinically diagnosed according to the National Institute of Aging and the Alzheimer’s Association (NIA-AA) [29]. The clinical information associated with the samples used in this study is summarized in Table 1 and fully described in Supplementary Table S1. The control samples were selected as not having a diagnosis of MCI, inflammatory diseases (cirrhosis, hepatitis, pancreatitis, inflammatory bowel disease, ulcerative colitis, Crohns), or any cancer type.

### 2.2. Chemicals and Reagents

PNGase F was obtained from New England Biolabs (Ipswich, MA, USA). Borane-ammonia complex, sodium hydroxide beads, acetic acid, and iodomethane were acquired from Sigma-Aldrich (St. Louis, MO, USA). Dimethyl sulfoxide (DMSO) was bought from Mallinckrodt Chemicals (Phillipsburg, NJ, USA). HPLC grade acetonitrile, water, and isopropanol were purchased from Fisher Scientific (Fair Lawn, NJ, USA). Isolute^®^ C18 (EC) cartridges were purchased from Biotage (Charlotte, NC, USA), and microspin columns were purchased from Harvard Apparatus (Holliston, MA, USA). 

### 2.3. Depletion of High-Abundance Proteins in Serum Samples 

Sera samples from control and MCI patients were depleted of high abundance proteins using a Human 14 Multiple Affinity Removal Column from Agilent Technologies Inc. (Santa Clara, CA, USA). A 30 μL aliquot of serum was depleted according to the protocol provided by the column manufacturer. Thereafter, the collected fractions were concentrated in a spin concentrator 5K MWCO Amicon and resuspended in 50 mM of ammonium bicarbonate (ABC) buffer to perform further analysis. The protein content in the final sample was measured using the Micro BCA^TM^ Protein Assay Kit (Thermo Sc., Rockford, IL, USA).

### 2.4. N-Glycans Release, Purification, Reduction, and Permethylation 

The *N*-glycans were released and extracted according to the procedure described previously [14]. Briefly, 25 μg of protein was diluted to a total volume of 100 μL with 50 mM ABC buffer (pH ~ 7.5) and denatured at 90 °C for 15 min. After the samples had cooled to room temperature, 1 μL of PNGase F (1000 U) was added, and the samples were incubated at 37 °C for 20 h. Then, the denatured samples were dried using a Labconco CentriVap vacuum concentrator (Kansas City, MO, USA). The dried samples were resuspended with 300 μL of 5% acetic acid. The SPE-C18 cartridges were washed with 3 mL of methanol and then equilibrated with 3 mL of 5% acetic acid. The resuspended samples were applied to the SPE-C18 cartridges and washed with 300 μL of 5% acetic acid three times while the flow-through was collected and dried using the vacuum concentrator.

Released and purified *N*-glycans were reduced and permethylated according to the following protocol [14]. The *N*-glycan reduction was accomplished by the addition of 10 μL of 10 μg/μL ammonium-borane solution to the released and purified glycans, followed by incubation at 60 °C for 1 h. After incubation, the residual borane was removed with the addition of methanol, generating methyl borate that was evaporated while drying in the vacuum concentrator. The reduced and dried *N*-glycans were then permethylated with a solid-phase permethylation protocol, as previously described [14]. The dried *N*-glycans were resuspended in 30 μL of DMSO, 1.2 μL of water, and 20 μL of iodomethane. The solution was then applied into a microspin column packed with sodium hydroxide beads that were subsequently incubated in darkness at room temperature for 25 min. After the initial incubation period, an additional 20 μL of iodomethane was applied to the spin column. The reaction was allowed to proceed for an additional 15 min and centrifuged at 1800 rpm. The permethylated *N*-glycans were dried and resuspended in an aqueous solution with 20% acetonitrile containing 0.1% of formic acid for LC-MS analysis.

### 2.5. N-Glycan Profiling

Two previously developed in-house strategies capable of separating an important number of isomeric *N*-glycans were used to complete the isomeric *N*-glycan profile of low-abundant serum glycoproteins in MCI samples. The MGC-LC-MS that uses a home-made 10 mm mesoporous graphitized carbon (MGC) column [30] and the C18(50 cm)-LC-MS that takes advantage of the path length to increase the interactions between analytes and the stationary phase were both used and resulted in isomeric separation [31].

For both the MGC-LC-MS and the C18(50 cm)-LC-MS based chromatographic separation analytical strategies, a glycan sample equivalent to 5 μg derived from serum depleted glycoproteins was introduced in the system as a single injection. The samples were separated on a 10 mm MGC column or a 50 cm C18 column using an UltiMate 3000 Nano UHPLC system (Thermo Sc., San Jose, CA, USA). An Acclaim PepMap 100 C18 trap column was used in both systems (75 μm × 2 cm, 3 μm particle size, Thermo Sc., Pittsburg, PA, USA) for loading and online purification. For the MGC-LC-MS system, the oven was set to an optimum temperature of 75 °C, which has been demonstrated to yield efficient isomeric separation. 

The chromatographic condition of mobile phase A (MPA) was 98% HPLC water and 2% acetonitrile with 0.1% of difluoro acetic acid; mobile phase B (MPB) was 50% isopropanol and 50% acetonitrile with 0.1% of difluoro acetic acid. A multistep gradient with a flow rate of 0.35 μL/min was used for the separation, where MPB was 20% for 10 min in the beginning. It was increased to 50% after 20 min. Later, it was increased to 95% at 60 min and kept constant until 87 min to completely elute the permethylated *N*-glycans. MPB was reduced to 20% at 88 min and was maintained in the same condition until 90 min to equilibrate the column [30]. For the C18(50 cm)-LC-MS system, the oven was set to an optimum temperature of 60 °C. The chromatographic condition of MPA was 98% HPLC water and 2% acetonitrile with 0.1% of formic acid; the MPB was 98% acetonitrile and 2% water with 0.1% of formic acid. A multistep gradient with a flow rate of 0.25 μL/min was used for the separation, where MPB was 20% over 33 min, then ramped to 42% in 4 min. Then, it was gradually increased to 55% after 123 min. Later, it was increased to 9% in 3 min and kept constant for 17 min. Finally, it was decreased to 20% in 3 min and maintained to equilibrate the column [31].

An LTQ Orbitrap Velos (Thermo Sc., San Jose, CA, USA) was used for sample analysis. It was operated in a positive ion mode with an ESI voltage of 1.6 kV. Data was acquired with a scan range of 400–2000 *m/z* and 100 K resolution. The top eight most intense precursor ions were selected for collision induced dissociation (CID) with a normalized collision energy of 35% and activation time of 10 ms. The analysis of the raw data was performed using Xcalibur 4.2 (Thermo Scientific) software; extracted ion chromatograms of each glycan structure, including all possible sodium and ammonium adducts, were generated. The *m/z* values of the identified glycan structures and their corresponding adducts were applied to generate the extracted ion chromatograms (EICs). The areas under the curve of the EICs were integrated, and the generated data were used to perform relative glycan quantitation (see Supplementary Tables S2–S5).

### 2.6. Multiple Reaction Monitoring (MRM) Validation 

The MGC-LC-MS system was used to perform a multiple reaction monitoring (MRM) verification on the previously identified *N*-glycans with statistical differences in abundance between control and MCI samples (*t*-test; *p* value < 0.05). For the MRM data acquisition, the TSQ Vantage (Thermo Sc., San Jose, CA, USA) triple quadrupole mass spectrometer was utilized. A data-dependent acquisition mode (DDA) with two scan events was performed in the positive ion mode. The first event was a full MS scan over the range of 300–1500 *m/z* in Q3 with a scan time of 0.7 s and peak width of 0.7 FWHM. The data-dependent scan occurred for the second scan event where the five most intense ions from scan event one were subsequently selected and subjected to MS^2^. For the MRM mode, three transition ions were summed for each precursor ion to generate the extracted ion chromatogram in Xcalibur Qual Browser. The corresponding transition ions were selected according to our previous publication [37] and are shown in Supplementary Table S6. The peak areas were computed and used to calculate the relative abundance of the investigated targets and subjected to statistical analyses, and they are shown in Supplementary Tables S7 and S8. Statistical *t*-test was applied to the sample groups, after which the Benjamini-Hochberg procedure was utilized to control the false discovery rate (FDR). To investigate the ability of the *N*-glycans to differentiate between sample cohorts, we used receiver operating characteristic (ROC) curves, dot plots, heat maps, principal component analysis (PCA), partial least squares-discriminant analysis (PLS-DA), and orthogonal partial least-squares-discriminant analysis (OPLS-DA) using SIMCA^®^ software version 16.

### 2.7. Protein Analysis of the Depleted Serum Samples

The equivalent to 50 μg of protein from the depleted serum was resuspended to 50 μL with 50 mM NH_4_HCO_3_ buffer and denatured at 90 °C in a water bath for 10 min. The denatured protein was reduced by the addition of 1.25 μL of dithiothreitol (DTT) 200 mM and incubation at 60 °C for 45 min. Then, the denatured and reduced protein was alkylated with the addition of 5 μL of iodoacetamide (IAA) 200 mM and incubation at 37 °C for 45 min. A second addition of 1.25 μL of DTT 200 mM and incubation at 37 °C for 30 min was performed to quench the IAA excess. Trypsin enzyme was added to the treated sample at a concentration ratio of 1:25 and incubated at 37 °C overnight. Finally, the samples were dried down in a SpeedVac concentrator.

The proteomics analysis was performed with the LC-MS/MS (Thermo Ultimate 3000 nano system coupled with a Q Exactive HF). The online purification was performed using a C18 Acclaim PepMap trapping column (75 μm I.D. × 2 cm, 3 µm particle sizes, 100 Å pore sizes, Thermo Scientific, San Jose, CA, USA). Peptide separation was achieved using a C18 Acclaim PepMap RSLC column (75 µm I.D. × 15 cm, 2 µm particle sizes, 100 Å pore sizes, Thermo Scientific, San Jose, CA, USA). The equivalent of 2 μg of proteins was injected for analysis under 120 K resolution. A 120 min LC method was used for the separation of the digested peptide sample. Briefly, the LC gradient of solvent B in LC-MS/MS analysis was 2% over 10 min, 2–20% over 55 min, 20–30% over 25 min, 30–50% over 20 min, 50–80% over 1 min, 80% over 4 min, 80–5% over 1 min, and 5% over 4 min. Two scan events were applied for the data-dependent acquisition. The first scan event was a full MS scan at a resolution of 120 K. The second scan event was an HCD MS/MS scan using a normalized collision energy (CE) of 35% and 10 ms of activation time. Proteomics data was then searched against the UniProt database homo sapiens in MaxQuant^®^ (Max Planck Institute of Biochemistry, Planegg, Germany). The protein relation with MCI and AD was investigated using ingenuity pathway analysis (IPA) and GeneOntology (GO).

## 3. Results

### 3.1. Analytical Workflow

**Figure 1:**
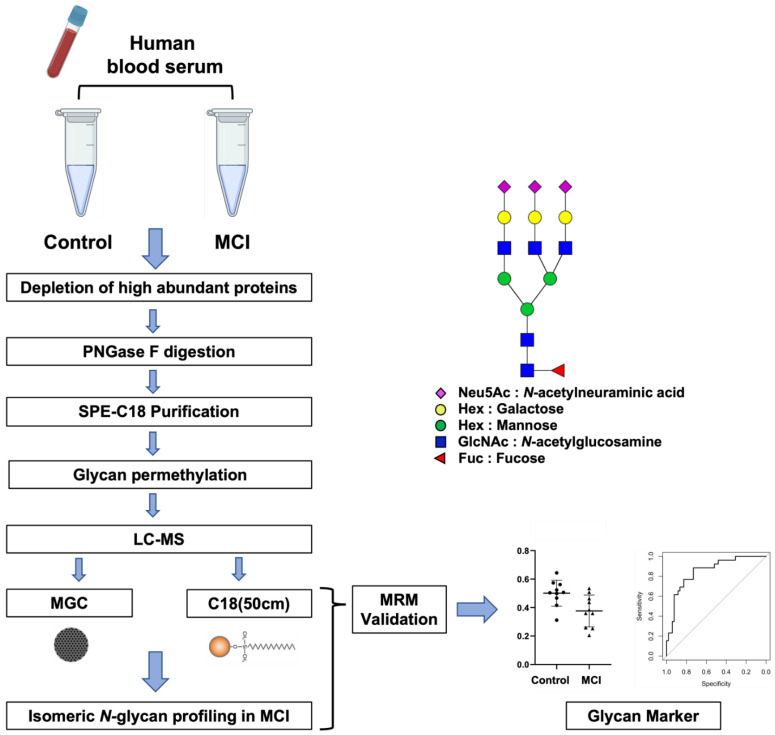
Workflow for the LC-MS analysis and glycan nomenclature. A four-digit *N*-glycan nomenclature was used in the next order; *N*-acetylglucosamine, hexose, fucose, and *N*-acetylneuraminic acid (GlcNAc, Hex, Fuc, Neu5Ac).

Serum samples were collected from 10 patients with a clinical diagnosis of MCI (3 females and 7 males, a mean age of 74 years [SD ± 8], and a ratio of smoker:non-smoker 3:7) and 10 healthy controls (2 females and 8 males, a mean age of 73 years [SD ± 10], and a ratio of smoker:non-smoker 4:6). Initially, the sera samples were subjected to depletion using a Human 14 Multiple Affinity Removal Column. This column depletes the fourteen high abundant human serum proteins-glycoproteins, namely albumin, IgG, antitrypsin, IgA, transferrin, haptoglobin, fibrinogen, alpha2-macroglobulin, alpha1-acid glycoprotein, IgM, apolipoprotein AI, apolipoprotein AII, complement C3, and transthyretin (Agilent Technologies Inc.). The removal of these proteins improves subsequent LC-MS analysis by effectively expanding the dynamic range of the low-abundant serum proteins. It also enables the characterization and investigation of low-abundant serum glycoproteins and their components [39]. Thus, an LC-MS approach was applied for the quantitation of isomeric *N*-glycans released from low-abundant serum glycoproteins to characterize the glycome alterations between control and MCI patients. The experimental workflow is shown in Figure 1. The depleted sample was denatured at 95 °C for 10 min. Then, the glycans were enzymatically released from the denatured glycoproteins and separated from the remaining protein material using SPE-C18 cartridges. The purified glycans were reduced and permethylated to enhance the ionization efficiency of the LC-MS analysis [14].

Two previously developed techniques capable of separating isomeric *N*-glycan forms were used to complete the isomeric *N*-glycan profile of low-abundant serum glycoproteins in the control and MCI samples: the MGC-LC-MS that uses a home-made mesoporous graphitized carbon (MGC) column of 10 mm length [30] and the C18(50 cm)-LC-MS that takes advantage of the path length to increase the interactions of the analyte-stationary phase resulting in isomeric separation [31]. Both techniques have been capable of separating all types of isomeric *N*-glycans including high mannose, fucosylated, and sialylated. For glycan identification, their theoretical *m/z* values representing the protonated, ammoniated, or sodiated molecular ions with a mass tolerance of 10 ppm were selected. The peaks were assigned and integrated manually, where subsequently all the areas were normalized by the total peak area. Thereafter, the *N*-glycan profiles of both analytical strategies were compared and investigated. To simplify the annotation of glycan structures, a four-digit nomenclature was employed as in Figure 1, where each digit represents the number of monosaccharides associated with an *N*-glycan structure in the following order: *N*-Acetylglucosamine, Hexose, Fucose, and *N*-Acetylneuraminic acid (GlcNAc, Hex, Fuc, Neu5Ac).

### 3.2. MGC-LC-MS and C18(50 cm)-LC-MS Comparison 

**Figure 2:**
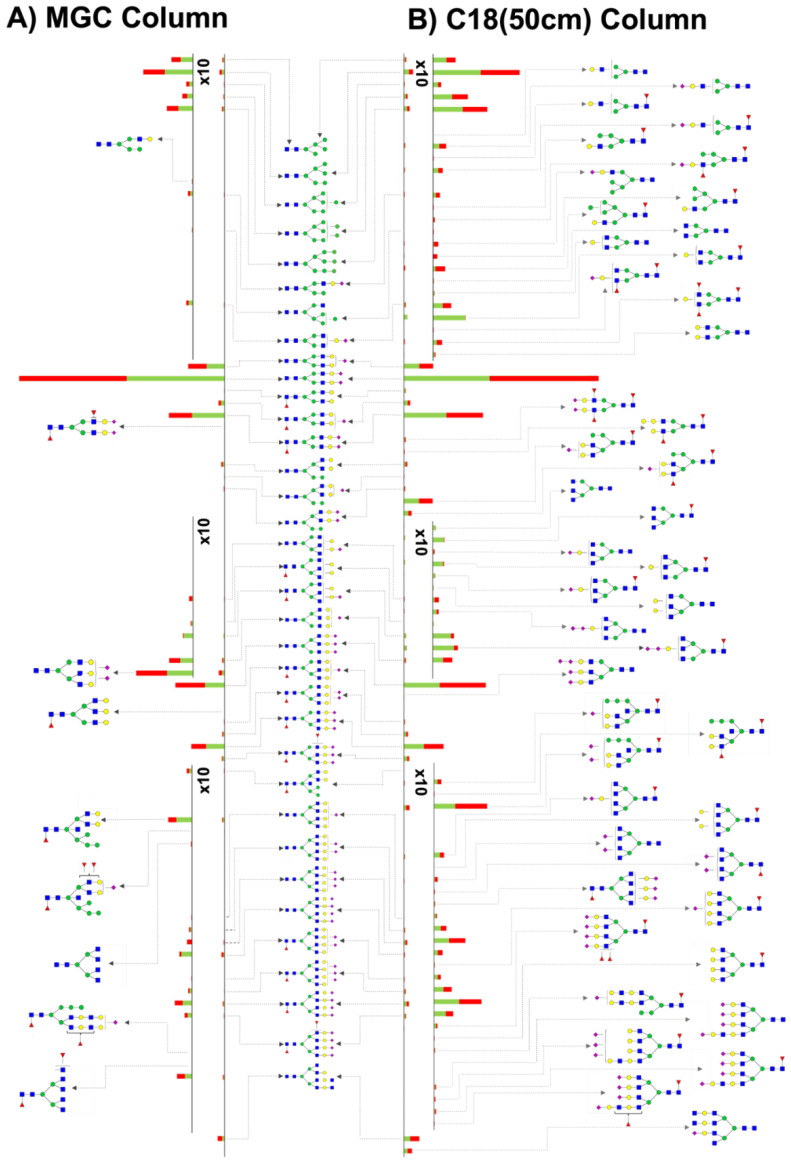
Butterfly plot comparison of the *N*-glycan profiles observed for the for MGC-LC-MS (**A**), C18(50 cm)-LC-MS (**B**) analysis; the green color represents the abundance of control samples and the red color the abundance of the MCI samples. Glycan identification as in Figure 1.

Two different LC-MS systems were used to investigate the glycome differences between the extracted low-abundant glycoproteins from control and MCI serum samples, resulting in an extensive isomeric *N*-glycan profile. First, we analyzed the samples using an MGC-LC-MS approach that was recently developed in our research laboratory [30] and demonstrated isomeric separation of permethylated *N*-glycans released from standard glycoproteins and cancer cell lines. This approach was applied for the first time for the evaluation of disease samples. We were able to accurately identify and quantify a total of 84 isomeric *N*-glycan structures that corresponded to 45 *N*-glycan conformations based on PNGase F digestion from low-abundant serum glycoproteins depleted from control and MCI samples, as in Supplementary Tables S2 and S3. The representative total ion chromatogram (TIC) is shown in Supplementary Figure S1A. Secondly, we analyzed the samples using a C18(50 cm)-LC-MS approach that was recently utilized by our research laboratory [31] to investigate the separation of permethylated isomeric *N*-glycans digested from standard glycoproteins. This approach was also applied for the first time for the evaluation of disease samples. We were able to accurately identify and quantify a total of 109 isomeric *N*-glycan structures that corresponded to 80 *N*-glycan conformations from the PNGase F digested low-abundant serum glycoproteins depleted from the control and MCI samples (Supplementary Tables S4 and S5). The representative TIC is shown in Supplementary Figure S1B. Figure 2 shows a butterfly plot comparing the observed *N*-glycans using the MGC column (Figure 2A) and the C18(50 cm) column (Figure 2B).

**Figure 3:**
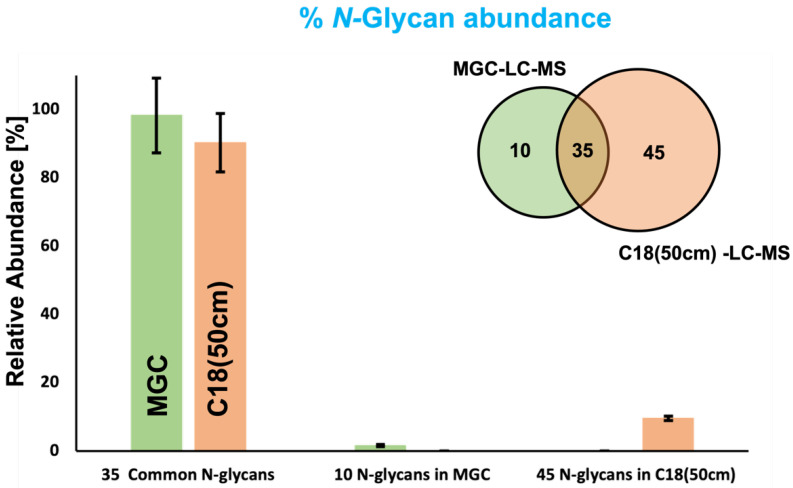
Bar graph compares the % glycan abundance between techniques, and the Venn diagram compares the number of *N*-glycans detected for each strategy.

A Venn diagram (Figure 3) was used to compare the common glycans between the investigated analytical techniques. The diagram describes 35 common *N*-glycans between the C18(50 cm)-LC-MS and the MGC-LC-MS strategies; 45 and 10 *N*-glycans were observed only in the C18(50 cm)-LC-MS and the MGC-LC-MS approaches, respectively. The bar graph of Figure 3 shows the 15 top abundant *N*-glycans that were common in both systems, where these glycan compositions represented 85% and 95% of the total abundance observed using the C18(50 cm) and MGC columns, respectively. The 10 unique *N*-glycans for the MGC approach represent only 1.6% of the total relative abundance, and the 45 unique *N*-glycans of the C18 approach represent only 9.6% of the total relative abundance. According to the results, we were able to identify and quantify 80 *N*-glycans using the C18(50 cm)-LC-MS strategy, a large number in comparison to the 45 identified using the MGC-LC-MS strategy. Conversely, the MGC column showed better capabilities for isomeric separation with a total of 39 isomers, versus only 29 using the C18(50 cm) column.

Supplementary Figures S2 and S3 show the TICs of all the detected isomeric *N*-glycans for the MGC and C18(50 cm) approaches, respectively. The TIC comparison between both strategies shows a superior capability of the MGC column to separate isomeric *N*-glycan forms. The MGC column resolved three chromatography peaks for the glycan structure 4-5-1-1, while only two peaks were resolved for the C18(50 cm) column. Similar results were observed for the glycan structures 4-5-1-2, 5-6-1-2, 5-6-0-3, and 6-7-1-4. Interestingly, some of the resolved peaks of the above-mentioned isomeric glycans showed important statistical differences between the control and MCI samples investigated in this approach; results are further described in Section 3.3. With respect to retention time, the C18(50 cm) column followed the expected patterns [40,41,42]. The high mannose glycans consecutively eluted according to the number of mannose units attached to the glycan with intervals of 13 min by an extra mannose unit. The sialylated and sialylated-fucosylated glycans consecutively eluted according to the antenna and sialic acid numbers attached in the glycan structure. For the MGC column, all the high mannose glycans detected were the initial eluted compounds with retention times between 31 and 34 min. Due to the large number of isomers observed for the sialylated and sialylated-fucosylated glycans, it was complicated to define an elution order for these glycan types. Similar to the C18(50cm) column, the sialylated and sialylated-fucosylated glycans eluted according to the antenna number (di-, tri-, and tetra-), but for the MGC column, the glycans with the largest number of sialic acids eluted first. The MGC elution times correlated with the observed for similar PGC approaches as was described for Gautman et al. [30].

**Figure 4:**
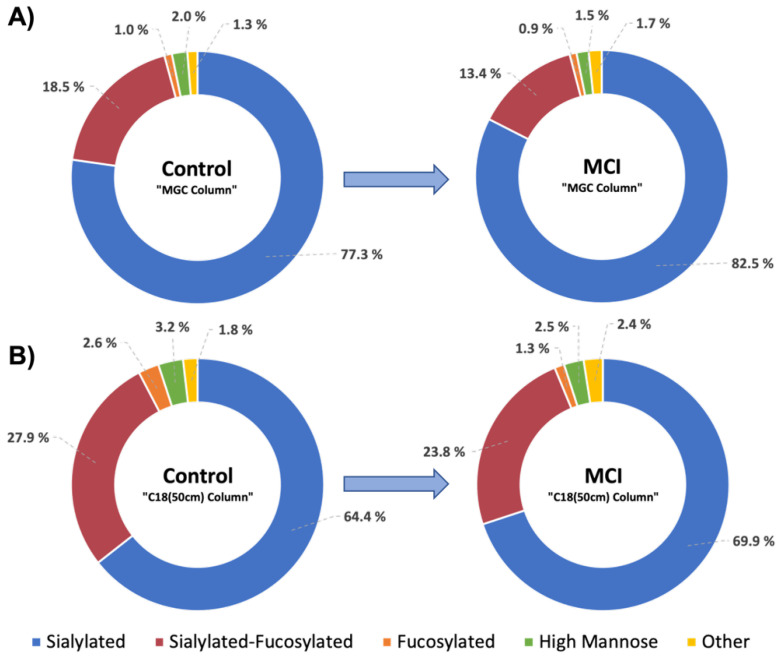
Changes in the glycome abundance of control and MCI samples. (**A**) MGC-LC-MS and (**B**) C18(50cm)-LC-MS approaches.

The glycome changes associated with group-glycan types was also investigated using doughnut graphs. For comparison, the data was separated into the following main glycan groups: sialylated, sialylated-fucosylated, fucosylated, high mannose, and others. Regardless of the column differences and the number of identified glycans in each strategy, the changes in glycan abundances between control and MCI samples was constant (Figure 4). In the progression of control to MCI patients, we observed an increase of about 5.4% in the abundance of sialylated glycans, a decrease of 4.6% in the abundance of sialylated-fucosylated glycans, a decrease of 0.7% in the abundance of fucosylated glycans, a decrease of 0.6% in the abundance of high mannose glycans, and an increase of 0.5% in the abundance in other type of glycans. 

### 3.3. Differentially Expressed Relative Abundance of N-Glycans Derived from Low Abundant Serum Glycoproteins in Control and MCI Samples 

Due to its superior ability to separate isomeric *N*-glycans, the MGC-LC-MS approach was utilized in the MRM mode to complete a target analysis of twelve *N*-glycans with statistical differences in abundance between the control and MCI samples (*p* value < 0.05) found in the identification stage. The targeted glycans can be observed in Supplementary Table S6. After the MRM analysis was completed, the peak areas were computed and used to calculate the relative abundance of the glycan targets. A statistical *t*-test was applied to the sample groups and the Benjamini-Hochberg correction was utilized to control the FDR. The ability of the statistically significant *N*-glycans (*p* values < 0.05) to differentiate between control and MCI samples was investigated, as shown in Table 2. 

**Figure 5:**
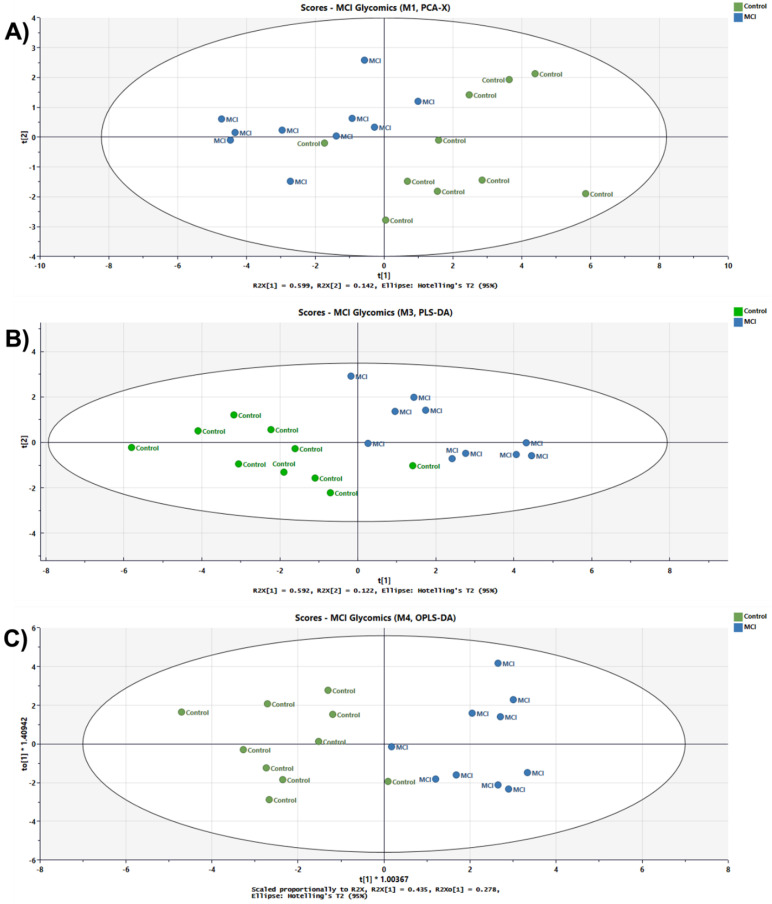
Score plots comparing glycomics patterns from control and MCI patients. (**A**) PCA, R2X[1] = 0.599, R2X[2] = 0.142, Ellipse Hotelling’s T2 95% CI. (**B**) PLS-DA, R2X[1] = 0.592, R2X[2] = 0.122, Ellipse Hotelling’s T2 95% CI. (**C**) OPLS-DA, Scaled proportionality to R2X, R2X[1] = 0.435, R2X_0_[1] = 0.278, Ellipse Hotelling’s T2 95% CI.

To find the *N*-glycans with differential expression between non-disease and disease state the sample cohorts were compared using unsupervising PCA, which revealed a degree of natural separation (Figure 5A). Then, PLS-DA and OPLS-DA analyses were used to validate the glycome differences between sample cohorts (Figure 5B,C, respectively).

**Figure 6:**
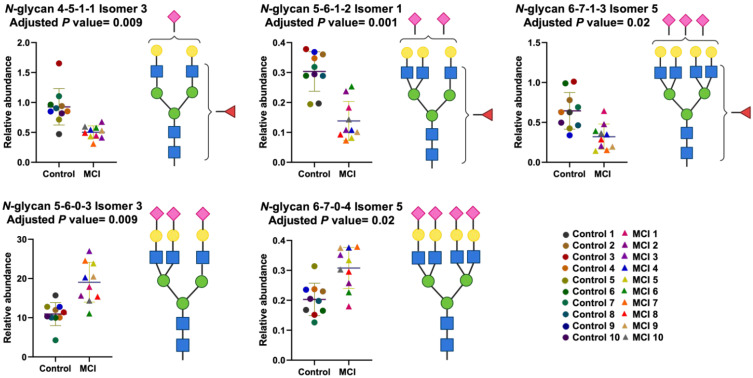
Dot plots and figures of the *N*-glycans with statistically significant differences between control and MCI samples. Glycan nomenclature: GlcNAc, Hex, Fuc, NeuAc (*N*-acetylglucosamine, Hexose, Fucose, *N*-acetylneuraminic acid).

The isomeric separation developed in our analytical strategy showed clear glycome differences between the control and the MCI samples. Five *N*-glycans were found to have the most statistically significant differences in abundance between the tested cohorts (*p* values < 0.05), Table 2: a tri-antennary trisialylated glycan with an adjusted *p* value of 0.009 (5-6-0-3 Isomer 3); a tetra-antennary trisialylated glycan with an adjusted *p* value of 0.02 (6-7-0-4 Isomer 5); and three sialylated mono-fucosylated glycans with one to three sialic acids (4-5-1-1 Isomer 3, the 5-6-1-2 Isomer 1, and the 6-7-1-3 Isomer 5) and respective adjusted *p* values of 0.009, 0.001, and 0.02. Thus, we further investigated the performance of the isomeric *N*-glycans to differentiate the disease state using dot plots and ROC curves as shown in Figure 6. The dot plots show an increase in abundance of the sialylated glycans in the progression of healthy to MCI patients. Conversely, the sialylated fucosylated glycans decrease in abundance in the progression of healthy to MCI patients. These results correlated with the general glycan profile described in Figure 4 and the heat map reported as Supplementary Figure S4. The heat map clearly showed up-regulation for most of the sialylated *N*-glycans and down-regulation for most of the targeted sialylated-fucosylated *N*-glycans investigated in the MRM approach.

**Figure 7:**
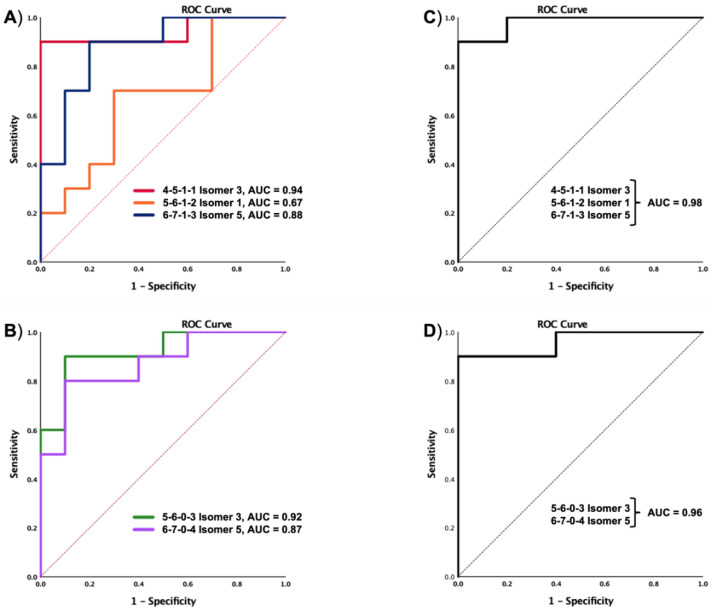
ROC curves of the *N*-glycans with statistically significant differences between control and MCI samples. Single ROC curves for the up regulated (**A**) and down regulated (**B**) *N*-glycans. Combined ROC curves for the up regulated (**C**) and down regulated (**D**) *N*-glycans. Glycan nomenclature: GlcNAc, Hex, Fuc, NeuAc (*N*-acetylglucosamine, Hexose, Fucose, *N*-acetylneuraminic acid).

The performance of the statistically significant glycans was evaluated using ROC curves, as shown in Figure 7. The results were as follows for each *N*-glycan: 0.94 for 4-5-1-1 Isomer 3, 0.67 for 5-6-1-2 Isomer 1, 0.87 for 6-7-1-3 Isomer 5, 0.92 for 5-6-0-3 Isomer 3, and 0.87 for 6-7-0-4 Isomer 5. Glycomics analysis has the advantage that related targets can be combined to gain sensitivity and specificity in the differentiation of two samples during a single analysis. Thus, we selectively combined the signals of the sialylated and the sialylated-fucosylated glycans using a binary logistic regression from SPSS software. For the sialylated glycans (5-6-0-3 Isomer 3 and 6-7-0-4 Isomer 5), the AUC value was enhanced to 0.96. For the sialylated-fucosylated glycans (4-5-1-1 Isomer 3, 4-5-1-2 Isomer 1, and 6-7-1-3 Isomer 5) the AUC value was enhanced to 0.98. In both cases, the AUC values were considerably larger than observed for single glycans.

### 3.4. Protein Analysis of the Depleted Serum Samples Extracted from Control and MCI Patients

Proteomics analysis of the low abundant proteins was performed to investigate the glycoprotein-source of the glycans described in the results. MaxQuant^®^ software was utilized for the identification and quantitation of the glycoproteins. The results showed a total of 120 glycoproteins, as shown in Supplementary Table S9. Among the identified glycoproteins, 15 showed statistical differences in abundance (*p* value < 0.05). The association of the identified glycoproteins with MCI and AD pathways was investigated using GeneOntology (GO) and ingenuity pathway analysis (IPA), Supplementary Figure S5A–C. The analysis showed the activation of the TP53 gene. For the canonical pathways, the “Acute Phase Response Signaling” pathway showed the highest significance, and the “Complement System” pathway was activated with a strong positive z-score. Additionally, the analysis showed that most of the detected glycoproteins were associated with the activation of the gene THOP1, a gene that has been widely studied because its expression changes during neurodegenerative diseases [43].

## 4. Discussion

The present study showed for the first time a glycome profile of serum low abundant glycoproteins extracted from the blood serum of control and MCI patients. Recently, MCI has been recognized as an important risk factor for developing more complicated neurodegenerative diseases such as AD [7]. Thus, the prediction of molecular changes in MCI, such as in the serum glycome, represent a minimally invasive tool for the diagnosis of early AD and other neurodegenerative diseases. Therefore, we focused this research on profiling the glycome of low abundant serum glycoproteins. A high percentage of the serum glycoproteome is composed of the *N*-glycans released from the high abundance glycoproteins, including IgG, antitrypsin, IgA, transferrin, haptoglobin, fibrinogen, alpha-2-macroglobulin, alpha1-acid glycoprotein, IgM, apolipoprotein AI, apolipoprotein AII, complement C3, and transthyretin [44]. The application of a depletion protocol (Section 2.3) eliminated the above-mentioned glycoproteins that would otherwise overlap the signal of the low abundance glycoproteins that may play an important role in the development of MCI and other MCI-derived neurodegenerative diseases. Additionally, it is well known that glycans have numerous isomeric forms. Among them, the most common isomeric changes are observed in the sialic acid linkage (α2,3 or α2,6) and modifications in the fucosylation (core or branch) [13]. Thus, we profiled the isomeric-glycome of the low abundant serum glycoproteins using two recent techniques developed in our research laboratory: the MGC-LC-MS that uses a column based on mesoporous graphitized carbon and the C18(50 cm)-LC-MS that used a long C18 column with a long chromatographic gradient. Both cases have proven to be excellent alternatives for the separation of isomeric glycans [30,31]. The results showed a large number of *N*-glycans when the C18(50 cm)-LC-MS strategy was applied to the samples, 80 in comparison to the 45 *N*-glycans identified using the MGC-LC-MS strategy. On the other hand, the MGC column showed better capabilities for the separation of isomers, with 39 base line resolved isomers against 29 poorly resolved isomeric glycans using the C18(50 cm) column. The results showed an extensive isomeric profile: 85 isomeric *N*-glycan structures from 45 *N*-glycan conformations with the MGC-LC-MS strategy and 109 isomeric *N*-glycan structures from 80 *N*-glycan conformations with the strategy C18(50 cm)-LC-MS. The analysis time is another important difference between the two analytical strategies. The MGC system used a gradient time of 70 min compared to a 220 min gradient used for the C18(50 cm) system. Despite the observed differences between the C18(50 cm) and the MGC strategies, the top 15 *N*-glycans were common to both approaches and represented 85% and 95% of the total glycan abundance detected, respectively (Figure 3). Among the top 15 *N*-glycans, the most representative types were: high mannose; bi-antennary mono-sialylated; di-sialylated with or without fucosylation (e.g., GlcNAc_4_,Hex_5_,Neu5Ac_2_); tri-antennary mono-, di-, and tri-sialylated with or without fucosylation (e.g., GlcNAc_5_,Hex_6_,Fuc, Neu5Ac); and tetra-antennary mono-, di-, tri-, and tetra-sialylated with or without fucosylation (e.g., GlcNAc_6_,Hex_7_,Fuc, Neu5Ac_4_). Among the common glycans, we found high mannose and other low abundant sialylated glycans. The specific glycan types observed in the MGC-LC-MS strategy were mainly glycan combinations of *N*-acetylglucosamine, mannose, galactose, and fucose, as shown in Figure 2A. The specific glycan types observed in the C18(50 cm)-LC-MS strategy were mainly galactosylated and large tri- and tetra-antennary complex glycans, as shown in Figure 2B.

Figure 4 showed the changes in the total glycome in the progression of healthy to MCI patients, regardless the isomeric changes. In the doughnut plots we can observe that both strategies, the MGC-LC-MS and C18(50 cm)-LC-MS follow the same trend of conversion from healthy to MCI samples. The main changes were observed for the sialylated glycans which increased 5.4% in abundance in the MCI samples. Conversely, the sialylated-fucosylated glycans decreased 4.6%. These specific structures included the “brain type” glycosylation characterized by antennary *N*-glycans with a significant amount of peripheral fucose (α1,3 linked to GlcNAc on the glycan antennae) resulting in a Lewis_x_ (Le_x_) or sialyl Lewis_x_ (sLe_x_) epitope, as well as the presence of *N*-acetylneuraminic acid (NeuAc) in α2,3 or α2,6 linkage [45]. Various glycomics studies of serum samples have reported important differences in the abundance of galactosylated-fucosylated glycans between healthy and AD patients, principally the biantennary di-galactosylated core-α1,6 fucosylated (NA2F) and the triantennary tri-galactosylated core-α1,6 fucosylated (NA3F) glycans [46]. The above-mentioned glycans were also observed in our strategies with low abundance and no statistical significance among the studied groups. These results suggest that previously reported glycan differences mainly come from the serum high abundant glycoproteins, such as IgG [47], portion of the sample that was depleted in this study.

The identification analysis completed on the MGC-LC-MS system prompted a large number of glycans and isomeric glycans with the ability to differentiate the sample groups (*p* value < 0.05), as shown in Table 2. Further, we validated these signals through target quantitation using an MRM approach with twelve of the identified *N*-glycans that also showed multiple isoforms, as shown in Supplementary Table S6. To investigate the ability of the validated data to provide insights into the separation between the control and MCI sample groups, we performed PCA, PLS-DA, and OPLS-DA chemometric analysis. A confidence interval of 95% was used to perform all the analyses. However, PCA is considered as a first-pass unsupervised tool to differentiated sample groups. It is highly recommended report cross-validation estimated scores plots for PLS and OPLS models [48,49]. Thus, the separation observed in all chemometric plots validated the groups’ differences when the identified *N*-isomeric glycans were used to visualize the cohort differences in Figure 5. For all the applied analysis, PCA, PLS-DA, and OPLS-DA the control samples 1 and 3 were observed separated from their cohort. Unfortunately, there is not sufficient clinical information to investigate sample differences. Therefore, a sample characteristic that came to our attention was the low *N*-glycan peak intensities observed for the above-mentioned samples with respect to the other analyzed samples. The sample size was normalized in accordance with protein concentration calculated using the Micro BCA^TM^ Protein Assay Kit (Thermo Scientific). The MGC-LC/MS-MRM analysis showed 46 isomeric *N*-glycans; the group signals were compared with the statistical *t*-test and the *p* values observed were corrected using the Benjamini-Hochberg procedure to reduce the false discovery rate (FDR), as shown in Table 2. After the statistical analyses were completed, the *N*-glycans 5-6-1-2 Isomer 1, 5-6-0-3 Isomer 3, 4-5-1-1 Isomer 3, 6-7-0-4 Isomer 5, and 6-7-1-3 Isomer 5 had the respective adjusted *p* values of 0.001, 0.009, 0.009, 0.02, and 0.02. The sialylated and sialylated-fucosylated isomeric *N*-glycans found with statistical significance correlated with the changes depicted for the glycome described in the glycan profile (Figure 2A,B). Thus, the sialylated-fucosylated *N*-glycans 4-5-1-1 Isomer 3, 5-6-1-2 Isomer 1, and 6-7-1-3 Isomer 5 decreased in abundance in the progression of control to MCI patients. Conversely, the sialylated *N*-glycans 5-6-0-3 Isomer 3 and 6-7-0-4 Isomer 5 increased in abundance in the progression of healthy to MCI patients. Additionally, we evaluated the diagnostic ability of the identified *N*-glycans using receiver operating characteristic (ROC) curves. Initially, the ROC/AUC curves were plotted for single glycan structures, as shown in Figure 7A,B. The single AUCs showed better results for two glycans: the sialylated-fucosylated glycan 4-5-1-1 Isomer 3 with an AUC of 0.94 (sensitivity of 90% and specificity of 40%) and the sialylated glycan 5-6-0-3 Isomer 3 with an AUC of 0.92 (sensitivity of 60% and specificity of 50%). Additionally, glycomics analyses have the advantage that different glycan signals observed in a single assay can be combined to gain sensitivity and specificity. Therefore, we investigated the possibility of using combined signals for two identified groups of statistically significant glycans (sialylated-fucosylated and sialylated) using a binary logistic regression to combine their signals. For the sialylated-fucosylated type, the combination of the three identified glycan signals increased the AUC to 0.98 with a sensitivity of 92% and specificity of 82% (Figure 7C). For the sialylated type, the combination of the two identified glycan signals increased the AUC to 0.96 with a sensitivity of 92% and specificity of 60% (Figure 7D). In both cases, we observed a substantial increase in the sensitivity and specificity of the signals.

There is substantial evidence that aberrant glycosylation in serum glycoproteins can be linked to neurological and neurodegenerative disorders [19,50]. In this study, we observed changes in the glycome expression of serum low abundant glycoproteins that may represent MCI glycofingerprints, and therefore insights for AD development. Glycosylation modulates the immune response [51]; the inflammatory response of monocytes, B cells, T cells, and also microglia are regulated by sialylated-type glycans [52,53]. Accordingly, our results indicated a significant increase of 5.5% in the abundance of the sialylated glycans in MCI (Figure 4), particularly the sialylated isomeric *N*-glycans 5-6-0-3 Isomer 3 and the 6-7-0-4 Isomer 5 with single AUC values of 0.92 and 0.87, respectively, and combined AUC of 0.96. In addition, we completed IPA analyses using the detected glycoproteins, as shown in Supplementary Table S9. The results showed the activation of the “Acute Phase Response Pathway” with a -log (*p* value) of 38 (Supplementary Figure S5B) as part of the immune response. Although the role of inflammation in neurodegenerative diseases such as AD is a controversial topic, many studies have pointed out the fundamental role that neuroinflammation plays in the progression of the neuropathological changes observed in AD [1]. Therefore, the upregulation of the isomeric glycans 5-6-0-3 (GlcNAc_5_,Hex_6_,Neu5Ac_3_ Isomer 3) and 6-7-0-4 (GlcNAc_6_,Hex_7_,Neu5Ac_4_ Isomer 5) observed in this study may represent an important avenue for future investigations, especially given the link between chronic inflammation and neurodegenerative diseases such as MCI and AD. The confirmatory mass spectra of these sialylated *N*-glycans is shown in Supplementary Figures S6 and S7. It’s also well known that sialylated glycans play an important role controlling neural transmission in the brain because of their effects on voltage gated sodium and potassium channels [54]. In this regard, it could be interesting for future work to investigate a correlation of the brain glycome changes with the modifications observed in the serum low abundant glycoproteins of MCI patients.

Additionally, three more glycans showed an important ability to differentiate between the sample cohorts: the 4-5-1-1 (GlcNAc_4_, Hex_5_, Fuc, Neu5Ac Isomer 3), the 5-6-1-2 (GlcNAc_5_,Hex_6_,Fuc, Neu5Ac_2_ Isomer 1), and the 6-7-1-3 (GlcNAc_6_, Hex_7_, Fuc, Neu5Ac_3_ Isomer 5), with respective single AUC values of 0.94, 0.67, and 0.88, and a combined AUC value of 0.96. These glycans were expressed with down regulation in the progression from healthy to MCI patients. The mass spectra of the structures indicate a branch fucosylation as can be observed in Supplementary Figures S8–S10. As previously mentioned, the fucosylation in the sialylated-fucosylated glycans implies important characteristics. For example, the formation of Lewis_x_ (Le_x_) or sialyl Lewis_x_ (sLe_x_) glycan epitopes have been recognized as brain type glycans in CSF because of their notorious changes in abundance in patients with AD [12,45,55]. Particularly in the brain, glycan branching modulates alterations that have been shown to affect myelination, neuronal excitability, the neuroinflammatory response, and promote neurodegeneration [56,57,58]. Thus, the types of glycans identified in our approach are of great interest, and future work should assess the identified glycans in large sample cohorts and particularly the differentiation of sialic acid or fucose linkages, which is a limitation of this approach. In this regard, the identification of the large number of isomeric glycans forms demands the development of easy and reliable identification techniques. Helm et al. proposed an interesting method that uses biosynthetic glycans with typical isomeric substitutions that can be used to normalize the isomeric glycan elution times [59].

Proteomics analysis of the low abundant glycoproteins was performed to investigate the glycoprotein-source of the glycans reported. Among the identified glycoproteins, 15 showed statistical differences in abundance (*p* value < 0.05), as shown in Supplementary Table S9. The association of the identified glycoproteins with MCI and AD pathways was investigated using IPA. The results showed the activation of the TP53 gene with a z-score of 2.2 by the glycoproteins APOE, VASN, MCAM, GPX3, GSN, and IGFBP3. The TP53 cascade showed the influence of these glycoproteins with the investigated disease, as shown in Supplementary Figure S5A: neurodegeneration of nerves (APOE); familial AD (APOE); finish type amyloidosis (APOE, GSN); brain damage (GPX3, GSN); autosomal dominant finish type (GSN); and autosomal dominant amyloidosis (APOE, GSN). For the canonical pathways the “Acute Phase Response Signaling” pathway showed the highest significance with a -log *p* value of 38, as shown in Supplementary Figure S5B. The IPA results also showed that most of the detected glycoproteins were associated with the activation of the gene THOP1 (Supplementary Figure S5C), a gene that has been widely studied due to its expression changes during neurodegenerative diseases [43,60]. Serum APOE is an *O-*glycosylated protein that has been linked with MCI and AD [61]; the statistical data of the proteomic results showed a clear differentiation between the tested cohorts with a *p*-value of 0.002, as shown in Supplementary Table S9. Therefore, the isolation and further *O-*glycomics analysis of serum APOE may bring insights of the progression from healthy to MCI states.

## Figures and Tables

**Table 1:** Patient clinical information.

Disease Diagnosis	Control ^1^	MCI ^2^
Number	10	10
Gender (M/F)	8/2	7/3
Age (mean ± SD)	73 ± 10	74 ± 8
Smoker (Yes/No)	3/7	4/6
Diabetes (Yes/No)	0/10	3/7

^1^ Control samples: Control 1, 2, 3, 4, 5, 6, 7, 8, 9, 10. ^2^ MCI samples: MCI 1, 2, 3, 4, 5, 6, 7, 8, 9, 10.

**Table 2:** Glycans with statistically significant changes in abundance between control and MCI samples. Glycan nomenclature: GlcNAc, Hex, Fuc, NeuAc (*N*-acetylglucosamine, Hexose, Fucose, *N*-acetylneuraminic acid).

Glycan Composition	*p* Value ^1^	Adjusted *p* Value ^2^
5-6-1-2 Isomer 1	0.00002	0.001
5-6-0-3 Isomer 3	0.0004	0.009
4-5-1-1 Isomer 3	0.0006	0.009
6-7-0-4 Isomer 5	0.001	0.02
6-7-1-3 Isomer 5	0.002	0.02
4-5-1-2 Isomer 5	0.008	0.06
4-5-1-1 Isomer 1	0.008	0.06
6-7-1-2	0.01	0.08
4-5-1-2 Isomer 4	0.01	0.07
4-5-2-2	0.02	0.08
6-7-1-3 Isomer 6	0.02	0.09
5-6-1-0	0.02	0.09
6-7-1-3 Isomer 4	0.03	0.09
5-6-1-2 Isomer 3	0.03	0.1
5-6-0-3 Isomer 4	0.03	0.09
5-6-1-2 Isomer 2	0.04	0.1

^1^*t*-test. ^2^ Benjamini-Hochberg correction.

## Data Availability

The mass spectrometry data generated during the study have been deposited to the Proteome Xchange Consortium via the PRIDE partner repository with the dataset identifier PXD036704 and Doi: 10.6019/PXD036704, username: reviewer_pxd036704@ebi.ac.uk, password: c3qWNZWA.

## Supplementary Materials

The following supporting information can be downloaded at: https://www.mdpi.com/article/10.3390/biom12111657/s1, Figure S1: EICs of the isomeric N-glycans identified; Figure S2: isomeric N-glycans observed with the MGC column; Figure S3: isomeric N-glycans observed with the C18(50 cm) column; Figure S4: heat map for the MRM MGC-LC-MS approach; Figure S5: ingenuity pathway analysis (IPA) of the identified glycoproteins; Figure S6: TIC and mass spectra of the N-glycan 5-6-0-3 Isomer 3; Figure S7: TIC and mass spectra of the N-glycan 6-7-0-4 Isomer 5; Figure S8: TIC and mass spectra of the N-glycan 4-5-1-1 Isomer 3; Figure S9: TIC and mass spectra of the N-glycan 5-6-1-2 Isomer 1; Figure S10: TIC and mass spectra of the N-glycan 6-7-1-3 Isomer 5; Table S1: clinical information; Table S2: MGC analysis, normalized abundance of all identified N-glycans digested from depleted low abundant serum glycoproteins of control patients; Table S3: MGC analysis, normalized abundance of all identified N-glycans digested from depleted low abundant serum glycoproteins of MCI patients; Table S4: C18(50 cm) analysis, normalized abundance of all identified N-glycans digested from depleted low abundant serum glycoproteins of control patients; Table S5: C18(50 cm) analysis, normalized abundance of all identified N-glycans digested from depleted low abundant serum glycoproteins of MCI patients; Table S6: MRM target list; Table S7: MGC MRM analysis, normalized abundance of targeted N-glycans digested from depleted low abundant serum glycoproteins of control patients; Table S8: MGC MRM analysis, normalized abundance of targeted N-glycans digested from depleted low abundant serum glycoproteins of MCI patients; Table S9: list of glycoproteins observed in the proteomics analysis of the low abundant serum glycoproteins from depleted serum samples of control and MCI patients.

## References

[1] Kinney J.W., Bemiller S.M., Murtishaw A.S., Leisgang A.M., Salazar A.M., Lamb B.T. (2018). Inflammation as a central mechanism in Alzheimer’s disease. Alzheimer’s Dement. Transl. Res. Clin. Interv..

[2] Ganguli M., Snitz B.E., Saxton J.A., Chang C.-C.H., Lee C.-W., Vander Bilt J., Hughes T.F., Loewenstein D.A., Unverzagt F.W., Petersen R.C. (2011). Outcomes of Mild Cognitive Impairment by Definition: A Population Study. Arch. Neurol..

[3] Farias S.T., Mungas D., Reed B.R., Harvey D., Cahn-Weiner D., Decarli C. (2006). MCI is associated with deficits in everyday functioning. Alzheimer Dis. Assoc. Disord..

[4] Dubois B., Albert M.L. (2004). Amnestic MCI or prodromal Alzheimer’s disease?. Lancet Neurol..

[5] Solfrizzi V., Panza F., Colacicco A.M., D’Introno A., Capurso C., Torres F., Grigoletto F., Maggi S., Del Parigi A., Reiman E.M. (2004). Vascular risk factors, incidence of MCI, and rates of progression to dementia. Neurology.

[6] Mallo S.C., Pereiro A.X., Campos-Magdaleno M., Nieto-Vieites A., Lojo-Seoane C., Facal D., Ismail Z., Juncos-Rabadán O. (2020). Neuropsychiatric symptoms in subjective cognitive complaints (SCC) and mild cognitive impairment (MCI): Detecting changes over time with the Mild Behavioral Impairment Checklist (MBI-C). Alzheimer’s Dement..

[7] Zhang D., Shen D. (2012). Predicting future clinical changes of MCI patients using longitudinal and multimodal biomarkers. PLoS ONE.

[8] Platero C., Tobar M.C. (2021). Predicting Alzheimer’s conversion in mild cognitive impairment patients using longitudinal neuroimaging and clinical markers. Brain Imaging Behav..

[9] Davatzikos C., Bhatt P., Shaw L.M., Batmanghelich K.N., Trojanowski J.Q. (2011). Prediction of MCI to AD conversion, via MRI, CSF biomarkers, and pattern classification. Neurobiol. Aging.

[10] Dennis J.W., Granovsky M., Warren C.E. (1999). Protein glycosylation in development and disease. Bioessays.

[11] Huang Y., Zhou S., Zhu J., Lubman D.M., Mechref Y. (2017). LC-MS/MS isomeric profiling of permethylated N-glycans derived from serum haptoglobin of hepatocellular carcinoma (HCC) and cirrhotic patients. Electrophoresis.

[12] Cho B.G., Veillon L., Mechref Y. (2019). N-Glycan Profile of Cerebrospinal Fluids from Alzheimer’s Disease Patients Using Liquid Chromatography with Mass Spectrometry. J. Proteome Res..

[13] Gutierrez Reyes C.D., Jiang P., Donohoo K., Atashi M., Mechref Y.S. (2021). Glycomics and glycoproteomics: Approaches to address isomeric separation of glycans and glycopeptides. J. Sep. Sci..

[14] Cho B.G., Gutierrez Reyes C.D., Mechref Y. (2021). N-Glycomics of Cerebrospinal Fluid: Method Comparison. Molecules.

[15] Palmigiano A., Barone R., Sturiale L., Sanfilippo C., Bua R.O., Romeo D.A., Messina A., Capuana M.L., Maci T., Le Pira F. (2016). CSF N-glycoproteomics for early diagnosis in Alzheimer’s disease. J. Proteom..

[16] Lundström S.L., Yang H., Lyutvinskiy Y., Rutishauser D., Herukka S.K., Soininen H., Zubarev R.A. (2014). Blood plasma IgG Fc glycans are significantly altered in Alzheimer’s disease and progressive mild cognitive impairment. J. Alzheimers Dis..

[17] Giau V.V., Bagyinszky E., An S.S.A. (2019). Potential Fluid Biomarkers for the Diagnosis of Mild Cognitive Impairment. Int. J. Mol. Sci..

[18] Schedin-Weiss S., Gaunitz S., Sui P., Chen Q., Haslam S.M., Blennow K., Winblad B., Dell A., Tjernberg L.O. (2020). Glycan biomarkers for Alzheimer disease correlate with T-tau and P-tau in cerebrospinal fluid in subjective cognitive impairment. FEBS J..

[19] Haukedal H., Freude K.K. (2021). Implications of Glycosylation in Alzheimer’s Disease. Front. Neurosci..

[20] Llop E., Ardá A., Zacco E., O’Flaherty R., García-Ayllón M.S., Aureli M., Frenkel-Pinter M., Reis C.A., Greiner-Tollersrud O.K., Cuchillo-Ibáñez I. (2022). Proceedings of workshop: “Neuroglycoproteins in health and disease”, INNOGLY cost action. Glycoconj. J..

[21] Peng W., Gutierrez Reyes C.D., Gautam S., Yu A., Cho B.G., Goli M., Donohoo K., Mondello S., Kobeissy F., Mechref Y. (2021). MS-based glycomics and glycoproteomics methods enabling isomeric characterization. Mass Spectrom. Rev..

[22] Akasaka-Manya K., Manya H., Sakurai Y., Wojczyk B.S., Kozutsumi Y., Saito Y., Taniguchi N., Murayama S., Spitalnik S.L., Endo T. (2009). Protective effect of N -glycan bisecting GlcNAc residues on β-amyloid production in Alzheimer’s disease. Glycobiology.

[23] Kizuka Y., Kitazume S., Taniguchi N. (2017). N-glycan and Alzheimer’s disease. Biochim. Biophys. Acta Gen. Subj..

[24] Bermingham M.L., Colombo M., McGurnaghan S.J., Blackbourn L.A.K., Vučković F., Pučić Baković M., Trbojević-Akmačić I., Lauc G., Agakov F., Agakova A.S. (2018). N-Glycan Profile and Kidney Disease in Type 1 Diabetes. Diabetes Care.

[25] Brownlee M. (1995). Advanced protein glycosylation in diabetes and aging. Annu. Rev. Med..

[26] Peng W., Goli M., Mirzaei P., Mechref Y. (2019). Revealing the Biological Attributes of N-Glycan Isomers in Breast Cancer Brain Metastasis Using Porous Graphitic Carbon (PGC) Liquid Chromatography-Tandem Mass Spectrometry (LC-MS/MS). J. Proteome Res..

[27] Stowell S.R., Ju T., Cummings R.D. (2015). Protein glycosylation in cancer. Annu. Rev. Pathol.

[28] Pinho S.S., Reis C.A. (2015). Glycosylation in cancer: Mechanisms and clinical implications. Nat. Rev. Cancer.

[29] Albert M.S., DeKosky S.T., Dickson D., Dubois B., Feldman H.H., Fox N.C., Gamst A., Holtzman D.M., Jagust W.J., Petersen R.C. (2011). The diagnosis of mild cognitive impairment due to Alzheimer’s disease: Recommendations from the National Institute on Aging-Alzheimer’s Association workgroups on diagnostic guidelines for Alzheimer’s disease. Alzheimers Dement..

[30] Gautam S., Banazadeh A., Cho B.G., Goli M., Zhong J., Mechref Y. (2021). Mesoporous Graphitized Carbon Column for Efficient Isomeric Separation of Permethylated Glycans. Anal. Chem..

[31] Wang J., Dong X., Yu A., Huang Y., Peng W., Mechref Y. (2022). Isomeric separation of permethylated glycans by extra-long reversed-phase liquid chromatography (RPLC)-MS/MS. Analyst.

[32] Donohoo K.B., Wang J., Goli M., Yu A., Peng W., Hakim M.A., Mechref Y. (2021). Advances in mass spectrometry-based glycomics-An update covering the period 2017–2021. Electrophoresis.

[33] Dong X., Huang Y., Cho B.G., Zhong J., Gautam S., Peng W., Williamson S.D., Banazadeh A., Torres-Ulloa K.Y., Mechref Y. (2018). Advances in mass spectrometry-based glycomics. Electrophoresis.

[34] Tsai T.H., Wang M., Di Poto C., Hu Y., Zhou S., Zhao Y., Varghese R.S., Luo Y., Tadesse M.G., Ziada D.H. (2014). LC-MS profiling of N-Glycans derived from human serum samples for biomarker discovery in hepatocellular carcinoma. J. Proteome Res..

[35] Meng Z., Veenstra T.D., Issaq H.J., Veenstra T.D. (2013). Chapter 26—Mass Spectrometry–Based Approach for Protein Biomarker Verification. Proteomic and Metabolomic Approaches to Biomarker Discovery.

[36] Parker C.E., Borchers C.H. (2014). Mass spectrometry based biomarker discovery, verification, and validation--quality assurance and control of protein biomarker assays. Mol. Oncol..

[37] Zhou S., Hu Y., DeSantos-Garcia J.L., Mechref Y. (2015). Quantitation of permethylated N-glycans through multiple-reaction monitoring (MRM) LC-MS/MS. J. Am. Soc. Mass Spectrom..

[38] (2013). Method of the Year 2012. Nat. Methods.

[39] Tu C., Rudnick P.A., Martinez M.Y., Cheek K.L., Stein S.E., Slebos R.J.C., Liebler D.C. (2010). Depletion of abundant plasma proteins and limitations of plasma proteomics. J. Proteome Res..

[40] Zhou S., Hu Y., Mechref Y. (2016). High-temperature LC-MS/MS of permethylated glycans derived from glycoproteins. Electrophoresis.

[41] Kurz S., Sheikh M.O., Lu S., Wells L., Tiemeyer M. (2021). Separation and Identification of Permethylated Glycan Isomers by Reversed Phase NanoLC-NSI-MSn. Mol. Cell. Proteom..

[42] Shajahan A., Supekar N., Heiss C., Azadi P. (2019). High-Throughput Automated Micro-permethylation for Glycan Structure Analysis. Anal. Chem..

[43] Ubina T., Magallanes M., Srivastava S., Warden C., Yee J.-K., Salvaterra P.M. (2018). A human embryonic stem cell model of Aβ-dependent chronic progressive neurodegeneration. bioRxiv.

[44] Klein A., Carre Y., Louvet A., Michalski J.-C., Morelle W. (2010). Immunoglobulins are the major glycoproteins involved in the modifications of total serum N-glycome in cirrhotic patients. PROTEOMICS–Clin. Appl..

[45] Barone R., Sturiale L., Palmigiano A., Zappia M., Garozzo D. (2012). Glycomics of pediatric and adulthood diseases of the central nervous system. J. Proteom..

[46] Chen C.C., Engelborghs S., Dewaele S., Le Bastard N., Martin J.J., Vanhooren V., Libert C., De Deyn P.P. (2010). Altered serum glycomics in Alzheimer disease: A potential blood biomarker?. Rejuvenation Res..

[47] Parekh R., Roitt I., Isenberg D., Dwek R., Rademacher T. (1988). Age-related galactosylation of the N-linked oligosaccharides of human serum IgG. J. Exp. Med..

[48] Worley B., Powers R. (2016). PCA as a practical indicator of OPLS-DA model reliability. Curr. Metab..

[49] Worley B., Powers R. (2014). MVAPACK: A complete data handling package for NMR metabolomics. ACS Chem. Biol..

[50] Mondello S., Sandner V., Goli M., Czeiter E., Amrein K., Kochanek P.M., Gautam S., Cho B.G., Morgan R., Nehme A. (2022). Exploring serum glycome patterns after moderate to severe traumatic brain injury: A prospective pilot study. EClinicalMedicine.

[51] van Kooyk Y., Rabinovich G.A. (2008). Protein-glycan interactions in the control of innate and adaptive immune responses. Nat. Immunol..

[52] Crocker P.R., Paulson J.C., Varki A. (2007). Siglecs and their roles in the immune system. Nat. Rev. Immunol..

[53] Blixt O., Collins B.E., van den Nieuwenhof I.M., Crocker P.R., Paulson J.C. (2003). Sialoside Specificity of the Siglec Family Assessed Using Novel Multivalent Probes: Identification of potent inhibitors of myelin-associated glycoprotein *. J. Biol. Chem..

[54] Scott H., Panin V.M. (2014). The role of protein N-glycosylation in neural transmission. Glycobiology.

[55] Hoffmann A., Nimtz M., Wurster U., Conradt H.S. (1994). Carbohydrate Structures of β-Trace Protein from Human Cerebrospinal Fluid: Evidence for “Brain-Type”N-Glycosylation. J. Neurochem..

[56] Lee S.U., Grigorian A., Pawling J., Chen I.J., Gao G., Mozaffar T., McKerlie C., Demetriou M. (2007). N-glycan processing deficiency promotes spontaneous inflammatory demyelination and neurodegeneration. J. Biol. Chem..

[57] Mkhikian H., Grigorian A., Li C.F., Chen H.L., Newton B., Zhou R.W., Beeton C., Torossian S., Tatarian G.G., Lee S.U. (2011). Genetics and the environment converge to dysregulate N-glycosylation in multiple sclerosis. Nat. Commun..

[58] Kleene R., Schachner M. (2004). Glycans and neural cell interactions. Nat. Rev. Neurosci..

[59] Helm J., Hirtler L., Altmann F. (2022). Towards Mapping of the Human Brain N-Glycome with Standardized Graphitic Carbon Chromatography. Biomolecules.

[60] Pollio G., Hoozemans J.J.M., Andersen C.A., Roncarati R., Rosi M.C., van Haastert E.S., Seredenina T., Diamanti D., Gotta S., Fiorentini A. (2008). Increased expression of the oligopeptidase THOP1 is a neuroprotective response to Aβ toxicity. Neurobiol. Dis..

[61] Sebastiani P., Monti S., Morris M., Gurinovich A., Toshiko T., Andersen S.L., Sweigart B., Ferrucci L., Jennings L.L., Glass D.J. (2019). A serum protein signature of APOE genotypes in centenarians. Aging Cell.

