# Supplementary material for: LC-MS/MS Isomeric Profiling of N-Glycans Derived from Low-Abundant Serum Glycoproteins in Mild Cognitive Impairment Patients
Source: Biomolecules. 2022 Nov 8;12(11):1657. doi: 10.3390/biom12111657 (PMC9687829; doi:10.3390/biom12111657)
Supplement: Supplementary file 1 [file biomolecules-12-01657-s001.zip › biomolecules-2010562-supplementary.pdf]

[Supplementary data]

LC-MS/MS Isomeric Profiling of *N*-glycans Derived from Low Abundant Serum Glycoproteins in Mild Cognitive Impairment Patients

Cristian D. Gutierrez Reyes[1], Md Abdul Hakim[1], Atashi Mojgan[1], Mona Goli[1], Sakshi Gautman[1], Junyao Wang[1], Andrew I. Bennett[1], Jianhui Zhu[2], David M. Lubman[2] and Yehia Mechref[1*]

1. Department of Chemistry and Biochemistry, Texas Tech University, Lubbock, TX
2. Department of Surgery, The University of Michigan, Ann Arbor, MI 48109

*Corresponding author

Department of Chemistry and Biochemistry

Texas Tech University

Lubbock, TX 79409-1061

Email: yehia.mechref@ttu.edu

Tel: 806-742-3059

Fax: 806-742-1289

Table of Contents:

Supplementary Figures:

Supplementary Figure S1. EICs of the isomeric *N*-glycans identified using the **A**) MGC-LC-MS system and the **B**) C18(50cm)-LC-MS system.

Supplementary Figure S2. Isomeric *N*-glycans observed with the **MGC** column. Glycan nomenclature: GlcNAc, Hex, Fuc, NeuAc (*N*-acetylglucosamine, Hexose, Fucose, *N*-acetylneuraminic acid).

Supplementary Figure S3. Isomeric *N*-glycans observed with the **C18(50cm)** column. Glycan nomenclature: GlcNAc, Hex, Fuc, NeuAc (*N*-acetylglucosamine, Hexose, Fucose, *N*-acetylneuraminic acid).

Supplementary Figure S4. Heat map for the MRM MGC-LC-MS approach.

Supplementary Figure S5. Ingenuity Pathway Analysis (IPA) of the identified glycoproteins. A) Activation of the TP53 gene, B) canonical pathway identification, and C) activation of the THOP1 gene.

Supplementary Figure S6. TIC and mass spectra of the N-glycan 5-6-0-3 Isomer 3.

Supplementary Figure S7. TIC and mass spectra of the N-glycan 6-7-0-4 Isomer 5.

Supplementary Figure S8. TIC and mass spectra of the *N*-glycan 4-5-1-1 Isomer 3.

Supplementary Figure S9. TIC and mass spectra of the *N*-glycan 5-6-1-2 Isomer 1.

Supplementary Figure S10. TIC and mass spectra of the *N*-glycan 6-7-1-3 Isomer 5.

Supplementary Tables:

Supplementary Table S1. Clinical information.

Supplementary Table S2. MGC analysis, normalized abundance of all identified *N*-glycans digested from depleted low abundant serum glycoproteins of **control** patients. GlcNAc, Hex, Fuc, NeuAc (*N*-acetylglucosamine, Hexose, Fucose, *N*-acetylneuraminic acid), and Iso = isomer.

Supplementary Table S3. MGC analysis, normalized abundance of all identified *N*-glycans digested from depleted low abundant serum glycoproteins of **MCI** patients. GlcNAc, Hex, Fuc, NeuAc (*N*-acetylglucosamine, Hexose, Fucose, *N*-acetylneuraminic acid), and Iso = isomer.

Supplementary Table S4. C18(50cm) analysis, normalized abundance of all identified *N*-glycans digested from depleted low abundant serum glycoproteins of **control** patients. GlcNAc, Hex, Fuc, NeuAc (*N*-acetylglucosamine, Hexose, Fucose, *N*-acetylneuraminic acid), and Iso = isomer.

Supplementary Table S5. C18(50cm) analysis, normalized abundance of all identified *N*-glycans digested from depleted low abundant serum glycoproteins of **MCI** patients. GlcNAc, Hex, Fuc, NeuAc (*N*-acetylglucosamine, Hexose, Fucose, *N*-acetylneuraminic acid), and Iso = isomer.

Supplementary Table S6. MRM target list. GlcNAc, Hex, Fuc, NeuAc (*N*-acetylglucosamine, Hexose, Fucose, *N*-acetylneuraminic acid), and Iso = isomer.

Supplementary Table S7. MGC **MRM** analysis, normalized abundance of targeted *N*-glycans digested from depleted low abundant serum glycoproteins of **control** patients. GlcNAc, Hex, Fuc, NeuAc (*N*-acetylglucosamine, Hexose, Fucose, *N*-acetylneuraminic acid), and Iso = isomer.

Supplementary Table S8. MGC **MRM** analysis, normalized abundance of targeted *N*-glycans digested from depleted low abundant serum glycoproteins of **MCI** patients. GlcNAc, Hex, Fuc, NeuAc (*N*-acetylglucosamine, Hexose, Fucose, *N*-acetylneuraminic acid), and Iso = isomer.

Supplementary Table S9. List of glycoproteins observed in the proteomic analysis of the low abundant serum glycoproteins from depleted serum samples of control and MCI patients.

Supplementary Figure S1. EICs of the isomeric *N*-glycans identified using the **A**) MGC-LC-MS system and the **B**) C18(50cm)-LC-MS system.

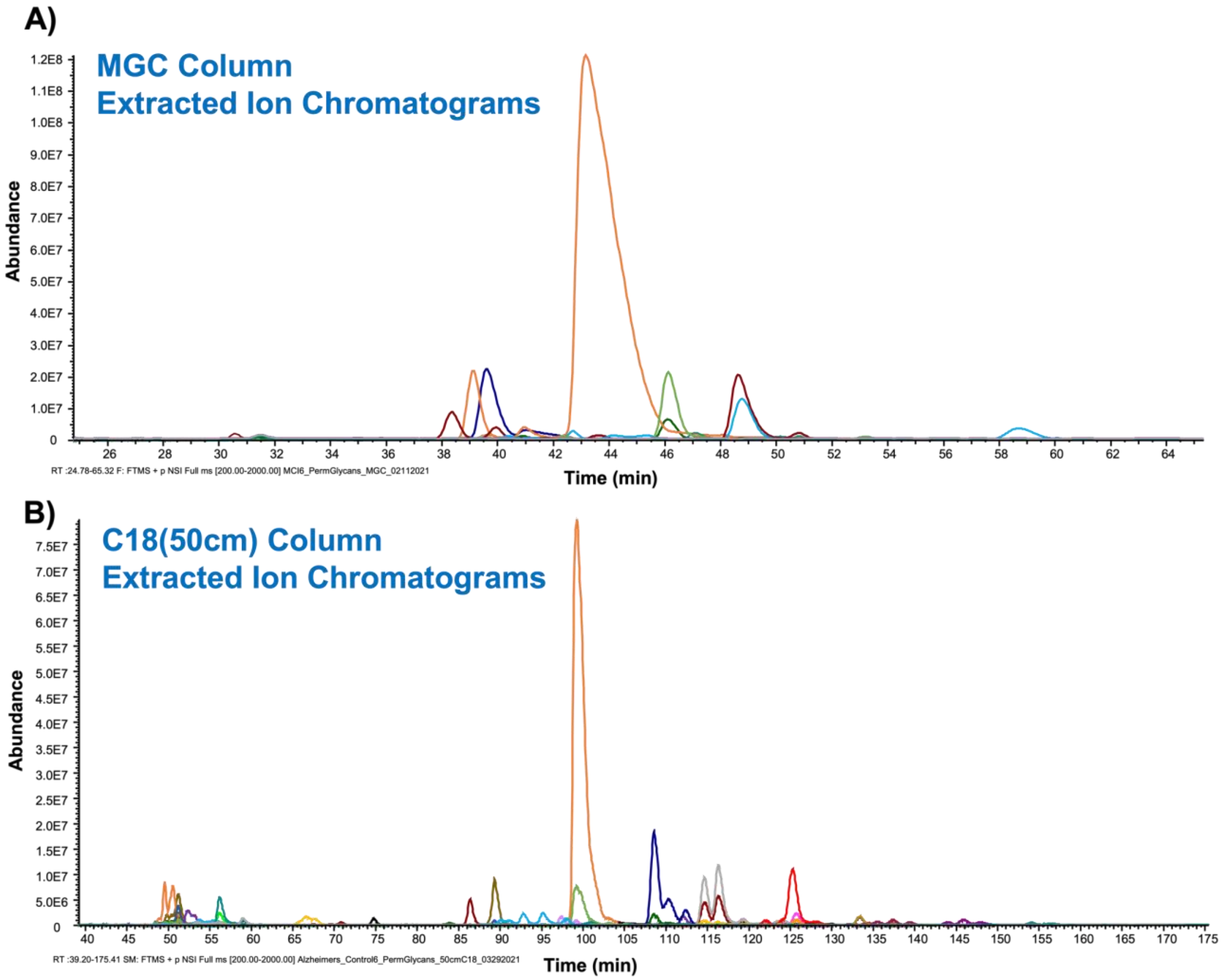

Supplementary Figure S2. Isomeric *N*-glycans observed with the **MGC** column. Glycan nomenclature: GlcNAc, Hex, Fuc, NeuAc (*N*-acetylglucosamine, Hexose, Fucose, *N*-acetylneuraminic acid).

Glycan 2-8-0-0

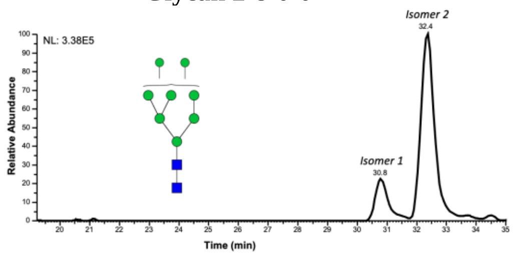

Glycan 4-5-1-1

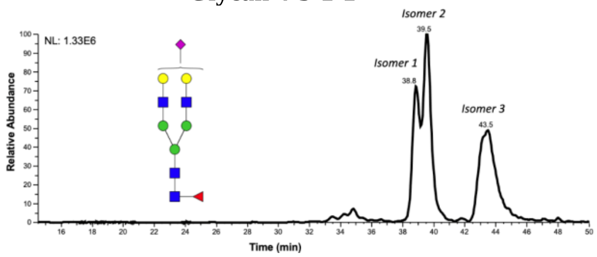

Glycan 4-5-0-2

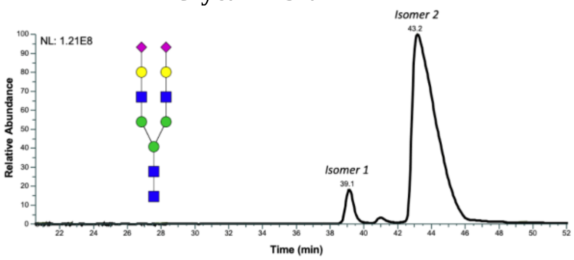

Glycan 5-6-0-1

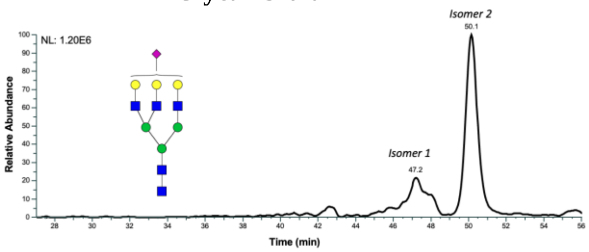

Glycan 5-8-0-0

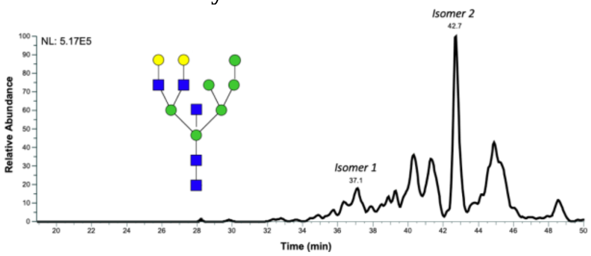

Glycan 4-5-1-2

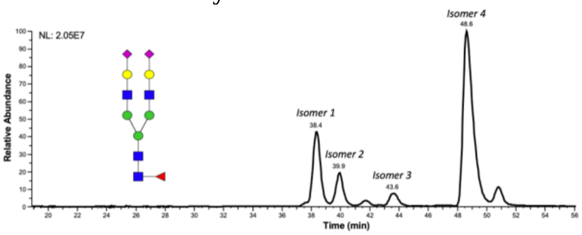

Glycan 4-6-0-2

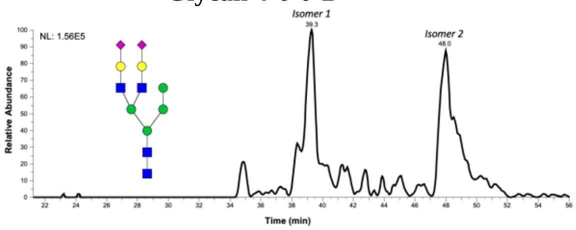

Glycan 5-6-1-1

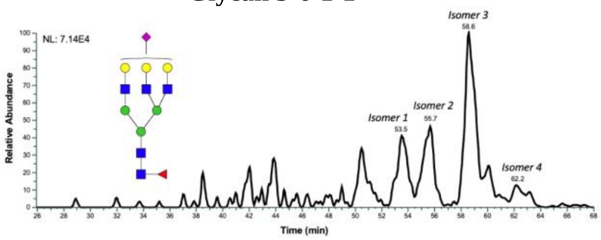

Glycan 5-6-0-2

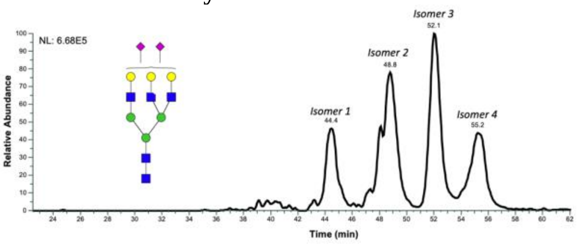

Glycan 8-6-0-0

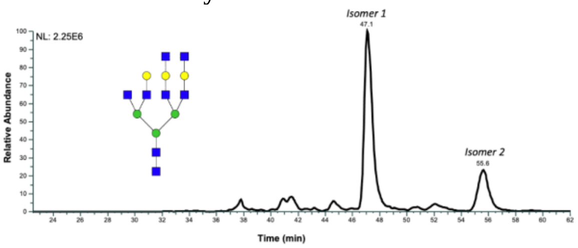

Supplementary Figure S2. Continued…

Glycan 5-6-1-2

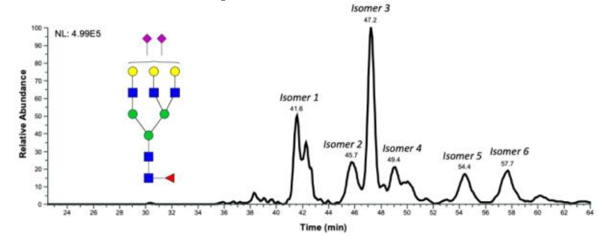

Glycan 5-6-0-3

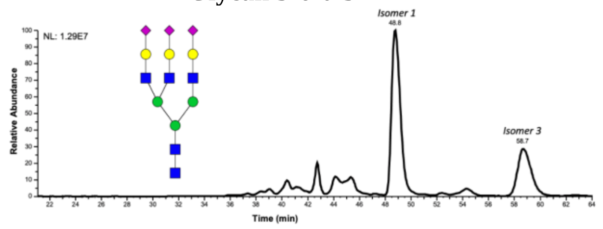

Glycan 5-6-1-3

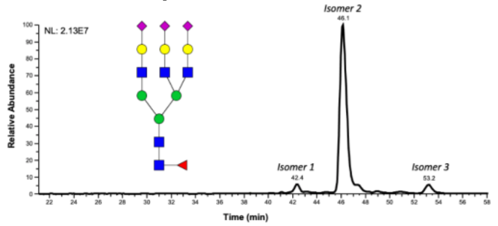

Glycan 5-8-3-1

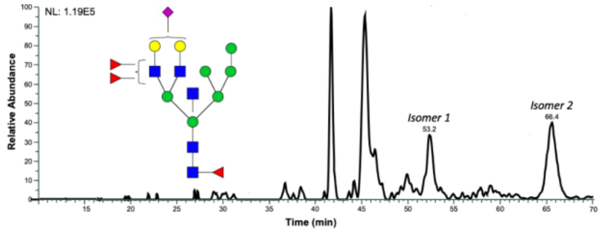

Glycan 5-6-2-3

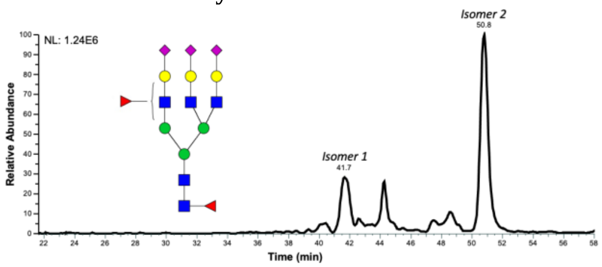

Glycan 6-7-0-3

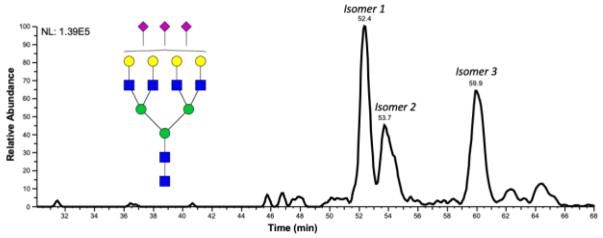

Glycan 6-7-1-3

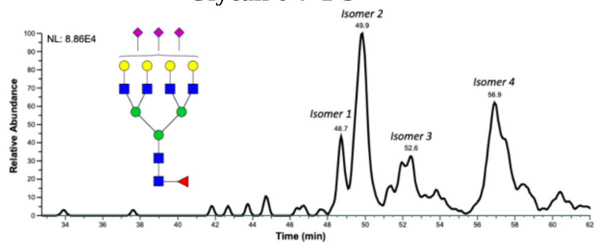

Glycan 6-7-0-4

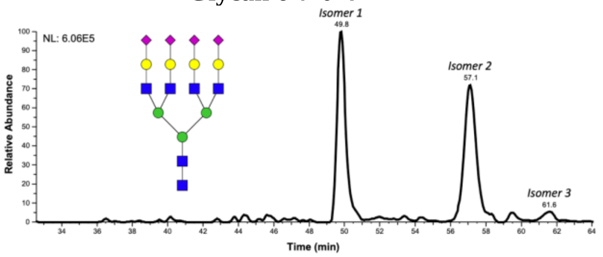

Glycan 6-7-1-4

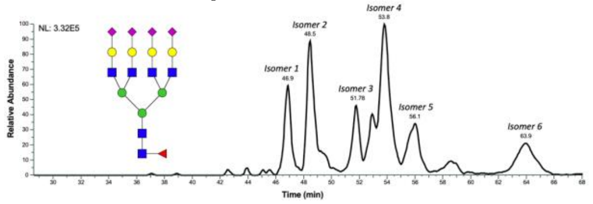

Glycan 6-7-2-4

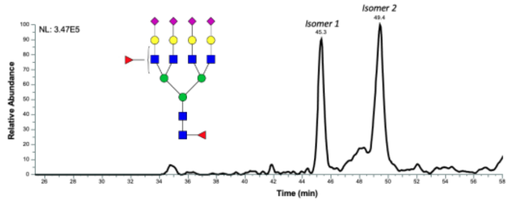

Supplementary Figure S3. Isomeric *N*-glycans observed with the **C18(50cm)** column. Glycan nomenclature: GlcNAc, Hex, Fuc, NeuAc (*N*-acetylglucosamine, Hexose, Fucose, *N*-acetylneuraminic acid).

Glycan 4-4-2-1

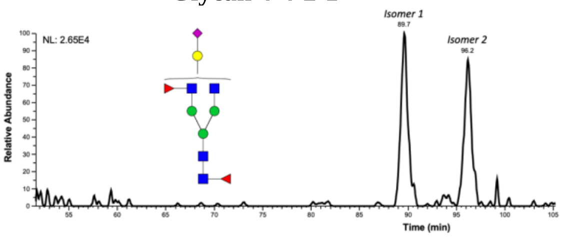

Glycan 4-5-1-0

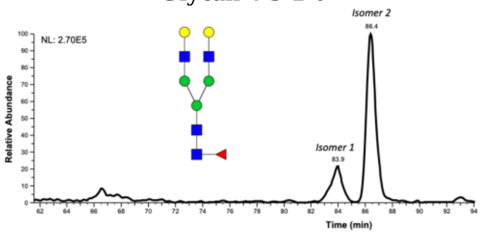

Glycan 4-5-1-1

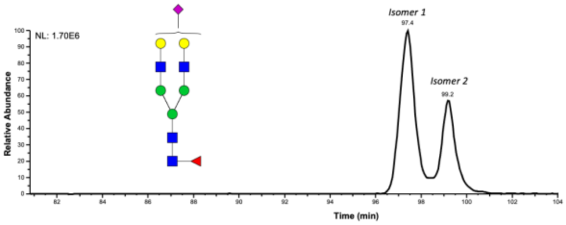

Glycan 4-5-1-2

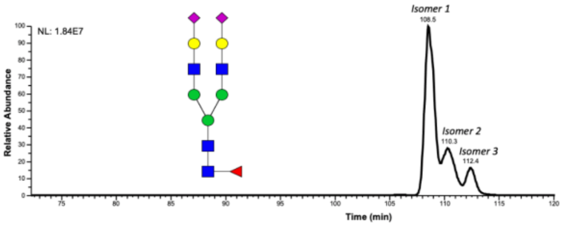

Glycan 4-6-2-0

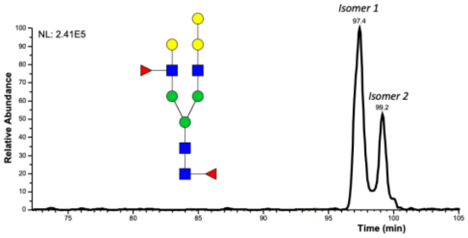

Glycan 5-5-0-2

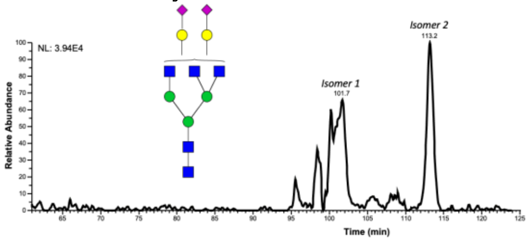

Glycan 5-6-0-1

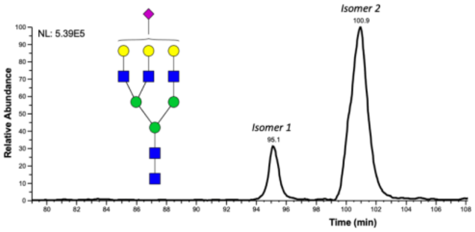

Glycan 5-6-0-3

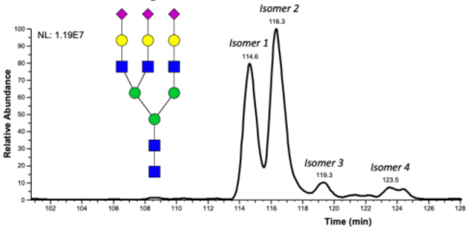

Glycan 5-6-0-4

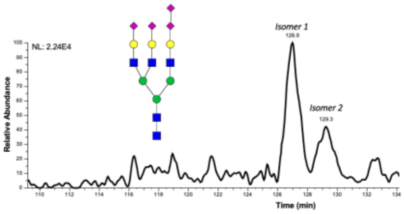

Glycan 5-6-1-2

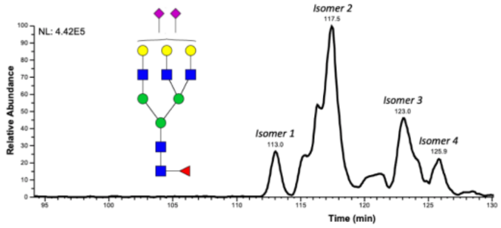

Supplementary Figure S3. Continued…

Glycan 5-6-1-3

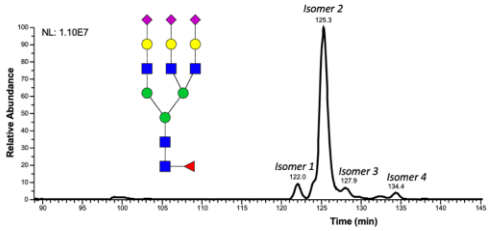

Glycan 5-6-2-3

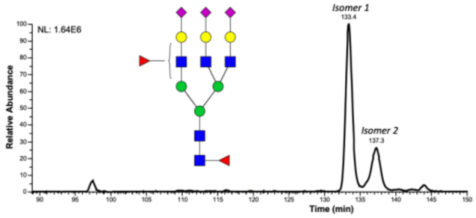

Glycan 5-7-1-2

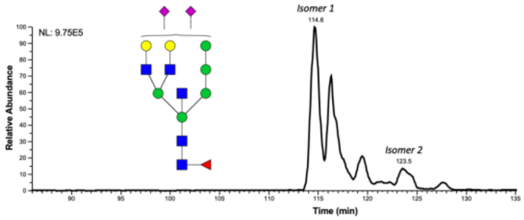

Glycan 6-7-0-4

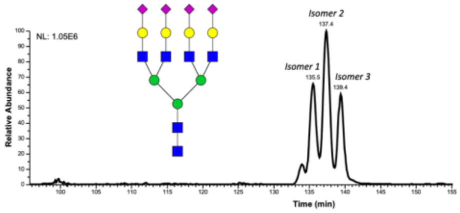

Glycan 6-7-1-2

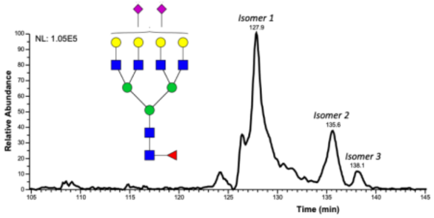

Glycan 6-7-1-4

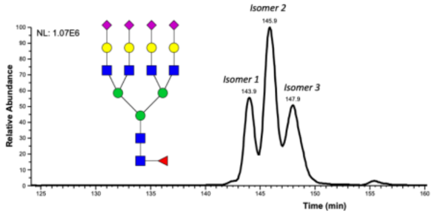

Glycan 6-7-2-4

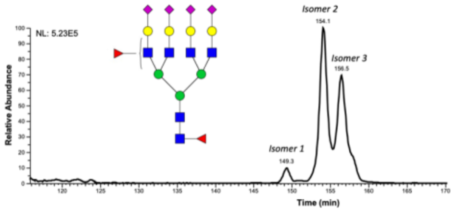

Glycan 7-8-2-4

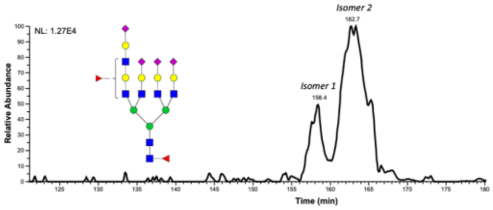

Glycan 8-6-0-1

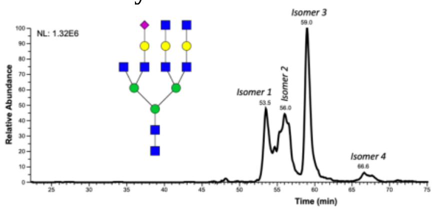

Supplementary Figure S4. Heat map for the MRM MGC-LC-MS approach.

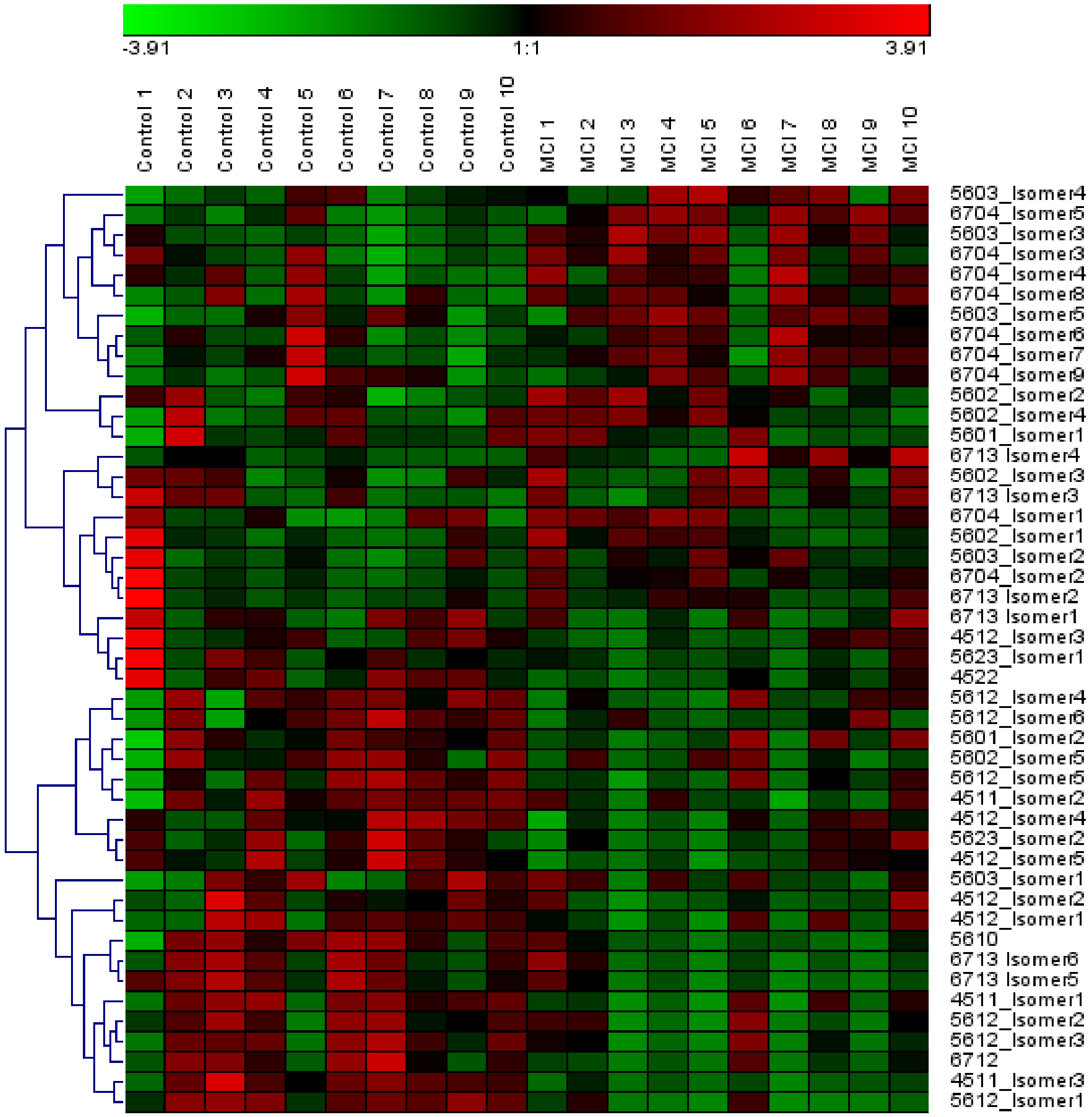

Supplementary Figure S5. Ingenuity Pathway Analysis (IPA) of the identified glycoproteins. A) Activation of the TP53 gene, B) canonical pathway identification, and C) activation of the THOP1 gene.

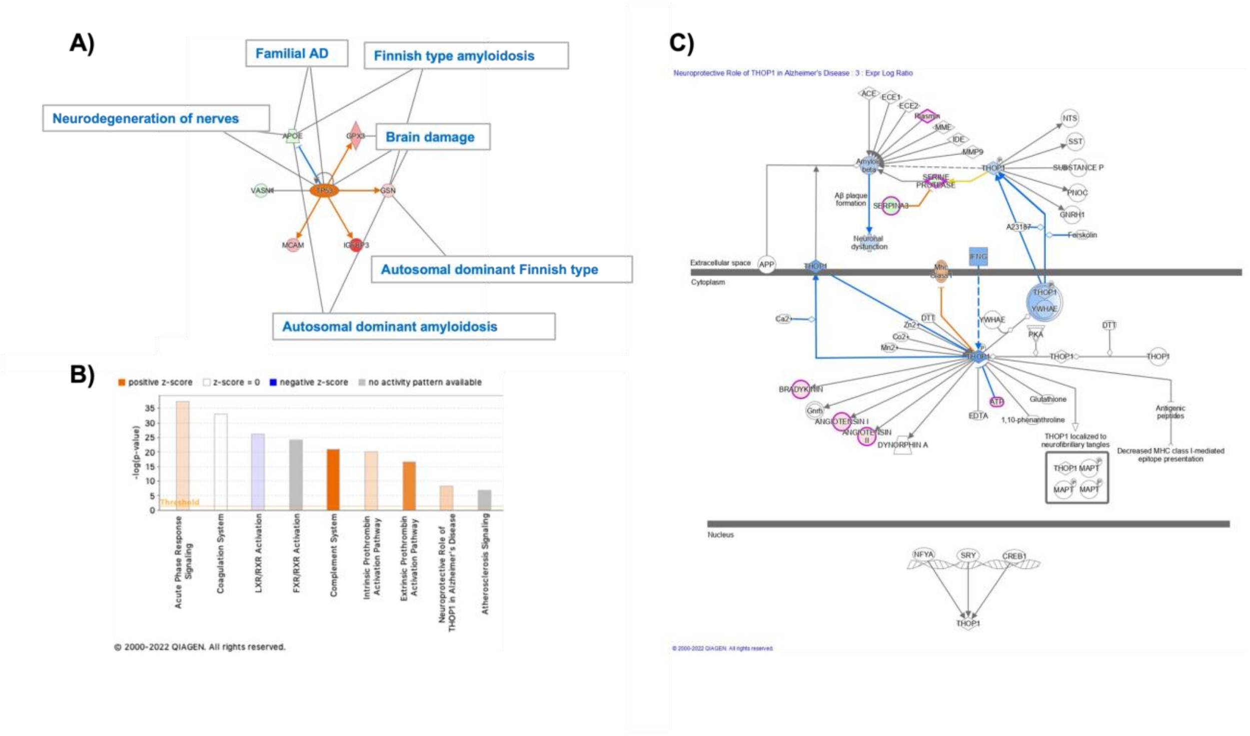

Supplementary Figure S6

Supplementary Figure S6. TIC and mass spectra of the *N*-glycan 5-6-0-3 Isomer 3.

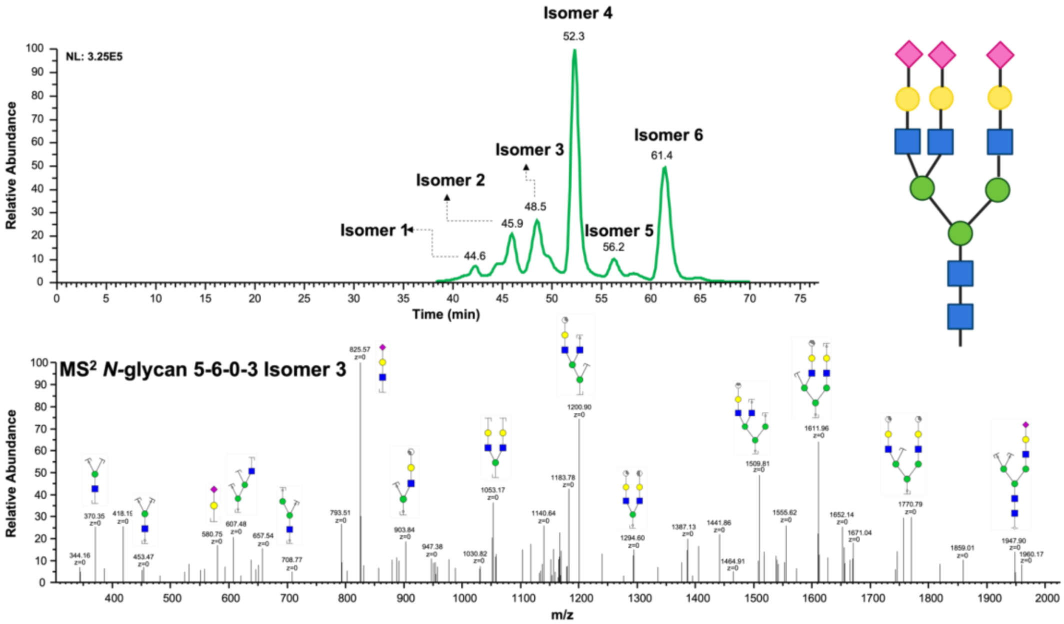

Supplementary Figure S7

Supplementary Figure S7. TIC and mass spectra of the *N*-glycan 6-7-0-4 Isomer 5.

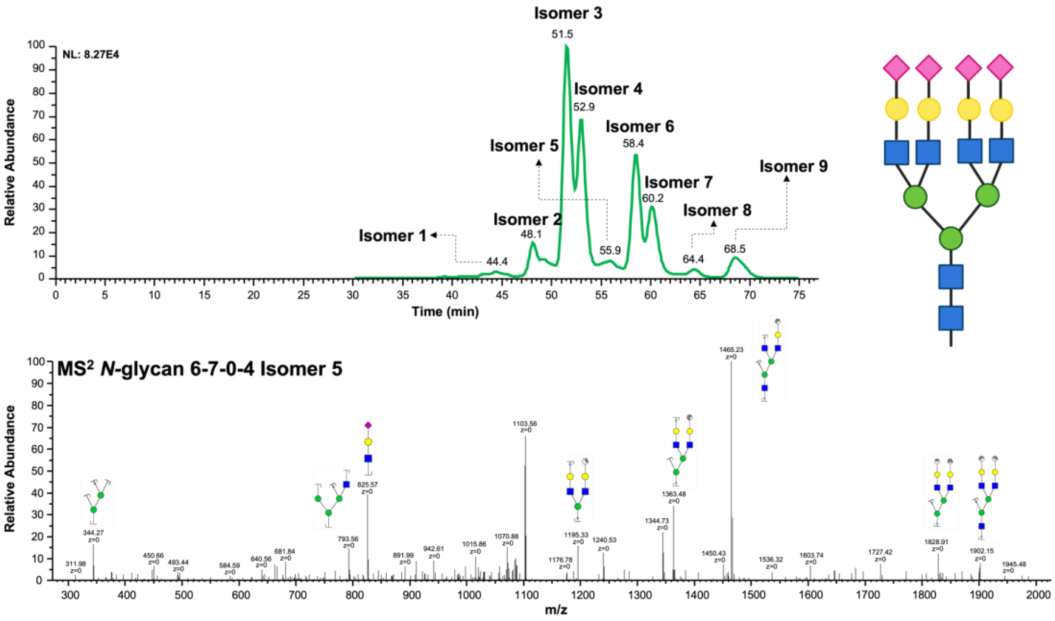

Supplementary Figure S8. TIC and mass spectra of the *N*-glycan 4-5-1-1 Isomer 3.

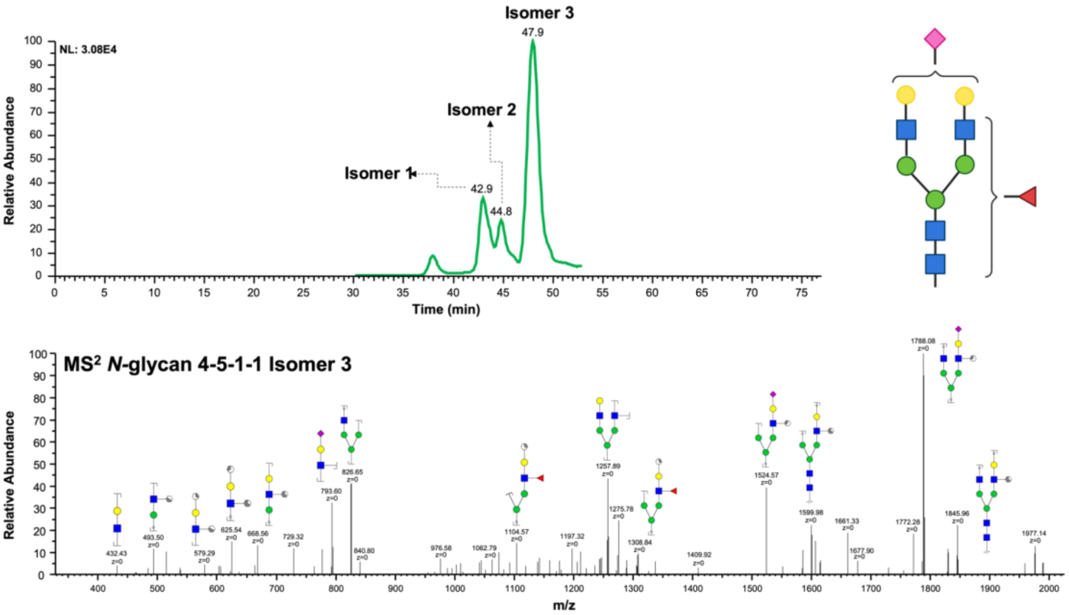

Supplementary Figure S9. TIC and mass spectra of the *N*-glycan 5-6-1-2 Isomer 1.

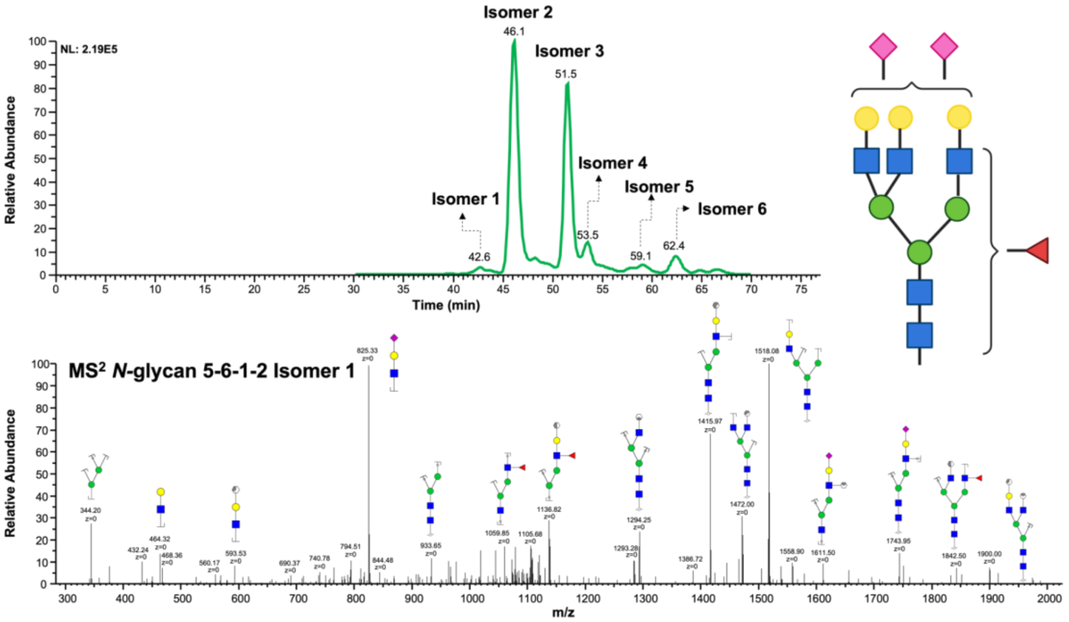

Supplementary Figure S10. TIC and mass spectra of the *N*-glycan 6-7-1-3 Isomer 5.

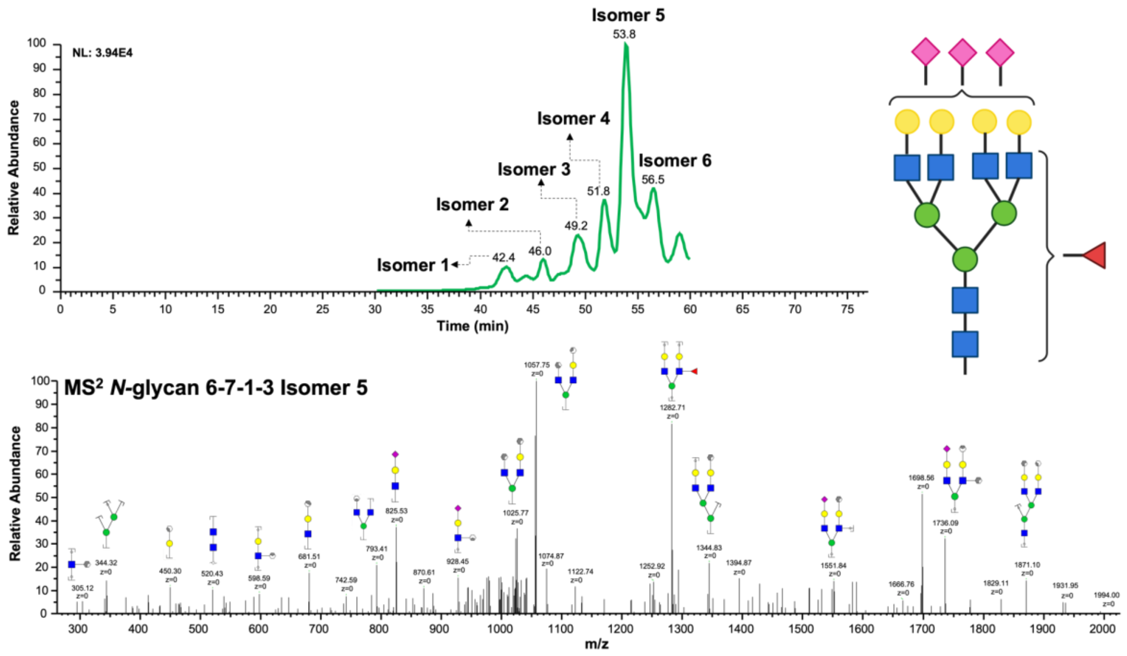

Supplementary Table S1. Clinical information.

Sample / Diagnosis	Age	Sex	Other observations
Control 1	75	Male	Former smoker
Control 2	64	Female	Non-smoker (less than 100 times in life)
Control 3	69	Male	Smokes a pipe
Control 4	85	Male	Former smoker, quit more than 30 days ago
Control 5	65	Male	Non-smoker (less than 100 times in life)
Control 6	87	Male	Non-smoker (less than 100 times in life)
Control 7	67	Male	Non-smoker (less than 100 times in life)
Control 8	58	Male	Non-smoker (less than 100 times in life)
Control 9	82	Female	Non-smoker (less than 100 times in life)
Control 10	80	Male	Non-smoker (less than 100 times in life)
MCI 1	61	Male	Former smoker, quit more than 30 days ago and Diabetes
MCI 2	78	Female	Former smoker, quit more than 30 days ago.
MCI 3	65	Female	Non-smoker (less than 100 times in life)
MCI 4	67	Male	Non-smoker (less than 100 times in life) and Diabetes
MCI 5	77	Male	Non-smoker
MCI 6	69	Male	Non-smoker (less than 100 times in life)
MCI 7	84	Male	Non-smoker (less than 100 times in life)
MCI 8	86	Male	Non-smoker (less than 100 times in life) and Diabetes
MCI 9	78	Male	Former smoker, quit more than 1 year ago
MCI 10	77	Female	Former smoker, quit more than 30 days ago

Supplementary Table S2. MGC analysis, normalized abundance of all identified *N*-glycans digested from depleted low abundant serum glycoproteins of **control** patients. GlcNAc, Hex, Fuc, NeuAc (*N*-acetylglucosamine, Hexose, Fucose, *N*-acetylneuraminic acid), and Iso = isomer.

Glycan	Control 1	Control 2	Control 3	Control 4	Control 5	Control 6	Control 7	Control 8	Control 9	Control 10
**2-5-0-0**	0.3425	0.3044	0.5798	0.4057	0.3820	0.3380	0.2942	0.2488	0.3247	0.5128
**2-6-0-0**	0.7509	1.3072	0.4924	0.9121	0.7294	1.0723	0.7075	0.7359	0.8162	1.1109
**3-5-0-0**	0.0110	0.0089	0.1023	0.0086	0.0064	0.0038	0.0342	0.0081	0.0089	0.0114
**2-7-0-0**	0.0948	0.2282	0.0763	0.0990	0.0917	0.0856	0.1428	0.1139	0.0931	0.1123
**3-6-0-0**	0.0174	0.0153	0.0503	0.0164	0.0172	0.0109	0.0218	0.0169	0.0202	0.0192
**6-3-0-0**	0.0084	0.0254	0.0313	0.0294	0.0017	0.0391	0.0051	0.0214	0.0181	0.0216
**3-5-0-1**	0.0495	0.0806	0.1212	0.0953	0.0610	0.0718	0.0871	0.0999	0.0632	0.1066
**2-8-0-0 Iso 1**	0.0220	0.0265	0.0227	0.0216	0.0318	0.0314	0.0175	0.0250	0.0280	0.0302
**2-8-0-0 Iso 2**	0.1312	0.2354	0.0977	0.1400	0.1240	0.1852	0.1138	0.1409	0.1493	0.1996
**4-4-0-1**	0.1131	0.1392	0.5508	0.0817	0.0485	0.0760	0.0384	0.0597	0.1084	0.1042
**4-5-1-0**	0.0518	0.0489	0.4286	0.0319	0.0238	0.0345	0.0283	0.0242	0.0464	0.0411
**2-9-0-0**	0.3816	0.6271	0.2319	0.4306	0.4663	0.5592	0.3694	0.4201	0.4090	0.5464
**4-5-0-1**	7.2440	7.0037	7.2038	5.1859	4.3125	3.9517	3.3854	4.7242	6.6814	5.0279
**4-6-1-0**	0.6638	0.8245	0.8570	0.5977	0.2333	0.3918	0.1518	0.4815	0.5123	0.5544
**5-5-1-0**	0.0367	0.0248	0.2888	0.0198	0.0291	0.0214	0.0370	0.0200	0.0360	0.0326
**2-10-0-0**	0.0064	0.0070	0.0006	0.0050	0.0067	0.0070	0.0046	0.0067	0.0052	0.0065
**4-5-1-1 Iso 1**	0.5186	0.5142	0.6062	0.5207	0.3213	0.4804	0.6593	0.4245	0.5409	0.5724
**4-5-1-1 Iso 2**	0.4441	0.4245	1.9288	0.2856	0.3889	0.3952	0.6404	0.3440	0.4274	0.3479
**4-6-0-1**	0.0637	0.0483	0.0663	0.0540	0.0609	0.0490	0.0505	0.0553	0.0639	0.0505
**5-5-0-1**	0.0178	0.0024	0.1052	0.0274	0.0099	0.0607	0.0017	0.0018	0.0204	0.0248
**5-6-1-0**	0.0111	0.0197	0.0607	0.0154	0.0027	0.0266	0.0020	0.0015	0.0104	0.0135
**7-3-2-0**	0.0934	0.3074	0.1801	0.1311	0.2918	0.2256	0.3883	0.2097	0.0885	0.2975
**4-5-0-2 Iso 1**	5.4211	3.9296	2.8732	3.7768	3.9655	3.6692	1.0556	2.8655	5.6290	2.8540
**4-5-0-2 Iso 2**	58.8840	56.6065	58.8840	60.2434	63.0425	60.1393	59.3210	62.7986	57.8700	63.6476
**5-5-1-1**	0.0510	0.0583	2.0581	0.0509	0.0611	0.0631	0.3271	0.0466	0.0509	0.0712
**5-6-0-1 Iso 1**	0.0659	0.0982	0.0940	0.0507	0.0498	0.0654	0.0344	0.0934	0.0892	0.0692
**5-6-0-1 Iso 2**	0.3381	0.4689	0.2134	0.2336	0.3210	0.3372	0.2931	0.3296	0.3311	0.3230
**5-7-1-0**	0.1058	0.1363	0.0711	0.0801	0.0593	0.0812	0.0534	0.1060	0.0899	0.0925
**5-8-0-0 Iso 1**	0.4131	0.1079	0.1735	0.0487	0.1073	0.0486	0.0745	0.0660	0.4071	0.0630
**5-8-0-0 Iso 2**	0.9251	0.2737	0.4153	0.2034	0.2236	0.1107	0.1997	0.1390	0.7123	0.1137
**4-5-1-2 Iso 1**	1.5101	1.1335	1.9714	2.1173	0.3853	1.5698	1.7567	1.2811	1.6155	1.4660
**4-5-1-2 Iso 2**	0.8242	0.8835	0.8905	0.7334	0.6756	0.8671	0.8640	0.5546	0.8407	0.7829
**4-5-1-2 Iso 3**	0.4831	0.4308	0.3537	0.3662	0.4348	0.3512	0.4187	0.3571	0.4818	0.3902
**4-5-1-2 Iso 4**	6.6068	5.4742	4.2503	6.7720	6.1317	6.4235	12.6933	9.6036	6.9113	7.0063
**4-6-0-2 Iso 1**	0.0687	0.0425	0.0503	0.0796	0.0270	0.0580	0.0255	0.0452	0.0850	0.0389
**4-6-0-2 Iso 2**	0.0708	0.0477	0.0418	0.0921	0.0389	0.0483	0.0559	0.0982	0.0862	0.0831
**5-6-1-1 Iso 1**	0.0151	0.0229	0.0654	0.0137	0.0134	0.0185	0.0438	0.0175	0.0182	0.0197
**5-6-1-1 Iso 2**	0.0190	0.0157	0.0149	0.0115	0.0164	0.0146	0.0075	0.0101	0.0166	0.0184
**5-6-1-1 Iso 3**	0.0370	0.0338	0.0344	0.0233	0.0325	0.0393	0.0524	0.0314	0.0407	0.0387
**5-6-1-1 Iso 4**	0.0110	0.0090	0.0050	0.0042	0.0047	0.0070	0.0060	0.0059	0.0119	0.0059
**4-5-2-2**	0.0834	0.0287	0.0508	0.0761	0.0150	0.0420	0.1437	0.0658	0.0793	0.0367
**5-6-0-2 Iso 1**	0.1604	0.2142	0.1756	0.0989	0.1054	0.1024	0.0344	0.0669	0.1794	0.0609
**5-6-0-2 Iso 2**	0.2699	0.2518	0.1863	0.1759	0.1418	0.2738	0.1488	0.1350	0.2911	0.1861
**5-6-0-2 Iso 3**	0.2009	0.6723	0.1656	0.2930	0.4671	0.2673	0.2060	0.3779	0.2101	0.3056
**5-6-0-2 Iso 4**	0.1125	0.2016	0.1328	0.1337	0.1602	0.1725	0.1707	0.1430	0.1315	0.1418
**8-6-0-0 Iso 1**	0.2151	0.9854	0.4228	0.2638	1.3110	0.7276	0.2517	0.3602	0.2264	0.6312
**8-6-0-0 Iso 2**	0.0475	0.2442	0.1021	0.0916	0.2860	0.2925	0.3862	0.1642	0.0444	0.2304
**6-7-0-1**	0.0103	0.0186	0.0129	0.0073	0.0135	0.0134	0.0156	0.0100	0.0098	0.0069
**5-6-1-2 Iso 1**	0.1368	0.0868	0.3077	0.1152	0.0413	0.1409	0.0582	0.0786	0.1348	0.0841
**5-6-1-2 Iso 2**	0.0330	0.0272	0.0309	0.0283	0.0177	0.0278	0.0544	0.0298	0.0415	0.0201
**5-6-1-2 Iso 3**	0.0752	0.1354	0.1305	0.1804	0.0516	0.1216	0.2836	0.1526	0.0873	0.1095
**5-6-1-2 Iso 4**	0.0310	0.0355	0.0301	0.0286	0.0348	0.0411	0.0489	0.0294	0.0612	0.0462
**5-6-1-2 Iso 5**	0.0334	0.0349	0.0222	0.0280	0.0298	0.0301	0.0351	0.0305	0.0004	0.0347
**5-6-1-2 Iso 6**	0.0460	0.0509	0.0393	0.0324	0.0452	0.0426	0.0553	0.0429	0.0006	0.0441
**5-6-0-3 Iso 1**	4.0789	5.9144	3.1909	3.6749	7.2926	4.2940	1.4062	3.1820	4.6367	3.6386
**5-6-0-3 Iso 2**	1.1456	2.2481	0.9732	1.9684	2.1407	2.2416	2.4274	1.7802	1.1944	1.8674
**6-7-0-2**	0.0556	0.0766	0.0477	0.0362	0.0655	0.0544	0.0354	0.0365	0.0519	0.0304
**5-6-1-3 Iso 1**	0.3053	4.7631	0.7190	0.3447	0.1977	0.4509	0.1587	0.1605	0.3128	0.1962
**5-6-1-3 Iso 2**	4.0453	0.5901	5.4084	6.3383	2.6504	6.0621	6.8198	4.7388	4.1892	3.9242
**5-6-1-3 Iso 3**	0.4814	0.0102	0.2044	0.4510	0.6894	0.4008	0.2927	0.4252	0.4966	0.3581
**5-8-3-1 Iso 1**	0.0125	0.0123	0.0053	0.0128	0.0025	0.0096	0.0024	0.0104	0.0144	0.0125
**5-8-3-1 Iso 2**	0.0158	0.0127	0.0022	0.0081	0.0003	0.0101	0.0011	0.0206	0.0127	0.0075
**5-8-3-1 Iso 3**	0.0292	0.0379	0.0083	0.0426	0.0371	0.0293	0.0785	0.0392	0.0231	0.0272
**6-7-1-2**	0.0118	0.0129	0.0226	0.0117	0.0054	0.0168	0.0310	0.0101	0.0130	0.0087
**5-6-2-3 Iso 1**	0.1012	0.0500	0.1762	0.1437	0.0213	0.0908	0.1228	0.0547	0.1023	0.0517
**5-6-2-3 Iso 2**	0.4044	0.3654	0.1508	0.6336	0.1788	0.5050	1.1163	0.5065	0.4110	0.3479
**6-7-0-3 Iso 1**	0.0641	0.0831	0.0431	0.0357	0.0705	0.0728	0.0250	0.0300	0.0655	0.0314
**6-7-0-3 Iso 2**	0.0328	0.0392	0.0274	0.0210	0.0377	0.0335	0.0145	0.0171	0.0432	0.0185
**6-7-0-3 Iso 3**	0.0302	0.0620	0.0220	0.0224	0.0535	0.0479	0.0290	0.0263	0.0359	0.0306
**6-9-2-1**	0.0011	0.0006	0.0006	0.0013	0.0007	0.0045	0.0007	0.0028	0.0053	0.0034
**6-7-1-3 Iso 1**	0.0279	0.0325	0.0368	0.0209	0.0125	0.0366	0.0454	0.0141	0.0268	0.0135
**6-7-1-3 Iso 2**	0.0211	0.0146	0.0197	0.0142	0.0081	0.0370	0.0664	0.0236	0.0197	0.0179
**6-7-1-3 Iso 3**	0.0114	0.0251	0.0222	0.0232	0.0117	0.0371	0.0416	0.0192	0.0309	0.0135
**6-7-0-4 Iso 1**	0.1838	0.1546	0.1327	0.1078	0.1662	0.3899	0.0325	0.2457	0.4701	0.2720
**6-7-0-4 Iso 2**	0.1145	0.1487	0.0416	0.0885	0.1817	0.1569	0.0548	0.0599	0.1188	0.0786
**6-7-0-4 Iso 3**	0.0144	0.0119	0.0050	0.0035	0.0061	0.0148	0.0075	0.0063	0.0209	0.0059
**6-7-1-4 Iso 1**	0.0381	0.0292	0.0242	0.0263	0.0152	0.0435	0.0224	0.0166	0.0389	0.0157
**6-7-1-4 Iso 2**	0.0911	0.0608	0.0736	0.0626	0.0391	0.1005	0.0585	0.0253	0.0827	0.0356
**6-7-1-4 Iso 3**	0.0381	0.0235	0.0090	0.0247	0.0244	0.0301	0.0272	0.0259	0.0420	0.0230
**6-7-1-4 Iso 4**	0.0890	0.1057	0.0625	0.1116	0.0668	0.1913	0.1145	0.0666	0.1052	0.0710
**6-7-1-4 Iso 5**	0.0430	0.0318	0.0175	0.0394	0.0305	0.0516	0.0523	0.0329	0.0466	0.0257
**6-7-1-4 Iso 6**	0.0322	0.0280	0.0149	0.0285	0.0172	0.0687	0.1533	0.0272	0.0260	0.0298
**6-7-2-4 Iso 1**	0.0510	0.0285	0.1337	0.0453	0.0103	0.0683	0.0792	0.0248	0.0519	0.0232
**6-7-2-4 Iso 2**	0.0502	0.0430	0.0516	0.0880	0.0148	0.1244	0.3263	0.0510	0.0547	0.0531

Supplementary Table S3. MGC analysis, normalized abundance of all identified *N*-glycans digested from depleted low abundant serum glycoproteins of **MCI** patients. GlcNAc, Hex, Fuc, NeuAc (*N*-acetylglucosamine, Hexose, Fucose, *N*-acetylneuraminic acid), and Iso = isomer.

Glycan	MCI 1	MCI 2	MCI 3	MCI 4	MCI 5	MCI 6	MCI 7	MCI 8	MCI 9	MCI 10
**2-5-0-0**	0.2834	0.4780	0.4498	0.2971	0.1942	0.2317	0.3037	0.1262	0.3127	0.2269
**2-6-0-0**	0.8211	1.2638	1.1297	0.6215	0.5843	0.0646	0.5812	0.3519	0.7565	0.4086
**3-5-0-0**	0.0060	0.0093	0.0090	0.0059	0.0074	0.0093	0.0103	0.0111	0.0083	0.0087
**2-7-0-0**	0.0870	0.1359	0.1541	0.0737	0.0863	0.0481	0.0935	0.0718	0.1007	0.0728
**3-6-0-0**	0.0193	0.0259	0.0201	0.0155	0.0142	0.0126	0.0138	0.0122	0.0206	0.0148
**6-3-0-0**	0.0030	0.0215	0.0139	0.0065	0.0084	0.0284	0.0109	0.0492	0.0088	0.0205
**3-5-0-1**	0.0813	0.0638	0.0809	0.0547	0.0819	0.0432	0.0761	0.0566	0.0657	0.0721
**2-8-0-0 Iso 1**	0.0254	0.0334	0.0349	0.0260	0.0248	0.0133	0.0225	0.0075	0.1046	0.0234
**2-8-0-0 Iso 2**	0.1262	0.2307	0.2618	0.1211	0.1061	0.0561	0.1091	0.0652	0.0210	0.0984
**4-4-0-1**	0.0649	0.0883	0.0917	0.0578	0.0699	0.0840	0.0931	0.0710	0.0738	0.0786
**4-5-1-0**	0.0383	0.0323	0.0289	0.0183	0.0252	0.0256	0.0442	0.0457	0.0334	0.0347
**2-9-0-0**	0.4236	0.5567	0.7136	0.2997	0.3273	0.1743	0.3110	0.2172	0.3084	0.2396
**4-5-0-1**	4.8679	4.7575	5.7114	4.2085	5.8959	5.3967	5.7309	7.0733	6.7048	5.4152
**4-6-1-0**	0.5404	0.4786	0.4937	0.3981	0.5587	0.3932	0.6387	0.6549	0.3865	0.4014
**5-5-1-0**	0.0378	0.0294	0.0220	0.0164	0.0209	0.0127	0.0251	0.0194	0.0162	0.0341
**2-10-0-0**	0.0047	0.0050	0.0071	0.0019	0.0035	0.0008	0.0035	0.0031	0.0044	0.0029
**4-5-1-1 Iso 1**	0.5503	0.3689	0.2110	0.2608	0.2651	0.4474	0.4039	0.4778	0.3806	0.5229
**4-5-1-1 Iso 2**	0.4762	0.4187	0.3837	0.2473	0.2372	0.2688	0.4351	0.4355	0.3929	0.3564
**4-6-0-1**	0.0386	0.0391	0.0288	0.0471	0.0711	0.0549	0.0503	0.0548	0.0548	0.0633
**5-5-0-1**	0.1435	0.1537	0.2940	0.0312	0.0752	0.0386	0.0401	0.0990	0.0197	0.1018
**5-6-1-0**	0.0477	0.0492	0.0634	0.0152	0.0363	0.0230	0.0186	0.0262	0.0057	0.0531
**7-3-2-0**	0.3166	0.3255	0.3481	0.2884	0.2205	0.2417	0.1069	0.3493	0.1100	0.2030
**4-5-0-2 Iso 1**	6.8737	3.7390	3.9804	4.6741	4.4622	4.1103	5.0539	4.3191	4.7413	4.2374
**4-5-0-2 Iso 2**	59.5276	58.2603	55.7573	65.6223	66.4865	65.8428	57.1079	58.9837	56.9693	62.8614
**5-5-1-1**	0.0905	0.0210	0.0159	0.0179	0.0192	0.0131	0.0182	0.0057	0.0050	0.0377
**5-6-0-1 Iso 1**	0.0293	0.1013	0.1551	0.0902	0.0997	0.0662	0.0765	0.0899	0.0895	0.0763
**5-6-0-1 Iso 2**	0.1755	0.2703	0.2235	0.1701	0.3414	0.3016	0.2472	0.2318	0.2496	0.3413
**5-7-1-0**	0.0359	0.2703	0.0387	0.0486	0.0785	0.0827	0.0674	0.0429	0.0360	0.0794
**5-8-0-0 Iso 1**	0.1049	0.1472	0.0502	0.1001	0.0486	0.0431	0.1207	0.0978	0.1734	0.0745
**5-8-0-0 Iso 2**	0.1488	0.1692	0.0758	0.2025	0.1398	0.0793	0.1725	0.2193	0.2480	0.1873
**4-5-1-2 Iso 1**	1.5639	0.9171	0.2681	0.7516	0.2236	1.7712	0.6093	1.3326	0.7440	1.4505
**4-5-1-2 Iso 2**	1.2082	0.5372	0.3308	0.5278	0.6316	0.7633	0.9444	0.8560	0.7755	0.8569
**4-5-1-2 Iso 3**	0.3661	0.4728	0.4376	0.2721	0.2932	0.3514	0.5173	0.4032	0.5727	0.3628
**4-5-1-2 Iso 4**	2.3611	6.1349	4.9173	4.4043	3.0634	5.4740	5.2623	5.8268	6.6937	5.0323
**4-6-0-2 Iso 1**	0.0459	0.0208	0.0225	0.0426	0.0411	0.0481	0.0625	0.1136	0.0841	0.0633
**4-6-0-2 Iso 2**	0.0330	0.0486	0.0316	0.0515	0.0402	0.0530	0.1434	0.1691	0.1518	0.0626
**5-6-1-1 Iso 1**	0.0153	0.0148	0.0178	0.0161	0.0117	0.0089	0.0195	0.0216	0.0187	0.0229
**5-6-1-1 Iso 2**	0.0215	0.0121	0.0097	0.0082	0.0108	0.0104	0.0197	0.0131	0.0135	0.0198
**5-6-1-1 Iso 3**	0.0417	0.0434	0.0293	0.0231	0.0243	0.0274	0.0367	0.0366	0.0371	0.0313
**5-6-1-1 Iso 4**	0.0095	0.0077	0.0068	0.0054	0.0045	0.0037	0.0082	0.0061	0.0076	0.0064
**4-5-2-2**	0.0107	0.0337	0.0100	0.0158	0.0037	0.0563	0.0233	0.0768	0.0254	0.0622
**5-6-0-2 Iso 1**	0.1774	0.1544	0.3058	0.1026	0.1395	0.1055	0.2515	0.1734	0.2934	0.1553
**5-6-0-2 Iso 2**	0.1522	0.1267	0.1131	0.1245	0.1771	0.2298	0.3069	0.3006	0.2479	0.2196
**5-6-0-2 Iso 3**	0.1641	0.5521	0.7995	0.3156	0.5704	0.2176	0.3963	0.2835	0.6703	0.2710
**5-6-0-2 Iso 4**	0.0671	0.1429	0.1379	0.1017	0.1431	0.1365	0.1522	0.1417	0.1532	0.1436
**8-6-0-0 Iso 1**	1.9909	1.3263	2.4529	1.0356	1.1452	0.4991	0.6818	0.6544	0.5750	0.6273
**8-6-0-0 Iso 2**	0.2664	0.4854	0.6177	0.2500	0.1839	0.1554	0.1112	0.2220	0.1034	0.1779
**6-7-0-1**	0.0056	0.0103	0.0117	0.0056	0.0135	0.0076	0.0102	0.0100	0.0137	0.0109
**5-6-1-2 Iso 1**	0.0817	0.0667	0.0357	0.0510	0.0304	0.1079	0.0828	0.2794	0.0942	0.1402
**5-6-1-2 Iso 2**	0.0175	0.0183	0.0125	0.0137	0.0181	0.0390	0.0210	0.0279	0.0222	0.0426
**5-6-1-2 Iso 3**	0.0561	0.0984	0.0394	0.0503	0.0358	0.1051	0.0583	0.1113	0.0938	0.1153
**5-6-1-2 Iso 4**	0.0486	0.0364	0.0346	0.0254	0.0208	0.0361	0.0508	0.0422	0.0600	0.0590
**5-6-1-2 Iso 5**	0.0337	0.0316	0.0262	0.0190	0.0221	0.0278	0.0389	0.0362	0.0397	0.0307
**5-6-1-2 Iso 6**	0.0310	0.0514	0.0460	0.0311	0.0339	0.0342	0.0523	0.0563	0.0714	0.0413
**5-6-0-3 Iso 1**	7.4523	6.0140	10.8348	6.3361	7.3109	3.4140	9.3435	6.9491	7.9141	4.4848
**5-6-0-3 Iso 2**	1.4053	2.8727	2.9837	2.2017	2.0402	1.4541	2.2475	1.1881	2.1548	1.5379
**6-7-0-2**	0.0365	0.0440	0.0590	0.0249	0.0481	0.0345	0.0743	0.0430	0.0661	0.0492
**5-6-1-3 Iso 1**	0.2558	0.3212	0.1564	0.2079	0.0807	0.2708	0.1643	0.6376	0.1582	0.3820
**5-6-1-3 Iso 2**	4.7291	4.7101	1.8722	3.2158	1.4437	4.5971	3.4770	4.0243	3.5876	5.3331
**5-6-1-3 Iso 3**	0.1531	0.5376	1.0168	0.3689	0.2842	0.2729	0.8839	0.3071	0.8860	0.3646
**5-8-3-1 Iso 1**	0.0004	0.0098	0.0145	0.0074	0.0049	0.0087	0.0167	0.0073	0.0139	0.0098
**5-8-3-1 Iso 2**	0.0004	0.0268	0.0104	0.0146	0.0081	0.0092	0.0144	0.0097	0.0136	0.0168
**5-8-3-1 Iso 3**	0.0100	0.0361	0.0291	0.0232	0.0152	0.0203	0.0331	0.0159	0.0196	0.0213
**6-7-1-2**	0.0051	0.0076	0.0025	0.0037	0.0037	0.0091	0.0154	0.0074	0.0136	0.0089
**5-6-2-3 Iso 1**	0.1108	0.0391	0.0143	0.0367	0.0104	0.0874	0.0220	0.0370	0.0347	0.1053
**5-6-2-3 Iso 2**	0.1031	0.3991	0.1471	0.1538	0.0456	0.2583	0.2736	0.2547	0.3282	0.3455
**6-7-0-3 Iso 1**	0.0622	0.0554	0.0809	0.0399	0.0311	0.0384	0.1157	0.0417	0.0713	0.0560
**6-7-0-3 Iso 2**	0.0339	0.0290	0.0353	0.0215	0.0587	0.0239	0.0633	0.0338	0.0387	0.0382
**6-7-0-3 Iso 3**	0.0254	0.0474	0.0527	0.0373	0.0590	0.0310	0.0676	0.0332	0.0537	0.0346
**6-9-2-1**	0.0006	0.0053	0.0028	0.0041	0.0074	0.0036	0.0062	0.0019	0.0032	0.0039
**6-7-1-3 Iso 1**	0.0204	0.0187	0.0058	0.0106	0.0068	0.0216	0.0231	0.0228	0.0152	0.0257
**6-7-1-3 Iso 2**	0.0103	0.0182	0.0063	0.0091	0.0073	0.0226	0.0179	0.0236	0.0148	0.0149
**6-7-1-3 Iso 3**	0.0075	0.0079	0.0044	0.0060	0.0034	0.0120	0.0096	0.0224	0.0165	0.0232
**6-7-0-4 Iso 1**	0.3288	0.5165	0.8608	0.5774	0.7010	0.3167	0.4389	0.0655	0.1693	0.1674
**6-7-0-4 Iso 2**	0.1302	0.1070	0.1370	0.1485	0.1987	0.1182	0.4178	0.1764	0.1968	0.1757
**6-7-0-4 Iso 3**	0.0111	0.0108	0.0101	0.0139	0.0163	0.0116	0.0251	0.0202	0.0139	0.0113
**6-7-1-4 Iso 1**	0.0376	0.0150	0.0055	0.0208	0.0114	0.0380	0.0389	0.0322	0.0181	0.0363
**6-7-1-4 Iso 2**	0.0810	0.0409	0.0149	0.0375	0.0175	0.0728	0.0804	0.0576	0.0430	0.0744
**6-7-1-4 Iso 3**	0.0398	0.0248	0.0106	0.0256	0.0273	0.0339	0.0583	0.0554	0.0447	0.0431
**6-7-1-4 Iso 4**	0.0729	0.0824	0.0294	0.0704	0.0385	0.1169	0.0828	0.0641	0.0464	0.1007
**6-7-1-4 Iso 5**	0.0356	0.0306	0.0155	0.0263	0.0236	0.0428	0.0511	0.0358	0.0370	0.0397
**6-7-1-4 Iso 6**	0.0110	0.0258	0.0074	0.0211	0.0102	0.0367	0.0234	0.0283	0.0180	0.0286
**6-7-2-4 Iso 1**	0.0362	0.0213	0.0050	0.0207	0.0056	0.0572	0.0261	0.0369	0.0161	0.0552
**6-7-2-4 Iso 2**	0.0347	0.0371	0.0118	0.0313	0.0078	0.0887	0.0405	0.3036	0.0276	0.0575

Supplementary Table S4. C18(50cm) analysis, normalized abundance of all identified *N*-glycans digested from depleted low abundant serum glycoproteins of **control** patients. GlcNAc, Hex, Fuc, NeuAc (*N*-acetylglucosamine, Hexose, Fucose, *N*-acetylneuraminic acid), and Iso = isomer.

Glycan	Control 1	Control 2	Control 3	Control 4	Control 5	Control 6	Control 7	Control 8	Control 9	Control 10
**2-5-0-0**	0.5207	0.2402	0.6173	0.3578	0.4175	0.3519	0.2824	0.2020	0.3664	0.3322
**2-6-0-0**	1.0101	1.8573	1.1053	1.2868	1.2801	1.5172	1.2431	0.9616	1.3941	1.4963
**2-7-0-0**	0.1958	0.1462	0.1455	0.0905	0.1036	0.0985	0.2029	0.1496	0.1451	0.0931
**2-8-0-0**	0.4715	0.6680	0.4595	0.5209	0.5148	0.6103	0.4239	0.4886	0.5396	0.5998
**2-9-0-0**	0.6322	1.0261	0.5303	0.7732	1.0954	0.9453	0.6553	0.7816	0.7966	1.0193
**3-4-0-0**	0.0015	0.0037	0.0059	0.0023	0.0019	0.0015	0.0010	0.0019	0.0035	0.0025
**3-4-0-1**	0.2317	0.2311	0.3716	0.1676	0.1529	0.1454	0.0893	0.0545	0.1032	0.1726
**3-4-1-0**	0.0070	0.0036	0.0112	0.0027	0.0028	0.0026	0.0032	0.0526	0.0032	0.0028
**3-4-1-1**	0.0798	0.1512	0.1038	0.1443	0.1978	0.1815	0.1564	0.1771	0.1863	0.0929
**3-5-0-1**	0.2188	0.1292	0.2019	0.1527	0.1077	0.0025	0.0027	0.0034	0.0023	0.0035
**3-5-1-0**	0.0174	0.0181	0.0276	0.0118	0.0131	0.0099	0.0053	0.0026	0.0041	0.0082
**3-5-2-1**	0.0000	0.0194	0.0000	0.0382	0.0128	0.0077	0.0044	0.0816	0.0233	0.0158
**3-6-0-0**	0.0159	0.0040	0.0142	0.0023	0.0031	0.0029	0.0044	0.0017	0.0041	0.0029
**3-6-0-1**	0.0230	0.0515	0.0100	0.0258	0.0221	0.0132	0.0266	0.0286	0.0594	0.0237
**3-6-1-0**	0.0000	0.0412	0.0065	0.0192	0.0175	0.0004	0.0006	0.0010	0.0014	0.0008
**3-7-1-0**	0.0318	0.1073	0.0203	0.0522	0.0430	0.0336	0.0601	0.0906	0.1705	0.0705
**4-3-0-0**	0.0255	0.0016	0.1456	0.0011	0.0000	0.0005	0.0008	0.0006	0.0020	0.0010
**4-4-0-0**	0.0820	0.0173	0.2494	0.0064	0.0086	0.0047	0.0036	0.0044	0.0106	0.0080
**4-4-0-1**	0.3915	0.4812	0.4125	0.2294	0.2128	0.1786	0.0933	0.1140	0.2619	0.2851
**4-4-1-0**	3.9298	0.0237	4.8619	0.0089	0.0145	0.0153	0.0271	0.0061	0.0152	0.0127
**4-4-2-1 Iso 1**	0.0000	0.0057	0.0119	0.0014	0.0083	0.0087	0.0090	0.0027	0.0010	0.0078
**4-4-2-1 Iso 2**	0.0000	0.0026	0.0050	0.0024	0.0075	0.0078	0.0067	0.0023	0.0010	0.0073
**4-4-3-0**	0.0317	0.0186	0.0156	0.0122	0.0107	0.0192	0.3305	0.3208	0.2255	0.0594
**4-5-0-0**	0.1121	0.0460	0.1145	0.0267	0.0326	0.0198	0.0176	0.0180	0.0381	0.0256
**4-5-0-1**	4.8904	5.5832	4.4349	3.6175	3.4414	2.9725	2.1329	3.7549	5.2735	4.1911
**4-5-0-2**	43.1119	47.2432	42.2994	51.6054	48.8393	49.1119	43.8921	51.6657	50.0148	52.6853
**4-5-1-0 Iso 1**	0.0475	0.0549	0.0285	0.0279	0.0255	0.0228	0.0115	0.0124	0.0228	0.0291
**4-5-1-0 Iso 2**	1.4118	0.1176	1.2243	0.0692	0.0891	0.0850	0.1044	0.0454	0.1539	0.1059
**4-5-1-1 Iso 1**	1.7764	0.5876	1.4421	0.3935	0.4450	0.5110	0.7380	0.4064	0.4867	0.4013
**4-5-1-1 Iso 2**	0.2206	0.2670	0.3627	0.2874	0.1875	0.2651	0.3586	0.2389	0.3452	0.3085
**4-5-1-2 Iso 1**	6.4481	8.3752	6.3063	10.8751	8.7597	9.7987	15.8646	13.1195	10.6507	10.4746
**4-5-1-2 Iso 2**	0.4998	0.9759	1.1560	1.0847	1.0525	1.1956	1.1360	0.8129	1.0756	1.0632
**4-5-3-2**	0.2458	0.3001	0.2813	0.0739	0.4084	0.3242	0.1733	0.1295	0.0956	0.2992
**4-6-2-0 Iso 1**	0.2632	0.1048	0.1685	0.0481	0.0005	0.0752	0.0966	0.0591	0.0635	0.0573
**4-6-2-0 Iso 2**	0.0168	0.0331	0.0546	0.0356	0.0001	0.0328	0.0446	0.0276	0.0374	0.0250
**4-6-1-0**	0.6362	0.7036	0.4222	0.3299	0.3306	0.2876	0.1990	0.3680	0.5255	0.4727
**4-6-0-1**	0.0434	0.0868	0.0431	0.0747	0.0608	0.0691	0.0571	0.0685	0.0511	0.0677
**4-6-0-2**	0.0676	0.1363	0.1149	0.2362	0.1293	0.1154	0.0943	0.1364	0.1371	0.1280
**4-6-1-1**	2.5122	4.4622	3.1686	4.4029	4.4025	4.4992	3.5953	4.3033	3.8769	4.1207
**4-6-2-1**	0.6957	1.1273	0.7359	1.2154	1.1798	1.1495	1.7171	1.4543	1.0754	1.1450
**5-3-0-0**	0.2220	0.0057	0.5354	0.0020	0.0036	0.0055	0.0080	0.0017	0.0068	0.0026
**5-3-1-0**	0.5488	0.0130	2.3892	0.0103	0.0117	0.0143	0.0189	0.0063	0.0221	0.0144
**5-4-0-1**	0.0000	0.0286	0.0048	0.0303	0.0241	0.0122	0.0104	0.0282	0.0351	0.0258
**5-4-1-0**	0.8144	0.0370	1.7426	0.0219	0.0182	0.0295	0.0563	0.0233	0.0457	0.0357
**5-4-1-1**	0.1445	0.0115	0.3551	0.0080	0.0075	0.0110	0.0396	0.0085	0.0088	0.0086
**5-5-0-0**	0.0529	0.0020	0.0970	0.0018	0.0028	0.0026	0.0061	0.0018	0.0042	0.0032
**5-5-0-1**	0.0000	0.0895	0.1265	0.0457	0.0325	0.0937	0.0241	0.0097	0.0344	0.0424
**5-5-0-2 Iso 1**	0.0478	0.0456	0.0268	0.0236	0.0324	0.0204	0.0066	0.0098	0.0160	0.0137
**5-5-0-2 Iso 2**	0.0929	0.0139	0.1478	0.0076	0.0158	0.0145	0.4476	0.0247	0.0043	0.0292
**5-5-1-0**	0.0255	0.0016	0.1452	0.0011	0.0007	0.0005	0.0008	0.0006	0.0020	0.0010
**5-5-1-1**	1.4314	0.0738	2.4277	0.0706	0.0904	0.0912	0.5112	0.0735	0.0810	0.1079
**5-5-1-2**	2.2364	0.0881	2.1130	0.0898	0.1215	0.0997	0.7127	0.1021	0.0732	0.1115
**5-6-0-1 Iso 1**	0.1630	0.1523	0.0665	0.0009	0.0014	0.0493	0.0330	0.0463	0.0591	0.0390
**5-6-0-1 Iso 2**	0.3154	0.3219	0.2042	0.0020	0.0035	0.2781	0.2897	0.2600	0.2975	0.2225
**5-6-0-3 Iso 1**	2.1983	3.0772	2.1500	3.1926	3.8637	3.5474	4.2321	3.2258	2.2887	3.0114
**5-6-0-3 Iso 2**	6.8239	6.4728	4.0238	4.7425	0.4874	5.0151	2.1422	4.1076	5.5989	4.3895
**5-6-0-3 Iso 3**	1.3149	0.7673	0.5100	0.4907	8.8274	0.4226	0.2892	0.3392	0.7544	0.3649
**5-6-0-3 Iso 4**	0.4888	0.4691	0.4214	0.3952	0.8408	0.5839	0.2865	0.3961	0.4031	0.3323
**5-6-0-4 Iso 1**	0.0575	0.0078	0.0099	0.0177	0.0171	0.0095	0.0052	0.0127	0.0085	0.0126
**5-6-0-4 Iso 2**	0.1038	0.0033	0.0056	0.0049	0.0054	0.0045	0.0073	0.0052	0.0079	0.0038
**5-6-1-1**	0.1300	0.1563	0.1509	0.1239	0.1355	0.1848	0.2572	0.1431	0.1614	0.1744
**5-6-1-2 Iso 1**	0.0359	0.0643	0.0220	0.0577	0.0635	0.0419	0.0887	0.0814	0.0572	0.0516
**5-6-1-2 Iso 2**	0.3012	0.3261	0.4056	0.4348	0.1585	0.3172	0.6238	0.3592	0.3226	0.2992
**5-6-1-2 Iso 3**	0.0628	0.1125	0.1054	0.1098	0.0849	0.1539	0.2150	0.1249	0.1421	0.1576
**5-6-1-2 Iso 4**	0.0616	0.0356	0.0263	0.0332	0.0370	0.0420	0.0495	0.0331	0.0499	0.0453
**5-6-1-3 Iso 1**	0.1336	0.3380	0.1046	0.4223	0.4056	0.2958	0.7900	0.4201	0.2490	0.2984
**5-6-1-3 Iso 2**	4.1108	4.3758	4.8011	5.5938	3.4430	5.4948	5.7252	4.6276	4.7106	4.4399
**5-6-1-3 Iso 3**	0.1340	0.1941	0.1695	0.1838	0.1563	0.1020	0.1408	0.0849	0.1504	0.1311
**5-6-2-3 Iso 1**	0.2074	0.5066	0.2581	0.8642	0.3319	0.6755	1.5218	0.7202	0.6711	0.5329
**5-6-2-3 Iso2**	0.1216	0.1738	0.3051	0.3372	0.0508	0.2381	0.2533	0.1500	0.2427	0.1499
**5-7-1-0**	0.0034	0.0068	0.0000	0.0009	0.0013	0.0011	0.0008	0.0015	0.0130	0.0010
**5-7-1-1**	0.1163	0.1536	0.0689	0.0839	0.1366	0.1079	0.0827	0.1038	0.0845	0.0993
**5-7-2-0**	0.0095	0.0112	0.0091	0.0056	0.0082	0.0131	0.0167	0.0097	0.0074	0.0122
**5-7-1-2 Iso 1**	0.9874	0.3023	0.2519	0.3661	0.4037	0.7140	0.5908	0.5785	0.5178	0.5389
**5-7-1-2 Iso2**	0.0745	0.0727	0.0665	0.0559	0.0808	0.0867	0.0512	0.0568	0.0648	0.0602
**6-4-1-1**	0.4007	0.2252	0.0857	0.1223	0.0726	0.1093	0.2173	0.1363	0.2033	0.0862
**6-5-1-0**	0.0000	0.0109	0.0056	0.0013	0.0112	0.0055	0.0073	0.0055	0.0028	0.0027
**6-6-0-2**	0.0294	0.0426	0.0440	0.0141	0.0148	0.0723	0.0000	0.0000	0.0266	0.0246
**6-6-1-2**	0.0000	0.0142	0.0302	0.0119	0.0037	0.0326	0.0177	0.0029	0.0059	0.0066
**6-6-1-3**	0.0095	0.0119	0.0146	0.0127	0.0118	0.0143	0.0726	0.0194	0.0095	0.0148
**6-7-0-1**	0.0566	0.0764	0.0341	0.0324	0.0578	0.0535	0.0663	0.0445	0.0384	0.0348
**6-7-0-2**	0.2638	0.2394	0.1444	0.1369	0.2094	0.2160	0.1957	0.1506	0.2027	0.1304
**6-7-0-3**	0.5703	0.5656	0.2778	0.2887	0.5878	0.4793	0.3264	0.2989	0.4404	0.3059
**6-7-0-4 Iso 1**	0.0169	0.0237	0.0085	0.0207	0.0336	0.0450	0.0408	0.0223	0.0178	0.0265
**6-7-0-4 Iso 2**	0.0552	0.0620	0.0212	0.0383	0.0544	0.0699	0.0307	0.0356	0.0674	0.0439
**6-7-0-4 Iso 3**	0.0517	0.0359	0.0250	0.0250	0.0267	0.0367	0.0120	0.0171	0.0555	0.0195
**6-7-1-1**	0.0125	0.0189	0.0120	0.0118	0.0129	0.0189	0.0427	0.0101	0.0147	0.0198
**6-7-1-2 Iso 1**	0.0776	0.0782	0.0825	0.0709	0.0540	0.0864	0.1622	0.0675	0.0755	0.0670
**6-7-1-2 Iso 2**	0.0040	0.0160	0.0103	0.0156	0.0163	0.0214	0.0250	0.0177	0.0306	0.0311
**6-7-1-2 Iso 3**	0.0000	0.0049	0.0022	0.0048	0.0028	0.0056	0.0061	0.0054	0.0070	0.0067
**6-7-1-3**	0.2057	0.2672	0.2507	0.2649	0.1252	0.3112	0.4906	0.2358	0.2641	0.2001

Supplementary Table S4. Continued…

Glycan	Control 1	Control 2	Control 3	Control 4	Control 5	Control 6	Control 7	Control 8	Control 9	Control 10
**6-7-1-4 Iso 1**	0.0425	0.1063	0.2078	0.1643	0.1638	0.2613	0.5521	0.1535	0.1100	0.1437
**6-7-1-4 Iso 2**	0.1937	0.2966	0.0729	0.3454	0.2285	0.5006	0.4312	0.2462	0.3709	0.2595
**6-7-1-4 Iso 3**	0.1679	0.1990	0.1903	0.2249	0.0847	0.3227	0.1780	0.1727	0.3721	0.1747
**6-7-2-4 Iso 1**	0.0384	0.0992	0.0955	0.1939	0.0491	0.2468	0.6319	0.1350	0.1640	0.1285
**6-7-2-4 Iso 2**	0.0894	0.0986	0.1451	0.1986	0.0468	0.2312	0.3201	0.0995	0.1946	0.1008
**6-7-3-4**	0.0296	0.0299	0.0708	0.0530	0.0083	0.1016	0.1629	0.0315	0.0737	0.0358
**6-7-1-0**	0.0000	0.0128	0.0049	0.0052	0.0107	0.0089	0.0109	0.0081	0.0056	0.0043
**6-8-1-1**	0.0000	0.0220	0.0124	0.0121	0.0149	0.0236	0.0396	0.0126	0.0118	0.0149
**7-8-0-4**	0.0281	0.0305	0.0150	0.0199	0.0417	0.0326	0.0145	0.0283	0.0187	0.0188
**7-8-1-3**	0.0000	0.0257	0.0149	0.0219	0.0182	0.0262	0.0350	0.0271	0.0218	0.0273
**7-8-1-4**	0.0000	0.0293	0.0148	0.0314	0.0274	0.0376	0.0338	0.0295	0.0324	0.0233
**7-8-2-4 Iso 1**	0.0000	0.0028	0.0026	0.0046	0.0011	0.0047	0.0120	0.0044	0.0023	0.0022
**7-8-2-4 Iso 2**	0.0000	0.0085	0.0067	0.0077	0.0009	0.0185	0.0185	0.0072	0.0078	0.0061
**8-6-0-0**	1.7843	2.1404	1.2561	0.9521	3.0287	1.9990	1.4938	1.1066	0.9833	1.5741
**8-6-0-1 Iso 1**	0.3339	0.3791	0.1876	0.1461	0.5340	0.2437	0.1363	0.0983	0.1098	0.2152
**8-6-0-1 Iso 2**	0.7326	0.5008	0.3272	0.1380	0.6376	0.3968	0.2887	0.1882	0.4059	0.2675
**8-6-0-1 Iso 3**	0.2999	0.3100	0.3687	0.2451	0.2075	0.4406	0.4918	0.2301	0.1821	0.2859
**8-6-0-1 Iso 4**	0.0163	0.0496	0.0199	0.0176	0.0635	0.0569	0.0348	0.0235	0.0303	0.0141

Supplementary Table S5. C18(50cm) analysis, normalized abundance of all identified *N*-glycans digested from depleted low abundant serum glycoproteins of **MCI** patients. GlcNAc, Hex, Fuc, NeuAc (*N*-acetylglucosamine, Hexose, Fucose, *N*-acetylneuraminic acid), and Iso = isomer.

Glycan	MCI 1	MCI 2	MCI 3	MCI 4	MCI 5	MCI 6	MCI 7	MCI 8	MCI 9	MCI 10
**2-5-0-0**	0.2266	0.3199	0.3300	0.2718	0.2043	0.2601	0.3154	0.1561	0.2414	0.2042
**2-6-0-0**	1.1924	1.5767	1.4700	1.0978	0.9745	0.7014	0.9625	0.5624	1.3317	0.8809
**2-7-0-0**	0.1177	0.0874	0.0676	0.0922	0.0998	0.0617	0.0917	0.0634	0.0864	0.1084
**2-8-0-0**	0.4346	0.6515	0.6902	0.4386	0.3962	0.2717	0.4287	0.2479	0.3721	0.3999
**2-9-0-0**	0.7381	0.8056	1.1366	0.6320	0.7422	0.3956	0.7065	0.4159	0.6706	0.5210
**3-4-0-0**	0.0015	0.0037	0.0002	0.0026	0.0012	0.0020	0.0025	0.0011	0.0017	0.0009
**3-4-0-1**	0.1443	0.3236	0.3434	0.1762	0.1179	0.1149	0.1270	0.3180	0.1418	0.0863
**3-4-1-0**	0.0032	0.0042	0.0042	0.0026	0.0023	0.0018	0.0032	0.0023	0.0028	0.0022
**3-4-1-1**	0.1415	0.1624	0.1934	0.0002	0.1616	0.1011	0.1195	0.1106	0.1373	0.1250
**3-5-0-1**	0.1285	0.1136	0.1304	0.0952	0.1247	0.0798	0.1096	0.0689	0.1128	0.1282
**3-5-1-0**	0.0059	0.0153	0.0148	0.0077	0.0052	0.0035	0.0044	0.0018	0.0029	0.0017
**3-5-2-1**	0.0049	0.0112	0.0131	0.0161	0.0157	0.0210	0.0624	0.0934	0.0577	0.0146
**3-6-0-0**	0.0034	0.0035	0.0003	0.0027	0.0032	0.0034	0.0036	0.0047	0.0027	0.0025
**3-6-0-1**	0.0233	0.0002	0.0437	0.0001	0.0204	0.0252	0.3937	0.5615	0.1372	0.0183
**3-6-1-0**	0.0130	0.0250	0.0247	0.0001	0.0227	0.0254	0.2500	0.6413	0.0863	0.0136
**3-7-1-0**	0.0345	0.0601	0.0029	0.0703	0.0452	0.0794	0.5778	1.5493	0.2269	0.0494
**4-3-0-0**	0.0005	0.0015	0.0016	0.0005	0.0005	0.0008	0.0006	0.0007	0.0007	0.0002
**4-4-0-0**	0.0075	0.0128	0.0145	0.0070	0.0062	0.0052	0.0078	0.0069	0.0098	0.0054
**4-4-0-1**	0.2604	0.0003	0.4187	0.2575	0.2131	0.2829	0.2248	0.1944	0.2705	0.2155
**4-4-1-0**	0.0141	0.0007	0.0146	0.0065	0.0062	0.0062	0.0090	0.0023	0.0124	0.0100
**4-4-2-1 Iso 1**	0.0114	0.0012	0.0066	0.0080	0.0047	0.0035	0.0005	0.0000	0.0002	0.0082
**4-4-2-1 Iso 2**	0.0082	0.0032	0.0071	0.0059	0.0048	0.0026	0.0004	0.0000	0.0001	0.0047
**4-4-3-0**	0.1869	0.8033	0.1476	0.0135	0.0109	0.0278	0.1557	0.0494	0.0116	0.0778
**4-5-0-0**	0.0221	0.0255	0.0393	0.0232	0.0202	0.0155	0.0261	0.0317	0.0278	0.0178
**4-5-0-1**	3.3718	3.8479	0.0007	3.6967	3.9024	4.1832	3.8480	4.8491	4.4665	3.7000
**4-5-0-2**	50.2798	47.2169	46.2701	62.2760	53.1840	54.0171	46.3499	50.2571	47.2017	51.6217
**4-5-1-0 Iso 1**	0.0291	0.0430	0.0468	0.0287	0.0238	0.0212	0.0235	0.0188	0.0257	0.0126
**4-5-1-0 Iso 2**	0.1147	0.0944	0.0815	0.0534	0.0477	0.0519	0.0933	0.0858	0.0872	0.0929
**4-5-1-1 Iso 1**	0.5397	0.4686	0.4828	0.3749	0.2764	0.3030	0.4906	0.4752	0.3986	0.4185
**4-5-1-1 Iso 2**	0.3136	0.2364	0.1579	0.1476	0.1389	0.2547	0.1854	0.3265	0.1827	0.2707
**4-5-1-2 Iso 1**	5.5144	8.8990	7.9021	8.8621	4.6857	9.5074	9.1219	9.7620	10.5435	9.2318
**4-5-1-2 Iso 2**	1.5266	0.9533	1.0277	0.8906	0.7984	1.1490	1.2942	1.2422	0.9536	1.2344
**4-5-3-2**	0.4400	0.4145	0.4996	0.3165	0.2294	0.1680	0.0514	0.2018	0.0650	0.2662
**4-6-2-0 Iso 1**	0.0788	0.0810	0.0737	0.0669	0.0327	0.0271	0.0826	0.0637	0.0711	0.0474
**4-6-2-0 Iso 2**	0.0421	0.0213	0.0107	0.0139	0.0163	0.0269	0.0273	0.0439	0.0250	0.0516
**4-6-1-0**	0.3551	0.4742	0.6240	0.3630	0.3760	0.3983	0.4806	0.5262	0.5677	0.3376
**4-6-0-1**	0.0420	0.0008	0.0594	0.0656	0.0719	0.0610	0.0467	0.0463	0.0453	0.0558
**4-6-0-2**	0.1500	0.1391	0.1263	0.1916	0.1403	0.1841	0.3165	0.3503	0.3204	0.1740
**4-6-1-1**	4.2309	3.7644	3.5073	0.0012	4.0809	4.2424	3.5304	4.0240	3.9077	4.1201
**4-6-2-1**	0.5357	1.1539	1.0307	0.4190	0.9267	0.8076	1.0713	0.9247	1.0082	0.8091
**5-3-0-0**	0.0028	0.0001	0.0029	0.0004	0.0043	0.0020	0.0046	0.0008	0.0031	0.0040
**5-3-1-0**	0.0197	0.0181	0.0113	0.0002	0.0131	0.0101	0.0155	0.0145	0.0110	0.0177
**5-4-0-1**	0.0260	0.0010	0.0181	0.0108	0.0245	0.0180	0.0707	0.0636	0.0356	0.0194
**5-4-1-0**	0.0427	0.0394	0.0276	0.0011	0.0274	0.0174	0.0311	0.0219	0.0199	0.0466
**5-4-1-1**	0.0125	0.0127	0.0086	0.0085	0.0113	0.0084	0.0082	0.0072	0.0038	0.0128
**5-5-0-0**	0.0050	0.0043	0.0024	0.0031	0.0016	0.0022	0.0030	0.0017	0.0016	0.0042
**5-5-0-1**	0.1673	0.1779	0.3306	0.0828	0.0675	0.0488	0.0399	0.1236	0.0288	0.1057
**5-5-0-2 Iso 1**	0.0427	0.0720	0.1160	0.0023	0.0372	0.0577	0.0457	0.0188	0.0444	0.0222
**5-5-0-2 Iso 2**	0.0042	0.0197	0.0052	0.0026	0.0037	0.0057	0.0168	0.0071	0.0301	0.0126
**5-5-1-0**	0.0003	0.0016	0.0017	0.0005	0.0005	0.0007	0.0006	0.0007	0.0010	0.0001
**5-5-1-1**	0.1374	0.0981	0.0000	0.0768	0.0789	0.0620	0.0818	0.0736	0.0599	0.1355
**5-5-1-2**	0.1640	0.1358	0.0975	0.0964	0.1388	0.0983	0.1073	0.0524	0.0677	0.1427
**5-6-0-1 Iso 1**	0.0427	0.0930	0.1351	0.0066	0.0451	0.0448	0.0696	0.0760	0.1302	0.0538
**5-6-0-1 Iso 2**	0.1388	0.1861	0.1462	0.0115	0.1639	0.2564	0.1903	0.2533	0.2207	0.2777
**5-6-0-3 Iso 1**	2.5078	3.6486	3.5958	2.7171	3.9472	2.5614	3.3272	2.4468	3.1452	2.9371
**5-6-0-3 Iso 2**	8.2807	6.7659	11.4784	7.1825	8.9562	4.4662	9.3959	4.2800	8.6218	6.1886
**5-6-0-3 Iso 3**	1.5196	0.7287	1.5655	0.8978	0.9973	0.8445	1.3631	0.4350	1.2372	0.6294
**5-6-0-3 Iso 4**	0.2928	0.2232	0.1391	0.2991	0.1344	0.6568	0.6623	0.4009	0.1028	0.4457
**5-6-0-4 Iso 1**	0.0201	0.0193	0.0272	0.0182	0.0153	0.0673	0.0108	0.0080	0.0184	0.0248
**5-6-0-4 Iso 2**	0.0053	0.0048	0.0032	0.0061	0.0065	0.1538	0.0045	0.0047	0.0041	0.0059
**5-6-1-1**	0.1664	0.1470	0.1077	0.0803	0.0997	0.1241	0.1445	0.1338	0.1282	0.1507
**5-6-1-2 Iso 1**	0.0205	0.0685	0.0810	0.0316	0.0450	0.0346	0.0701	0.0836	0.1163	0.0437
**5-6-1-2 Iso 2**	0.2359	0.2985	0.1589	0.1039	0.2013	0.3520	0.2368	0.4179	0.3128	0.3973
**5-6-1-2 Iso 3**	0.1345	0.0924	0.0853	0.0780	0.0793	0.1404	0.1156	0.1198	0.1168	0.1276
**5-6-1-2 Iso 4**	0.0609	0.0320	0.0323	0.0372	0.0296	0.0357	0.0490	0.0451	0.0408	0.0439
**5-6-1-3 Iso 1**	0.0759	0.4207	0.4479	0.1342	0.2821	0.2239	0.3958	0.2719	0.4064	0.2012
**5-6-1-3 Iso 2**	4.9957	5.4075	3.6515	1.8385	4.5662	5.1856	4.7674	4.9081	5.1071	5.7311
**5-6-1-3 Iso 3**	0.1895	0.1000	0.0986	0.1077	0.1101	0.3701	0.3129	0.3369	0.1200	0.3108
**5-6-2-3 Iso 1**	0.1867	0.6085	0.2811	0.0763	0.2275	0.4507	0.4519	0.5524	0.5869	0.5611
**5-6-2-3 Iso2**	0.2717	0.1870	0.0717	0.0251	0.0272	0.2481	0.1220	0.1884	0.1578	0.2511
**5-7-1-0**	0.0015	0.0055	0.0058	0.0016	0.0014	0.0001	0.0022	0.0016	0.0051	0.0003
**5-7-1-1**	0.0602	0.1304	0.1495	0.1068	0.0962	0.0716	0.1189	0.0998	0.1334	0.0772
**5-7-2-0**	0.0117	0.0106	0.0086	0.0053	0.0078	0.0051	0.0124	0.0055	0.0077	0.0063
**5-7-1-2 Iso 1**	0.9502	0.8926	1.1367	0.7443	1.0363	0.5493	0.9882	0.4401	0.7328	0.6442
**5-7-1-2 Iso2**	0.0892	0.0571	0.0587	0.0637	0.0729	0.0856	0.0869	0.0507	0.0447	0.0704
**6-4-1-1**	0.1019	0.0817	0.1077	0.1450	0.1502	0.0775	0.1452	0.0605	0.1452	0.1000
**6-5-1-0**	0.0088	0.0218	0.0061	0.0010	0.0003	0.0006	0.0009	0.0008	0.0005	0.0021
**6-6-0-2**	0.1003	0.1243	0.2669	0.0575	0.0415	0.0267	0.0382	0.0733	0.0211	0.0541
**6-6-1-2**	0.0225	0.0302	0.0160	0.0023	0.0027	0.0080	0.0060	0.0246	0.0027	0.0177
**6-6-1-3**	0.0241	0.0201	0.0107	0.0116	0.0213	0.0142	0.0167	0.0086	0.0174	0.0156
**6-7-0-1**	0.0161	0.0442	0.0489	0.0417	0.0225	0.0246	0.0380	0.0381	0.0442	0.0389
**6-7-0-2**	0.1111	0.1557	0.1769	0.1605	0.1073	0.1365	0.2085	0.1276	0.1914	0.1828
**6-7-0-3**	0.3696	0.4248	0.5729	0.5100	0.3623	0.3329	0.7025	0.3207	0.4988	0.4166
**6-7-0-4 Iso 1**	0.0190	0.0256	0.0219	0.0278	0.0267	0.0245	0.0376	0.0152	0.0212	0.0207
**6-7-0-4 Iso 2**	0.0715	0.0539	0.0659	0.0713	0.0806	0.0542	0.1031	0.0376	0.0620	0.0598
**6-7-0-4 Iso 3**	0.0781	0.0311	0.0420	0.0465	0.0418	0.0324	0.0894	0.0252	0.0440	0.0430
**6-7-1-1**	0.0170	0.0119	0.0004	0.0094	0.0126	0.0109	0.0107	0.0157	0.0119	0.0174
**6-7-1-2 Iso 1**	0.0506	0.0592	0.0326	0.0317	0.0465	0.0706	0.0608	0.0761	0.0631	0.0781

Supplementary Table S5. Continued…

Glycan	MCI 1	MCI 2	MCI 3	MCI 4	MCI 5	MCI 6	MCI 7	MCI 8	MCI 9	MCI 10
**6-7-1-2 Iso 2**	0.0366	0.0208	0.0198	0.0210	0.0208	0.0227	0.0206	0.0278	0.0260	0.0281
**6-7-1-2 Iso 3**	0.0076	0.0044	0.0038	0.0045	0.0048	0.0056	0.0067	0.0065	0.0060	0.0057
**6-7-1-3**	0.2103	0.2627	0.1507	0.1120	0.1734	0.3004	0.2466	0.2875	0.2438	0.2856
**6-7-1-4 Iso 1**	0.0716	0.1168	0.0378	0.0377	0.0973	0.1645	0.1159	0.0907	0.0885	0.1331
**6-7-1-4 Iso 2**	0.2807	0.2799	0.1150	0.1309	0.3038	0.4081	0.3602	0.2640	0.2612	0.3485
**6-7-1-4 Iso 3**	0.3730	0.1960	0.0900	0.1282	0.2144	0.3185	0.3454	0.2468	0.2447	0.3384
**6-7-2-4 Iso 1**	0.0730	0.1077	0.0168	0.0172	0.0953	0.2163	0.0753	0.1031	0.0727	0.1920
**6-7-2-4 Iso 2**	0.1375	0.0990	0.0204	0.0209	0.0879	0.1999	0.0844	0.1145	0.0728	0.2034
**6-7-3-4**	0.0397	0.0352	0.0049	0.0054	0.0259	0.0843	0.0280	0.0453	0.0203	0.0793
**6-7-1-0**	0.0023	0.0063	0.0068	0.0060	0.0032	0.0050	0.0070	0.0051	0.0056	0.0055
**6-8-1-1**	0.0245	0.0210	0.0142	0.0050	0.0133	0.0143	0.0072	0.0223	0.0061	0.0138
**7-8-0-4**	0.0272	0.0230	0.0562	0.0221	0.0274	0.0196	0.0609	0.0204	0.0420	0.0287
**7-8-1-3**	0.0210	0.0185	0.0157	0.0131	0.0133	0.0289	0.0286	0.0292	0.0266	0.0245
**7-8-1-4**	0.0379	0.0265	0.0233	0.0186	0.0272	0.0341	0.0411	0.0348	0.0392	0.0318
**7-8-2-4 Iso 1**	0.0023	0.0021	0.0010	0.0004	0.0021	0.0033	0.0014	0.0029	0.0014	0.0024
**7-8-2-4 Iso 2**	0.0077	0.0090	0.0037	0.0019	0.0048	0.0099	0.0088	0.0089	0.0059	0.0080
**8-6-0-0**	3.6322	2.7986	4.8481	1.8808	2.8839	1.4835	1.4679	2.2826	1.3101	1.4193
**8-6-0-1 Iso 1**	0.5535	0.2892	0.8373	0.2294	0.3907	0.1806	0.1967	0.3780	0.2596	0.1745
**8-6-0-1 Iso 2**	0.7144	0.5615	0.8956	0.2485	0.6210	0.1662	0.3473	0.4422	0.3345	0.2375
**8-6-0-1 Iso 3**	0.4893	0.4471	0.2751	0.0820	0.3113	0.3238	0.1213	0.4754	0.1583	0.3087
**8-6-0-1 Iso 4**	0.0952	0.0608	0.0907	0.0432	0.0701	0.0420	0.0497	0.0601	0.0318	0.0398

Supplementary Table S6. MRM target list. GlcNAc, Hex, Fuc, NeuAc (*N*-acetylglucosamine, Hexose, Fucose, *N*-acetylneuraminic acid), and Iso = isomer.

Glycan	RT	Precursor ion (m/z)	Target ions
4-5-1-1 Iso 1	37.92	1300.178	312,344,376
4-5-1-1 Iso 2	44.57		
4-5-1-1 Iso 3	47.62		
5-6-1-0	53.47	1344.204	196, 432, 464
5-6-0-1 Iso 1	51.54	958.834	344, 376, 825
5-6-0-1 Iso 2	53.88		
4-5-1-2 Iso 1	42.61	987.513	312, 344, 376
4-5-1-2 Iso 2	44.93		
4-5-1-2 Iso 3	48.57		
4-5-1-2 Iso 4	51.96		
4-5-1-2 Iso 5	55.01		
4-5-2-2	47.5	1045.542	312, 344, 376
5-6-0-2 Iso 1	44.49	1079.225	344, 376, 825
5-6-0-2 Iso 2	48.71		
5-6-0-2 Iso 3	52.22		
5-6-0-2 Iso 4	55.51		
5-6-0-2 Iso 5	58.67		
5-6-1-2 Iso 1	43.19	1137.255	312, 344, 376
5-6-1-2 Iso 2	46.47		
5-6-1-2 Iso 3	51.51		
5-6-1-2 Iso 4	53.51		
5-6-1-2 Iso 5	59.37		
5-6-1-2 Iso 6	62.63		
5-6-0-3 Iso 1	44.87	1199.616	344, 376, 825
5-6-0-3 Iso 2	48.62		
5-6-0-3 Iso 3	52.49		
5-6-0-3 Iso 4	56.35		
5-6-0-3 Iso 5	61.97		
6-7-1-2	58.14	965.500	312, 344, 376
5-6-2-3 Iso 1	46.12	1315.676	312, 344, 376
5-6-2-3 Iso 2	55.13		
6-7-1-3 Iso 1	41.78	1055.793	312, 344, 376
6-7-1-3 Iso 2	45.76		
6-7-1-3 Iso 3	49.28		
6-7-1-3 Iso 4	51.97		
6-7-1-3 Iso 5	54.19		
6-7-1-3 Iso 6	56.89		
6-7-0-4 Iso 1	44.95	1102.564	344, 376, 825
6-7-0-4 Iso 2	48.71		
6-7-0-4 Iso 3	52.45		
6-7-0-4 Iso 4	53.86		
6-7-0-4 Iso 5	57.26		
6-7-0-4 Iso 6	60.18		
6-7-0-4 Iso 7	61.82		
6-7-0-4 Iso 8	66.27		
6-7-0-4 Iso 9	70.59		

Supplementary Table S7. MGC **MRM** analysis, normalized abundance of targeted *N*-glycans digested from depleted low abundant serum glycoproteins of **control** patients. GlcNAc, Hex, Fuc, NeuAc (*N*-acetylglucosamine, Hexose, Fucose, *N*-acetylneuraminic acid), and Iso = isomer.

Glycan	Control 1	Control 2	Control 3	Control 4	Control 5	Control 6	Control 7	Control 8	Control 9	Control 10
4-5-1-1 Iso 1	0.1012	0.2559	0.3087	0.3154	0.1132	0.2743	0.2933	0.1986	0.2205	0.2469
4-5-1-1 Iso 2	0.0546	0.2068	0.1575	0.2368	0.1658	0.1910	0.2128	0.1899	0.1999	0.2078
4-5-1-1 Iso 3	0.4704	0.9392	1.6520	0.8519	0.7150	0.9632	1.1038	0.9046	0.8487	0.8172
5-6-1-0	0.0070	0.0472	0.0536	0.0361	0.0486	0.0568	0.0535	0.0368	0.0270	0.0405
5-6-0-1 Iso 1	0.1467	1.8924	0.7835	0.7235	0.8191	1.1142	0.7781	0.7814	0.7301	1.1668
5-6-0-1 Iso 2	0.1060	1.4532	1.0313	0.9032	0.9648	1.2978	1.1014	1.0380	0.9729	1.2070
4-5-1-2 Iso 1	2.7133	2.7858	9.8218	8.6038	2.2191	5.5597	5.9546	5.1991	6.2966	5.3548
4-5-1-2 Iso 2	2.8674	2.6647	5.5112	3.6981	2.9414	3.3327	3.1832	3.2381	3.8764	3.3370
4-5-1-2 Iso 3	4.8137	2.3143	2.4795	2.6981	2.8387	2.1815	2.3025	2.9260	3.2539	2.6922
4-5-1-2 Iso 4	17.5707	13.6086	13.1960	20.5655	16.3000	16.4110	28.7636	26.2329	22.1335	19.8769
4-5-1-2 Iso 5	1.5953	1.2655	1.1367	2.5937	1.0715	1.3783	2.9414	1.8149	1.4096	1.2908
4-5-2-2	0.8191	0.1500	0.3339	0.4156	0.1392	0.2446	0.4919	0.3704	0.3942	0.2502
5-6-0-2 Iso 1	1.6873	0.6001	0.5707	0.3477	0.6004	0.4174	0.3497	0.4324	0.7370	0.5566
5-6-0-2 Iso 2	7.1062	9.5315	5.1911	4.2640	7.0852	6.7021	2.3210	4.0797	5.2673	5.7399
5-6-0-2 Iso 3	3.2526	3.1761	2.9529	1.9739	2.3711	2.7649	1.8995	2.0173	2.9451	2.6432
5-6-0-2 Iso 4	4.4759	11.8154	5.6051	6.4465	8.9811	9.0047	6.6065	6.5065	5.0226	8.7173
5-6-0-2 Iso 5	1.3367	4.1057	2.7895	2.8315	3.2129	4.0338	4.0837	3.0432	2.2179	3.8742
5-6-1-2 Iso 1	0.1965	0.3605	0.3781	0.3476	0.1941	0.2892	0.3184	0.2890	0.3686	0.2941
5-6-1-2 Iso 2	2.7029	3.9202	5.6702	3.6548	1.5107	5.3920	5.5771	3.0675	3.1821	3.8253
5-6-1-2 Iso 3	1.1897	3.3293	3.1967	3.2759	1.2481	4.0147	4.7760	2.7837	2.1862	3.3232
5-6-1-2 Iso 4	0.5063	0.9795	0.4739	0.8342	0.7863	0.8751	0.9021	0.7414	0.9344	0.8616
5-6-1-2 Iso 5	0.1612	0.3648	0.2482	0.4283	0.3265	0.5046	0.5570	0.4276	0.3676	0.4698
5-6-1-2 Iso 6	0.2611	0.5919	0.2296	0.4486	0.4996	0.5711	0.7422	0.5176	0.4773	0.5476
5-6-0-3 Iso 1	1.9555	2.2543	3.6219	3.1065	3.9571	2.1838	2.4709	3.2204	4.1318	3.1933
5-6-0-3 Iso 2	11.9300	3.6319	4.7230	4.1131	5.2395	3.4326	2.5434	4.1236	6.5530	4.4971
5-6-0-3 Iso 3	15.6984	11.9570	11.3680	10.0354	12.7836	9.9909	4.2706	10.0491	12.7712	10.3266
5-6-0-3 Iso 4	0.8490	1.0674	1.2318	1.1191	1.4256	1.4718	0.9864	1.2209	1.2935	1.3113
5-6-0-3 Iso 5	3.4637	5.5356	5.3055	6.8892	8.7757	6.5291	7.9624	6.8982	4.3559	6.2586
6-7-1-2	0.0601	0.1808	0.1777	0.1124	0.0535	0.1994	0.2715	0.0998	0.0592	0.1134
5-6-2-3 Iso 1	1.0944	0.2242	0.5516	0.4024	0.2034	0.3215	0.4102	0.2816	0.3242	0.2889
5-6-2-3 Iso 2	0.5106	0.2692	0.3784	0.7468	0.2466	0.4793	0.9877	0.5639	0.4545	0.3264
6-7-1-3 Iso 1	0.2567	0.1240	0.1613	0.1586	0.1211	0.1058	0.1986	0.1696	0.2147	0.1382
6-7-1-3 Iso 2	0.7319	0.1566	0.1918	0.1290	0.1754	0.1117	0.1561	0.1610	0.2234	0.1487
6-7-1-3 Iso 3	0.4395	0.2963	0.3057	0.1778	0.1578	0.2612	0.1516	0.1817	0.1822	0.1445
6-7-1-3 Iso 4	0.2479	0.3369	0.3380	0.2250	0.2602	0.3167	0.2350	0.2351	0.2119	0.2293
6-7-1-3 Iso 5	0.6272	0.7792	1.0111	0.6287	0.4244	0.9897	0.6910	0.4627	0.3376	0.4973
6-7-1-3 Iso 6	0.1873	0.3940	0.4601	0.3190	0.2092	0.4560	0.3531	0.2262	0.1910	0.2811
6-7-0-4 Iso 1	0.1948	0.1112	0.1117	0.1338	0.0669	0.0583	0.0795	0.1592	0.1706	0.0776
6-7-0-4 Iso 2	1.5033	0.2729	0.3379	0.2243	0.3558	0.1756	0.1320	0.2554	0.3706	0.2353
6-7-0-4 Iso 3	2.3276	1.6942	1.4903	1.3210	2.6057	1.0614	0.4652	1.1296	1.4886	1.2719
6-7-0-4 Iso 4	1.6656	1.4123	1.9763	1.0778	2.4758	1.3065	0.4079	1.1687	0.9307	0.9124
6-7-0-4 Iso 5	0.1677	0.2299	0.1516	0.2372	0.3139	0.1650	0.1261	0.1977	0.2356	0.2047
6-7-0-4 Iso 6	0.9512	1.3780	1.0529	1.0381	2.7266	1.4092	0.5473	1.0189	0.5809	0.9577
6-7-0-4 Iso 7	0.6799	0.8874	0.8321	0.9132	1.3525	0.8559	0.7726	0.8066	0.5508	0.8532
6-7-0-4 Iso 8	0.1022	0.1353	0.2295	0.1215	0.2635	0.1467	0.0925	0.1812	0.1257	0.1071
6-7-0-4 Iso 9	0.2138	0.3124	0.2200	0.2752	0.6540	0.3971	0.3749	0.3523	0.1731	0.2847

Supplementary Table S8. MGC **MRM** analysis, normalized abundance of targeted *N*-glycans digested from depleted low abundant serum glycoproteins of **MCI** patients. GlcNAc, Hex, Fuc, NeuAc (*N*-acetylglucosamine, Hexose, Fucose, *N*-acetylneuraminic acid), and Iso = isomer.

Glycan	MCI 1	MCI 2	MCI 3	MCI 4	MCI 5	MCI 6	MCI 7	MCI 8	MCI 9	MCI 10
4-5-1-1 Iso 1	0.1533	0.1613	0.0615	0.1226	0.0558	0.2407	0.0577	0.2139	0.1184	0.1975
4-5-1-1 Iso 2	0.1843	0.1534	0.1085	0.1730	0.1426	0.1468	0.0766	0.1439	0.1204	0.1854
4-5-1-1 Iso 3	0.4470	0.6760	0.4132	0.5440	0.4339	0.5773	0.3099	0.4927	0.5294	0.5966
5-6-1-0	0.0417	0.0336	0.0255	0.0263	0.0195	0.0280	0.0265	0.0229	0.0197	0.0329
5-6-0-1 Iso 1	1.3029	1.2355	0.8462	0.7902	0.6560	1.3090	0.5456	0.6989	0.6456	0.7315
5-6-0-1 Iso 2	0.7908	0.8962	0.5968	0.7389	0.8591	1.4605	0.5982	1.2922	0.8582	1.3562
4-5-1-2 Iso 1	4.5259	3.8026	1.0395	3.4047	1.2107	5.8390	2.2176	6.0728	3.2032	6.4081
4-5-1-2 Iso 2	3.7046	2.8189	2.1634	2.7373	2.8170	3.1978	2.7200	2.8413	2.9374	4.3421
4-5-1-2 Iso 3	2.4440	2.1372	1.9096	2.5396	2.2092	2.2873	2.1727	2.7402	2.9208	2.8342
4-5-1-2 Iso 4	6.1095	15.7739	9.8225	13.7854	9.6373	17.0254	12.3921	17.7690	19.3630	16.1405
4-5-1-2 Iso 5	0.4826	0.9606	0.6843	1.0950	0.3485	0.9669	1.0095	1.4497	1.3402	1.2903
4-5-2-2	0.1302	0.1907	0.0759	0.1728	0.1659	0.2714	0.1165	0.2641	0.1978	0.2994
5-6-0-2 Iso 1	1.1900	0.6355	0.8581	0.7627	0.8032	0.6228	0.4879	0.3770	0.4419	0.6072
5-6-0-2 Iso 2	9.8121	7.7603	9.6571	6.3202	8.2334	6.3602	6.6715	4.8419	6.2951	5.2539
5-6-0-2 Iso 3	3.8227	2.4566	2.3298	2.3326	3.2634	3.7293	2.4275	2.8497	2.1906	3.3416
5-6-0-2 Iso 4	9.2565	9.1565	9.7493	7.7686	9.7452	7.6926	6.8111	7.0739	6.7845	5.6123
5-6-0-2 Iso 5	2.3691	3.1977	2.2601	2.5180	3.2413	3.5949	2.1555	2.8586	2.0603	2.6172
5-6-1-2 Iso 1	0.1828	0.2375	0.1083	0.1078	0.0815	0.2535	0.0728	0.0927	0.1011	0.1430
5-6-1-2 Iso 2	3.6923	3.5949	1.0704	1.8380	1.0070	5.0998	1.4222	2.4137	1.5292	3.1744
5-6-1-2 Iso 3	2.4441	2.3991	0.7746	1.4483	0.9189	3.6894	1.0050	2.3165	1.2151	2.1849
5-6-1-2 Iso 4	0.5852	0.7499	0.6487	0.6233	0.5785	0.9242	0.6848	0.6802	0.7942	0.7815
5-6-1-2 Iso 5	0.3104	0.3229	0.1748	0.3050	0.2626	0.4638	0.2521	0.3465	0.3119	0.3796
5-6-1-2 Iso 6	0.3147	0.4308	0.4793	0.3809	0.3498	0.4007	0.3824	0.4460	0.5810	0.3611
5-6-0-3 Iso 1	3.5504	3.1653	2.2447	3.1496	2.7505	3.2395	2.7179	2.7126	2.3861	3.0792
5-6-0-3 Iso 2	7.2061	4.3569	5.5628	5.1448	6.9903	5.3900	6.8694	4.9107	4.6418	5.0388
5-6-0-3 Iso 3	17.8661	15.6229	26.9837	20.2701	23.8423	11.0626	24.5374	15.3635	20.4911	14.3311
5-6-0-3 Iso 4	1.3204	1.1750	1.1920	1.8060	1.8931	1.3782	1.5071	1.6373	1.0224	1.6113
5-6-0-3 Iso 5	4.6605	7.4592	8.1253	9.2615	7.9577	5.5689	7.6740	8.1606	7.5416	6.7725
6-7-1-2	0.0727	0.0673	0.0237	0.0603	0.0328	0.1355	0.0271	0.0799	0.0489	0.0951
5-6-2-3 Iso 1	0.3099	0.2828	0.1216	0.2437	0.2097	0.2715	0.1154	0.2862	0.1693	0.3952
5-6-2-3 Iso 2	0.1481	0.4228	0.2117	0.2927	0.1782	0.3704	0.2919	0.4705	0.4568	0.6878
6-7-1-3 Iso 1	0.1710	0.1152	0.1088	0.1455	0.1059	0.1660	0.1085	0.1223	0.1462	0.2150
6-7-1-3 Iso 2	0.3096	0.1790	0.1895	0.2465	0.2299	0.2284	0.1370	0.1431	0.1476	0.2757
6-7-1-3 Iso 3	0.3060	0.1745	0.1185	0.2036	0.2848	0.3068	0.1663	0.2346	0.2046	0.3170
6-7-1-3 Iso 4	0.4021	0.3133	0.2994	0.2066	0.2100	0.7287	0.3568	0.5613	0.3401	0.6760
6-7-1-3 Iso 5	0.6432	0.4787	0.2056	0.3493	0.1433	0.3914	0.1535	0.2869	0.1953	0.3653
6-7-1-3 Iso 6	0.4111	0.2694	0.1499	0.1841	0.1149	0.2131	0.1122	0.1854	0.1359	0.2108
6-7-0-4 Iso 1	0.1821	0.1655	0.1481	0.1863	0.1780	0.1116	0.0948	0.1065	0.1074	0.1377
6-7-0-4 Iso 2	0.5579	0.2968	0.4017	0.4136	0.5804	0.2794	0.4212	0.3102	0.3762	0.4433
6-7-0-4 Iso 3	2.3711	1.8278	2.7814	1.8217	2.2472	1.0225	2.4066	1.5630	2.1566	1.5187
6-7-0-4 Iso 4	2.4961	1.1029	1.9090	1.6779	1.7257	0.8557	2.9519	1.3818	1.7181	1.8053
6-7-0-4 Iso 5	0.1799	0.2574	0.3519	0.3762	0.3337	0.2268	0.3789	0.2959	0.3750	0.3030
6-7-0-4 Iso 6	1.2482	1.1107	1.4519	1.6482	1.4673	0.8689	2.4208	1.3244	1.3505	1.3208
6-7-0-4 Iso 7	0.8546	0.9127	1.0186	1.0958	0.9145	0.6198	1.1607	0.9951	0.9648	0.9706
6-7-0-4 Iso 8	0.2052	0.1612	0.2101	0.2049	0.1704	0.1143	0.2526	0.1797	0.1605	0.2060
6-7-0-4 Iso 9	0.2309	0.3012	0.3326	0.4760	0.3990	0.2690	0.5223	0.3901	0.3042	0.3523

Supplementary Table S9. List of glycoproteins observed in the proteomic analysis of the low abundant serum glycoproteins from depleted serum samples of control and MCI patients.

No.	T: Protein IDs	Gene names	LFQ Intensity Average Control	LFQ Intensity Average MCI	Ratio Control/MCI	Fold change	*p* value
1	O00391	QSOX1	25.380	25.184	0.992	-0.011	0.5524
2	O75882	ATRN	28.175	28.315	1.005	0.007	0.0788
3	O95497	VNN1	24.131	23.383	0.969	-0.045	0.0475
4	P00450	CP	34.155	34.316	1.005	0.007	0.1077
5	P00734	F2	32.169	32.235	1.002	0.003	0.3615
6	P00736	C1R	30.324	30.381	1.002	0.003	0.3660
7	P00740	F9	27.743	27.780	1.001	0.002	0.3872
8	P00742	F10	27.246	27.057	0.993	-0.010	0.4535
9	P00747	PLG	32.822	32.892	1.002	0.003	0.2732
10	P00748	F12	29.097	29.287	1.007	0.009	0.1203
11	P00751	CFB	33.094	33.113	1.001	0.001	0.4515
12	P01008	SERPINC1	31.351	31.416	1.002	0.003	0.2910
13	P01011	SERPINA3	32.701	32.444	0.992	-0.011	0.1094
14	P01019	AGT	31.195	31.343	1.005	0.007	0.1620
15	P01031	C5	31.266	31.225	0.999	-0.002	0.7224
16	P01042	KNG1	32.600	32.676	1.002	0.003	0.2077
17	P02649	APOE	29.896	29.375	0.983	-0.025	0.0022
18	P02671	FGA	28.382	28.339	0.998	-0.002	0.7563
19	P02743	APCS	30.800	30.852	1.002	0.002	0.7460
20	P02745	C1QA	27.600	26.861	0.973	-0.039	0.0843
21	P02748	C9	30.942	30.861	0.997	-0.004	0.9590
22	P02749	APOH	32.505	32.739	1.007	0.010	0.0414
23	P02750	LRG1	30.006	30.020	1.000	0.001	0.8199
24	P02751	FN1	32.869	32.692	0.995	-0.008	0.3621
25	P02760	AMBP	31.224	31.229	1.000	0.000	0.9246
26	P02766	TTR	27.098	26.906	0.993	-0.010	0.9351
27	P02790	HPX	34.829	35.035	1.006	0.009	0.0293
28	P03951	F11	24.901	24.674	0.991	-0.013	0.6699
29	P03952	KLKB1	29.114	29.094	0.999	-0.001	0.5277
30	P04003	C4BPA	28.870	27.979	0.969	-0.045	0.2105
31	P04004	VTN	31.363	31.359	1.000	0.000	0.6161
32	P04070	PROC	25.612	25.489	0.995	-0.007	0.4458
33	P04114	APOB	33.446	33.056	0.988	-0.017	0.0294
34	P04180	LCAT	23.308	22.610	0.970	-0.044	0.1734
35	P04196	HRG	30.951	31.219	1.009	0.012	0.0483
36	P04217	A1BG	32.680	32.804	1.004	0.005	0.1506
37	P04275	VWF	28.019	27.878	0.995	-0.007	0.7169
38	P04278	SHBG	26.351	26.781	1.016	0.023	0.1142
39	P05090	APOD	24.405	24.033	0.985	-0.022	0.2599
40	P05154	SERPINA5	23.585	23.904	1.014	0.019	0.2592
41	P05155	SERPING1	29.751	29.612	0.995	-0.007	0.6020
42	P05156	CFI IF	30.170	30.265	1.003	0.005	0.6421
43	P05160	F13B	27.733	28.013	1.010	0.015	0.1438
44	P05362	ICAM1	22.679	22.497	0.992	-0.012	0.3181
45	P05543	SERPINA7	28.528	28.830	1.011	0.015	0.0254
46	P05546	SERPIND1	30.211	30.286	1.002	0.004	0.3817
47	P06276	BCHE	27.388	27.593	1.007	0.011	0.1915
48	P06681	C2	29.159	29.237	1.003	0.004	0.6170
49	P07225	PROS1	27.550	26.901	0.976	-0.034	0.0272
50	P07357	C8A	29.057	29.047	1.000	0.000	0.6972
51	P07358	C8B	29.163	29.063	0.997	-0.005	0.9101
52	P07359	GP1BA	23.956	23.556	0.983	-0.024	0.4152
53	P08185	SERPINA6	27.568	27.504	0.998	-0.003	0.6108
54	P08571	CD14	25.965	25.840	0.995	-0.007	0.9496
55	P08603	CFH	33.425	33.333	0.997	-0.004	0.3842
56	P08697	SERPINF2	29.524	29.594	1.002	0.003	0.7635
57	P09172	DBH	24.531	25.099	1.023	0.033	0.3020
58	P09486	SPARC	24.855	24.947	1.004	0.005	0.4958
59	P09871	C1S	29.716	29.745	1.001	0.001	0.5469
60	P0C0L4	C4A	29.367	28.506	0.971	-0.043	0.0094
61	P0C0L5	C4B	34.600	34.332	0.992	-0.011	0.2134
62	P10643	C7	29.926	30.050	1.004	0.006	0.3704
63	P10909	CLU	30.811	30.706	0.997	-0.005	0.8420
64	P12259	F5	26.876	26.905	1.001	0.002	0.6579
65	P13591	NCAM1	22.876	23.087	1.009	0.013	0.6527
66	P13598	ICAM2	23.221	23.194	0.999	-0.002	0.9442
67	P13671	C6	30.177	30.287	1.004	0.005	0.3813
68	P14151	SELL	24.718	24.906	1.008	0.011	0.2074
69	P17936	IGFBP3	24.314	25.403	1.045	0.063	0.0011
70	P18428	LBP	24.968	24.795	0.993	-0.010	0.7932
71	P19320	VCAM1	23.261	23.050	0.991	-0.013	0.1231
72	P19823	ITIH2	32.611	32.518	0.997	-0.004	0.8170
73	P19827	ITIH1	32.097	32.014	0.997	-0.004	0.9061
74	P20851	C4BPB	23.855	23.515	0.986	-0.021	0.7141
75	P22792	CPN2	28.761	29.036	1.010	0.014	0.0422
76	P22891	PROZ	25.036	25.171	1.005	0.008	0.2484
77	P23142	FBLN1	26.537	27.200	1.025	0.036	0.0697
78	P25311	AZGP1	32.135	32.313	1.006	0.008	0.1197
79	P26927	MST1	26.009	26.324	1.012	0.017	0.1745
80	P27169	PON1	24.299	25.287	1.041	0.058	0.0832
81	P27918	CFP	26.170	25.706	0.982	-0.026	0.2548
82	P29622	SERPINA4	28.348	28.683	1.012	0.017	0.0378
83	P35858	IGFALS	28.701	29.135	1.015	0.022	0.0654
84	P36955	SERPINF1	30.220	29.866	0.988	-0.017	0.2427
85	P36980	CFHR2	26.418	26.950	1.020	0.029	0.3546
86	P43121	MCAM	23.799	24.321	1.022	0.031	0.0246
87	P43251	BTD	26.179	26.406	1.009	0.012	0.1289
88	P43652	AFM	31.498	31.474	0.999	-0.001	0.8577
89	P48740	MASP1	25.755	25.882	1.005	0.007	0.3931
90	P49747	COMP	26.447	26.467	1.001	0.001	0.5965
91	P49908	SELENOP	26.864	26.763	0.996	-0.005	0.9790
92	P51884	LUM	29.560	29.419	0.995	-0.007	0.7527
93	P68871	HBB	23.840	23.347	0.979	-0.030	0.2814
94	P80108	GPLD1	24.885	24.894	1.000	0.001	0.6491
95	P98160	HSPG2	22.739	22.701	0.998	-0.002	0.7015

Supplementary Table S9. Continued…

No.	T: Protein IDs	Gene names	LFQ Intensity Average Control	LFQ Intensity Average MCI	Ratio Control/MCI	Fold change	*p* value
96	Q03591	CFHR1	27.852	27.759	0.997	-0.005	0.7823
97	Q04756	HGFAC	27.182	27.151	0.999	-0.002	0.8986
98	Q06033	ITIH3	29.043	28.685	0.988	-0.018	0.2828
99	Q08380	LGALS3BP	24.391	24.021	0.985	-0.022	0.4550
100	Q12805	EFEMP1	24.561	24.354	0.992	-0.012	0.9996
101	Q14515	SPARCL1	23.012	22.769	0.989	-0.015	0.4595
102	Q14520	HABP2	27.415	27.290	0.995	-0.007	0.7102
103	Q14624	ITIH4	32.505	32.302	0.994	-0.009	0.1374
104	Q16610	ECM1	27.884	27.921	1.001	0.002	0.9531
105	Q4G0P3	HYDIN	27.993	27.149	0.970	-0.044	0.8984
106	Q6EMK4	VASN	24.897	24.481	0.983	-0.024	0.0402
107	Q6UXB8	PI16	24.975	24.200	0.969	-0.046	0.1056
108	Q86VB7	CD163	23.537	23.426	0.995	-0.007	0.4753
109	Q8IUB2	WFDC3	26.808	26.739	0.997	-0.004	0.5431
110	Q92820	GGH	24.829	24.813	0.999	-0.001	0.5991
111	Q92954	PRG4	24.795	23.942	0.966	-0.050	0.0011
112	Q96IY4	CPB2	26.115	26.394	1.011	0.015	0.4123
113	Q96KN2	CNDP1	24.554	25.993	1.059	0.082	0.1296
114	Q96PD5	PGLYRP2	29.632	29.811	1.006	0.009	0.0925
115	Q9BXR6	CFHR5	23.680	23.550	0.995	-0.008	0.9096
116	Q9NZP8	C1RL	25.460	25.969	1.020	0.029	0.1242
117	Q9UGM5	FETUB	25.159	25.334	1.007	0.010	0.5583
118	Q9UK55	SERPINA10	25.225	25.582	1.014	0.020	0.0829
119	Q9UNN8	PROCR	22.730	22.815	1.004	0.005	0.8216
120	Q9Y6R7	FCGBP	26.110	25.783	0.987	-0.018	0.8940
